# Role of Hydrogen Bonds in Formation of Co-amorphous Valsartan/Nicotinamide Compositions of High Solubility and Durability with Anti-hypertension and Anti-COVID-19 Potential

**DOI:** 10.1021/acs.molpharmaceut.0c01096

**Published:** 2021-04-01

**Authors:** Marika Turek, Ewa Różycka-Sokołowska, Marek Koprowski, Bernard Marciniak, Piotr Bałczewski

**Affiliations:** † Institute of Chemistry, Faculty of Science and Technology, Jan Długosz University in Częstochowa, Armii Krajowej 13/15, Częstochowa 42-201, Poland; ‡ Division of Organic Chemistry, Centre of Molecular and Macromolecular Studies, Polish Academy of Sciences, Sienkiewicza 112, Łódź 90-363, Poland

**Keywords:** anti-hypertensive, COVID-19, co-amorphization, nicotinamide, solid dispersion, valsartan

## Abstract

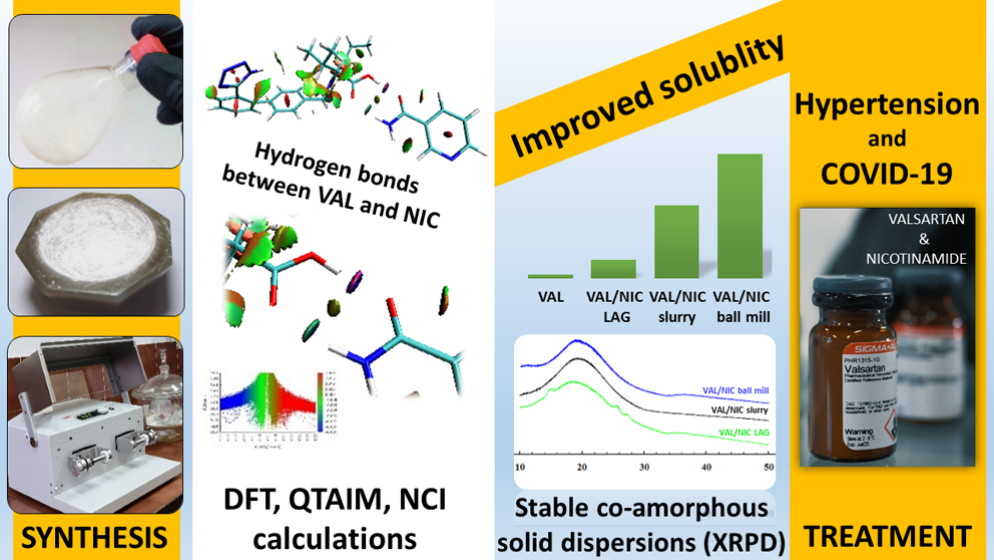

Physicochemical properties, in particular solubility and the associated bioavailability, are key factors in determining efficacy of poorly water-soluble drugs, which constitute 40% of new drugs in the market, and improving them is an important challenge for modern pharmacy. A recent strategy to achieve this goal is formation of stable co-amorphous solid dispersions with co-formers of low molecular weight. Here, the amorphization strategy was applied for low-soluble anti-hypertensive valsartan (VAL), an angiotensin II receptor blocker, and nicotinamide, which exhibits lung- and cardio-protective effects. Through interactions with the renin–angiotensin–aldosteron system, VAL may be used to treat both hypertension and the current pandemic coronavirus SARS-CoV-2 infection. Using mechanochemical and liquid- and solid-state approaches, solvated co-amorphous solid dispersions of VAL with nicotinamide were obtained. They were characterized by spectroscopic, thermal, and X-ray analyses. The density functional theory, quantum theory of atoms in molecules, and non-covalent interaction index calculations revealed the presence of two types of hydrogen bonds between VAL and NIC (i.e., N–H···O and O–H···O). One of them had a partially covalent character, which caused conformational changes in the flexible VAL molecule, restricting contribution of the tetrazolyl N–H donor and thus limiting the possibility of co-crystal formation. The recognized VAL/NIC1- and VAL/NIC2-type heterodimeric interactions were responsible for the excellent durability of the solid compositions and up to 24-fold better solubility than VAL alone. The synthesized dispersions constitute a new class of dually acting drugs, containing an active pharmaceutical ingredient (VAL) and supporting nutraceutical (nicotinamide).

## 1 Introduction

Valsartan (VAL) is an anti-hypertensive drug belonging to the angiotensin II receptor blockers (ARBs), which according to the recommendations of the European Cardiac Society (2016) provide alternative treatment for patients intolerant of angiotensin-converting enzyme inhibitors (ACE inhibitors) or aldosterone receptor blocker therapies.^1^ High blood pressure is one of the most important risk factors for cardiovascular diseases, and hence, ARBs are an important class of drugs decreasing the risk of heart diseases.^2^ However, ARBs are poorly water-soluble drugs, classified according to the Biopharmaceutics Classification System (BCS) as class II with high permeability and low solubility.^3^ The bioavailability of BSC class II drugs is limited by their solvation rate and in the case of ARBs, it ranges from 13% (eprosartan) to 70% (irbesartan), and for the title, VAL is 23%. No wonder that pharmaceutical industry is intensively looking for solutions that will allow them to reduce the dose of the drugs by enhancing solubility, which improves their bioavailability. Several strategies have been used to improve the oral bioavailability of VAL, such as co-crystallization,^4−7^ formation of host–guest β-cyclodextrin complexes,^8,9^ synthesis of microcapsules,^10^ nanoparticles,^11^ and polymeric solid dispersions.^12−14^ It is worth noting that the solid dispersion strategy leading to amorphization of pharmaceutical components is especially important in the case of pharmaceuticals, such as VAL, which are difficult to crystallize, and attempts to crystallize them usually lead to a sticky solid, amorphous film, or loose powder.^15^

Generally, amorphous forms of drugs dissolve more rapidly and are better absorbed than the corresponding crystalline forms.^16^ Hence, amorphization gives an opportunity to improve dissolution rates of drugs, which directly leads to the increase in their bioavailability.^17^ The amorphous solids (glasses) in contrast to the crystalline solids have no well-defined molecular arrangement, but their enhanced molecular mobility leads to a better solubility, providing the advantage to use this type of active pharmaceutical ingredients (APIs).^18^ They possess higher free energy and entropy than the corresponding crystals and tend to recrystallize to lower energy crystalline forms, so that special methods, such as the solid dispersion strategy, are necessary to stabilize the amorphous state. The solid dispersion is defined as a dispersion of one or more active substances suspended in an inert carrier and prepared by melting, dissolution, or melting–dissolution techniques.^19^ To date, polymeric amorphous dispersions containing the drug incorporated in a glass polymeric matrix have commonly been used for stabilization of amorphous drugs. However, such solid dispersions have several disadvantages, including low miscibility necessitating large polymer/drug ratios and hygroscopicity.^17^ Therefore, a new alternative strategy, leading to co-amorphous solid dispersions, in which the polymer was replaced by a low-molecular weight substance, such as nicotinamide (NIC), has been developed. These mixtures combine two or more low-molecular weight components, called as co-formers, into a homogenous amorphous single phase.^20^ Addition of a co-former gives advantages, such as preventing recrystallization of the amorphous API and preventing phase separation observed in polymeric matrices. The solid dispersions can be prepared using milling (ball milling and cryo-milling), solvent evaporation (rotary evaporation, spray drying, and freeze drying), quench cooling, and hot-melt extrusion techniques.^21^

Some of these techniques have been applied to improve solubility of VAL; however, polymer-based glass solutions were mainly obtained.^12−14,22−24^ Mei et al. reported on the co-amorphous mixture where amino acids were used as low molecular excipients to increase the solubility of VAL up to 1000 times; however, some of these solid dispersions turned out to be unstable after 3 months.^25^ Other solid dispersions of VAL with small molecular co-formers have also been reported: (a) with inorganic, alkalizing agents (CaCO_3_) and (b) organic *N*-methyl-d-glucamine. A 46-fold and ninefold increase in solubility, respectively, has been achieved.^26^ A co-amorphous system of VAL with vanillin, loaded in mesoporous silica particles with an improved dissolution rate, has also been described.^27^ However, the co-formers applied in the abovementioned API-GRAS (GRAS—generally recognized as safe, FDA list of safe food additives) systems do not contribute to cardiovascular health effects. There are only two, recently described, co-amorphous API–API systems containing VAL, obtained by Shastri et al.^28,29^ First, the VAL–clinidipine system turned out to be stable under standardized accelerated conditions (40 °C/75% RH) for 1 month and exhibited an enhanced dissolution profile.^28^ The second system, VAL–nifedipine, showed the same stability as the VAL–clinidipine system and 2.19-fold-increased solubility compared to the free VAL.^29^

**Figure 1:**
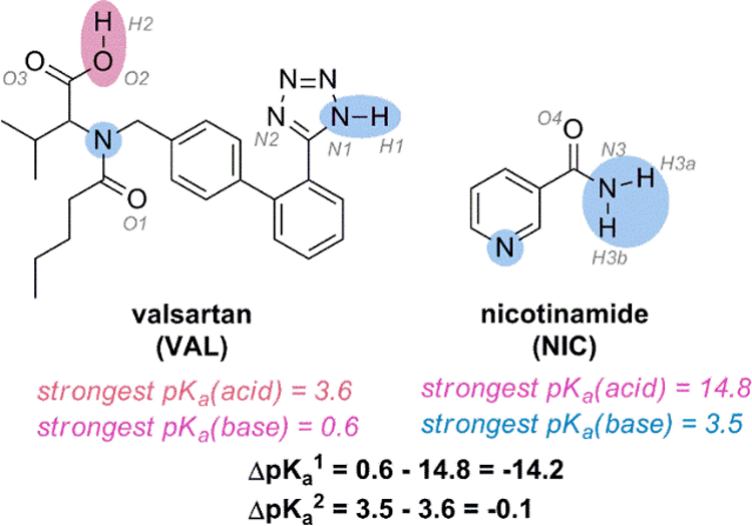
Chemical structures of VAL and NIC with predicted pKa values (ACD Lab Percepta software). Functional groups which can form non-covalent bonds and their atom numbering are marked in colors.

The current paper is focused on co-amorphous formulations, consisting of VAL (as the API) and NIC, as pharmaceutically acceptable, small-molecular weight excipient, which has been chosen in view of its high water solubility and beneficial health effect. NIC, which is one of the two components of vitamin B_3_ (PP), exhibits several beneficial health effects, among others: (1) supports the cardiovascular system (promotes relaxation and reduces anxiety), (2) stimulates the immune system (*vide infra*), (3) reduces inflammation [prevents lung tissue damage and slows the progress of degenerative arthritis (*vide infra*)]. Hence, it can be rationally used as the excipient of the VAL composition.^30^ Both VAL and NIC contain several functional groups, which are part of the relevant synthons needed to create strong intermolecular interactions, for example, carboxyl, tetrazolyl, and amide groups (VAL) and amide and pyridyl groups (NIC) (Figure 1). The Δp*K*_a_ values also indicate the possibility of creating VAL–NIC compositions with strong intermolecular bonds. Based on a generally accepted “rule of three”, the co-crystal formation (lack of proton transfer) is expected for Δp*K*_a_ < 0 and the salt formation (proton transfer occurs) is expected for Δp*K*_a_ > 3.^31^ The Δp*K*_a_ values of the investigated VAL/NIC system are <0; therefore, hydrogen bonding instead of salt formation is expected (Figure 1). Moreover, the 1:1 molar ratio (VAL/NIC) used fully corresponded to the recommended daily dosages of VAL (80–320 mg, therapeutic dose) and NIC (20–50 mg, preventive supplementation dose). Therefore, this type of formulation can be used to treat hypertension as an improved, more bioavailable, and dually acting composition.

ARB drugs (e.g., VAL) target the renin–angiotensin–aldosteron system by blocking angiotensin II type 1 receptors (AT1R).^32−39^ Based on ClinicalTrials.gov resources, 57 clinical trials regarding the influence of ARBs in the treatment of the COVID-19 disease have been commenced until October 2020. Five of these clinical trials are focused only on VAL and one (ClinicalTrials ID: NCT04335786) proposes two mechanisms: (1) ARBs block the excessive angiotensin-mediated AT1R activation; (2) ARBs upregulate ACE2, which reduces angiotensin II concentrations and increases the production of the protective vasodilator angiotensin-(1–7). NIC, which is the second component of the solid dispersion, can also be useful in the treatment of the COVID-19 disease. It has been shown that NIC, apart from the beneficial influence on the cardiovascular system, prevents lung tissue damage^40^ and is a key compound that enhances immune response, especially in viral infections.^41^ It has a strong anti-inflammatory effect on respiratory lung damage, which is crucial for COVID-19 patient treatment.^42,43^

In this work, four co-amorphous compositions of VAL and NIC were synthesized by the solid- and solution-state protocols. Solid dispersions and their components (VAL and NIC) have been studied by Fourier transform infrared (FT-IR) spectroscopy supported by the calculations in the framework of density functional theory (DFT), Bader’s quantum theory of atoms in molecules (QTAIM),^44^ and the non-covalent interactions index (NCI) method,^45^ which can be treated as an extension of QTAIM. It is a very useful tool for distinction and visualization of non-covalent interactions of different types. Bader’s QTAIM has been widely applied to study covalent and non-covalent interactions.^46−52^ However, in the area of multicomponent pharmaceutical solid forms, these calculations have rarely been used,^53^ despite the fact that non-covalent bonds play a crucial role in the formation of co-crystals or co-amorphous solid dispersions and hence in changing properties of these solid forms.^17,18^ Here, we employed QTAIM and NCI to elucidate the strength and nature of the possible non-covalent interactions occurring in VAL/NIC co-amorphous solid dispersions and compared them with the interactions occurring in single-component crystals. Additional calculations of solvation free energy were utilized to investigate the interactions between the solute (co-amorphous solid dispersions) and solvents (water and ethanol). Other experimental techniques, such as solid-state NMR (ssNMR), X-ray powder diffraction (XRPD), differential scanning calorimetry (DSC), and scanning electron microscopy (SEM), have also been applied to confirm amorphization of the obtained materials. The solubility of co-amorphous formulations was investigated in water and phosphate buffer (pH 7.4) and compared with the solubility of pure VAL.

## 2 Materials and Methods

### 2.1 Materials

VAL was extracted from Vanatex (Polpharma SA) and Axudan (Sandoz GmbH). The detailed procedure and analyses are described in the Supporting Information. Briefly, VAL was extracted from tablets with acetonitrile and purified by the slurry crystallization method developed by Mei et al.^15^ The structure of the API obtained was confirmed by spectroscopic (NMR, MS, and IR), thermal (DSC and melting point) and X-ray (XRPD) analyses (SM). NIC and solvents were purchased from Sigma-Aldrich and used without further treatment.

### 2.2 Synthesis of Co-Amorphous Mixtures

(1) VAL/NIC LAGs: equimolar amounts (1 mmol) of the two components (VAL and NIC) were ground using an agate mortar and pestle with addition of six drops of ethanol per each 30 min with a total grinding time of 30 min (VAL/NIC LAG 30 min) and 60 min (VAL/NIC LAG 60 min). The resulted powder was dried and kept in a desiccator. (2) VAL/NIC slurry: equimolar amounts (1 mmol) of the two components (VAL and NIC) were added to 3 mL of ethanol and the resulted slurry was stirred and refluxed at 70 °C for 3 h. The solvent was evaporated on a rotary vacuum evaporator. The solid obtained was dried and stored in a desiccator. (3) VAL/NIC ball mill: equimolar amounts (1 mmol) of the two components (VAL and NIC) were placed in a stainless-steel 12 mL grinding jar with 10 stainless-steel balls (4 mm diameter) and ground with addition of 12 drops of ethanol at 450 rpm for 60 min. The obtained sticky solid was dissolved in anhydrous ethanol, which was then evaporated on a rotary vacuum evaporator. The solid obtained was dried for 30 min under vacuum (0.7 mbar) and stored in a desiccator.

### 2.3 Fourier Transform Infrared Spectroscopy

The Fourier transform mid infrared spectra of VAL and NIC and the obtained co-amorphous mixtures were measured at 2 cm^–1^ resolution and by 32 scans on a Nicolet-Nexus spectrometer, in the region of 4000–400 cm^–1^, using the KBr pellet technique. Vibrations of the essential groups, which may be involved in the hydrogen bond formation, have carefully been analyzed. The data were processed using Spectrum software.

### 2.4 DFT, QTAIM, and NCI Calculations

DFT calculations were performed with the Gaussian 09 set of programs.^54^ The structures of components (VAL and NIC) and two designed VAL/NIC heterodimers (VAL/NIC1 and VAL/NIC2) differing in the existing supramolecular synthons were optimized and the IR intensities were calculated with the hybrid functional B3LYP combined with the 6-311++G(d,p) basis set. These two heterodimers (VAL/NIC1 and VAL/NIC2) were selected after preliminary calculations of five different heterodimers using the semiempirical AM1 method, as the two heterodimers with the lowest energy (Figure S10). This approach allowed us to assess how the arrangement of components (VAL and NIC) in the co-amorphous formulation affected the shift of vibrations of essential functional groups involved in the formation of hydrogen bonds. To compare predicted and observed frequencies, the wavenumbers were scaled by 0.967.^55^ Potential energy distribution analysis was performed using the FCART program.^56^ QTAIM and NCI calculations were performed for the designed heterodimers with the structures optimized at the B3LYP/6-311++G(d,p) level both in the gas phase and in ethanol. Additionally, these calculations were conducted for VAL/VAL homodimers and NIC/NIC homotrimers to investigate binding energies in individual component crystals. Therefore, homodimers/trimers were cut from the crystal structures of NIC (Cambridge Structural Database (CSD) refcode NICOAM06) and VAL (CSD refcode KIPLIG), and the single-point DFT calculations were performed. To investigate the intermolecular interactions between ethanol and the investigated VAL/NIC system, these calculations were also carried out for the designed solvated system (VAL/NIC1)EtOH and crystal solvate of VAL cut from the crystal structure (CSD refcode KIPLEC).

The calculations in the framework of QTAIM were performed with the use of Multiwfn (a multifunctional wavefunction analyzer) software.^57^ It enabled us to find the bond critical points (BCPs) of non-covalent interactions and to determine the properties of electron density at these points, such as the electron density at BCP (ρ_BCP_), its Laplacian (∇^2^ρ_BCP_), the kinetic (*G*_BCP_), potential (*V*_BCP_), and total (*H*_BCP_) electron energy densities. The energies of non-covalent interactions (*E*_bin_) were calculated from the *V*_BCP_ values, according to the equation in the form of *E*_bin_ = *V*_BCP_/2, which was proposed by Espinosa et al.^58^ To be able to state that a particular non-covalent H···A interaction belongs to the group of hydrogen bonds, we have used the first four criteria proposed by Koch and Popelier (KP).^59^ According to them, the hydrogen bond occurs when (i) the bond path between donor (D) and acceptor (A) atoms exists, (ii) the ρ_BCP_ values lie in the range of 0.002–0.034 a.u., (iii) the ∇^2^ρ_BCP_ values lie in the range from 0.024 to 0.139 a.u., and (iv) a mutual penetration of the H and A atoms, which is treated as “a necessary and sufficient” criterion, exists (i.e., Δ*r*_H_ = *r*_H_^V^ – *r*_H_ > Δ*r*_A_ = *r*_A_^V^ – *r*_A_ and Δ*r*_H_ + Δ*r*_A_ > 0, where *r*_H_^V^ and *r*_A_^V^ are the van der Waals radii of H and A atoms, respectively, and *r*_H_ and *r*_A_ denote the distances between these atoms and BCP). Multiwfn software was also used for calculations in the framework of the NCI method, which can be treated as an extension of QTAIM theory. This method is based on the relationship between the electron density (ρ) and the reduced density gradient (RDG = [1/2(3π^2^)^1/3^][|∇ρ|/ρ^4/3^]) and is a very useful tool for the distinction and visualization of different types of non-covalent interactions. The NCI calculations were visualized with VMD (visual molecular dynamics) software^60^ and Gnuplot 5.2.^61^

Free energies of solvation, its components (cavitation, dispersion, non-electrostatic, electrostatic, and polarization energy), and dipole moments were calculated for both heterodimers (VAL/NIC1 and VAL/NIC2) and the solvated system (VAL/NIC1)EtOH with the polarizable continuum model (PCM) with the integral equation formalism variant at the B3LYP/6-311++G(d,p) level of theory with the Gaussian 09 package and compared with the results obtained in the gas phase.^54^ The solute cavity was created *via* overlapping of van der Waals spheres, with water and ethanol as solvents.

### 2.5 Solid-State NMR

The ^13^C cross-polarization magic angle spinning (^13^C CP-MAS) ssNMR experiments were performed on a Bruker Avance III 600 spectrometer with resonance frequencies of 150.918 MHz. The spectra of components and co-amorphous mixtures were collected with a spinning rate of 12 kHz, and the relaxation delays (RDs) during the acquisitions equaled 5 and 300 s.

### 2.6 X-ray Powder Diffraction

XRPD data were collected for VAL, NIC, VAL/NIC co-amorphous dispersions, and VAL/NIC physical mixtures at room temperature with an Oxford Diffraction, Xcalibur3 CCD diffractometer using Cu Kα radiation. XRPD patterns were recorded for all samples after synthesis (1st day) and over 4th, 9th, and 11th months of storage under standardized conditions (25 °C, 60% RH)—for fully co-amorphized samples. XRPD experiments were performed using transmission geometry at room temperature with powdered samples sealed in capillary tubes and rotated about phi over 360° at 0.5°/S. CCD image data were processed using CrysAlisPRO software.^62^ The obtained data were analyzed using Fullprof software.^63^

### 2.7 Differential Scanning Calorimetry

DSC studies of VAL, NIC, their physical mixture, and co-amorphous mixtures were recorded using the Netzsch STA 409 C/CD instrument. The samples were placed in sealed, non-hermetic aluminum pans and scanned at a heating rate of 10.0 K/min under an argon atmosphere.

### 2.8 Scanning Electron Microscopy

A VEGA3 TESCAN scanning electron microscope was used to collect photo-micrographs of co-amorphous mixtures and their components. The samples were mounted on a metal stub with adhesive tape and observed without coating under high vacuum. For each sample, 200× magnification was applied.

### 2.9 Solution-State NMR Studies

The ^1^H NMR spectra were collected for co-amorphous formulations in order to investigate contents of ethanol in the obtained formulations. The ^1^H NMR spectra were measured on a Bruker AV III 500 spectrometer in CDCl_3_ with chemical shifts (δ) given in ppm relative to TMS as an internal standard.

### 2.10 Solubility Studies

The solubility of the co-amorphous dispersions obtained and free VAL and physical mixture of VAL and NIC was determined in distilled water and phosphate buffer (pH = 7.4) at 37 °C to simulate physiological conditions. The relevant sample was weighted in order to obtain supersaturated solution in water/phosphate buffer using a 20 mL test tube immersed in a thermostated water bath (37 °C) and equipped with a magnetic stirrer. The contents of the test tube were continuously stirred for 15 min; then, an additional amount of the relevant solvent (e.g., 0.1 mL) was added to the tube until the test substance completely dissolved, as determined with the macrocamera. The experiment was performed in triplicate and solubility values were expressed as a mean ± standard deviation. The solubility values for solvated co-amorphous solid dispersions and physical mixture were recalculated in reference to pure VAL, taking into account the content of VAL relative to NIC and EtOH in the obtained solvated solid dispersions.

## 3 Results and Discussion

### 3.1 Hydrogen-Bonding Interactions: FT-IR Spectra, DFT, QTAIM, and NCI Calculations

**Figure 2:**
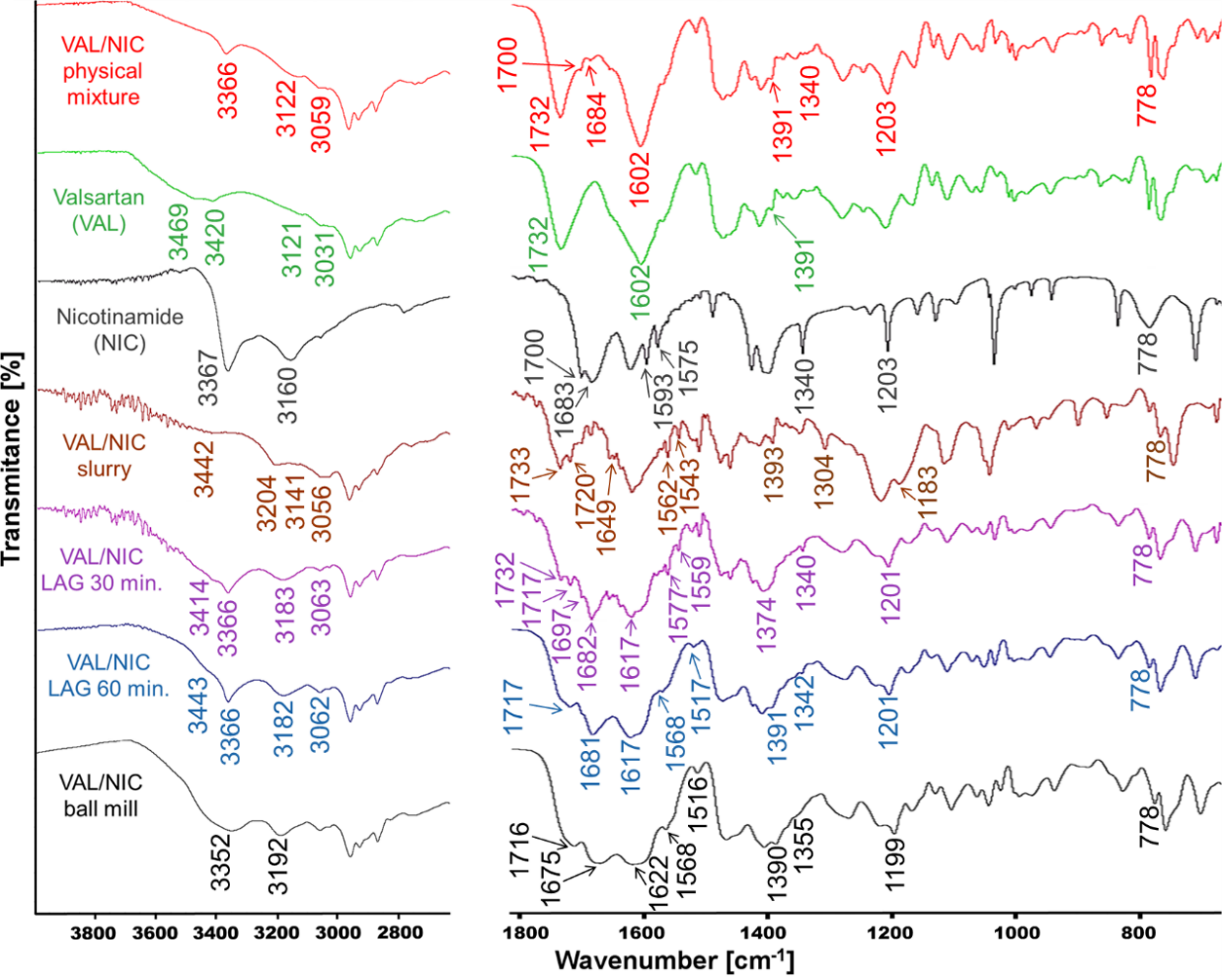
FT-IR spectra recorded for VAL, NIC, their physical mixture, and co-amorphous formulations.

The experimental FT-IR spectra of VAL, NIC, their physical mixtures, and co-amorphous formulations are presented in Figure 2. The FT-IR spectrum of VAL shows two characteristic sharp peaks at 1732 and 1602 cm^–1^, corresponding to the υ(C=O)^acid^ and υ(C=O)^amide^ vibrations, respectively. Broad, merged peaks with maxima at 3121 and 3420 cm^–1^ can be assigned to the υ(OH) and υ(NH) vibrations of VAL, respectively. The FT-IR spectrum of NIC indicates the presence of five characteristic absorption bands at 3367, 3160, 1683 cm^–1^ (with a shoulder at 1700 cm^–1^), 1593 , and 1575 cm^–1^ corresponding to the υ(NH_2_)^as^, υ(NH_2_)^s^, υ(C=O), and two δ(NH) vibrations, respectively. Two narrow sharp peaks at 1340 and 1203 cm^–1^ corresponding to the υ(C–N) and υ(C–NH_2_) vibrations are also observed along with the ρ_w_(NH_2_) broad peak at 778 cm^–1^. The characteristic vibrations and band shifting of the most important groups that can form hydrogen bonds are collected in Table 1. The spectrum of the VAL/NIC physical mixture reveals non-shifted peaks of single components, in particular carbonyl peaks of VAL (1732 and 1602 cm^–1^) and NIC (1684 cm^–1^ with a shoulder at 1700 cm^–1^), which has low intensity. A comparison of the spectra indicates that major bands of the starting compounds are shifted in the solid dispersions obtained. In case of the VAL/NIC ball mill sample, three carbonyl peaks are significantly shifted: υ(C=O)^amide^ and υ(C=O)^acid^ peaks of VAL and υ(C=O) peak of NIC are shifted from 1602, 1732^—^, and 1683 to 1622, 1716^—^, and 1675 cm^–1^ respectively. Two carbonyl peaks are also significantly shifted in the case of VAL/NIC slurry: the υ(C=O)^amide^ peak of VAL is shifted from 1602 to 1649 cm^–1^, whereas a υ(C=O) peak of NIC is moved from 1683 to 1720 cm^–1^.

**Table 1:** Characteristic, Experimental Values of FT-IR Vibrations for VAL, NIC, and Co-Amorphous Dispersions along with Relevant Shifts (Δ) in Relation to VAL and NIC

	observed frequencies [cm^–1^]					
assignment	NIC	VAL	VAL/NIC slurry	VAL/NIC LAG 30 min	VAL/NIC LAG 60 min	VAL/NIC ball mill
υ(NH)^tetrazole^		3420	3442 (Δ = +22)	3414 (Δ = −6)	3443 (Δ = +23)	3352 (Δ = −68)
υ(OH)		3121	3204 (Δ = +83)			
υ(C=O)^acid^		1732	1733 (Δ = +1)	1717 (Δ = −15)	1717 (Δ = −15)	1716 (Δ = −16)
υ(C=O)^amide^		1602	1649 (Δ = +47)	1605 (Δ = +3)	1605 (Δ = +3)	1622 (Δ = +20)
υ(C–O)		1391	1393 (Δ = +2)	1374 (Δ = −17)	1391 (Δ = 0)	1390 (Δ = −1)
υ(NH_2_)^as^	3367		3204 (Δ = −163)	3366 (Δ = −1)	3366 (Δ = −1)	3352 (Δ = −15)
(NH_2_)^s^	3160		3056 (Δ = −23)	3183 (Δ = +23)	3182 (Δ = +22)	3192 (Δ = +32)
υ(C=O)	1683^a^		1720 (Δ = +37)	1681 (Δ = −2)	1681 (Δ = −2)	1675 (Δ = −8)
δ(NH)	1593		1562 (Δ = −31)	1577 (Δ = −16)	1568 (Δ = −25)	1568 (Δ = −25)
δ(NH)	1575		1543 (Δ = −32)	1559 (Δ = −16)	1517 (Δ = −58)	1516 (Δ = −57)
υ(C–N)	1340		1304 (Δ = −36)	1340 (Δ = 0)	1342 (Δ = +2)	1355 (Δ = +15)
υ(C–NH_2_)	1203		1183 (Δ = −20)	1201 (Δ = −2)	1201 (Δ = −2)	1199 (Δ = −4)
ρ_w_(NH_2_)	778		778 (Δ = 0)	778 (Δ = 0)	778 (Δ = 0)	778 (Δ = 0)

a With a shoulder at 1700 cm–^1^ υ—stretching, δ—bending, ρ_w_—wagging, as—asymmetric vibrations, and s—symmetric vibrations.

Smaller bands shifts are observed in the case of the VAL/NIC LAG solid dispersions where the υ(C=O)^amide^ peak of VAL is slightly shifted from 1602 to 1605 cm^–1^, υ(C=O) peak of NIC is slightly moved from 1683 to 1681 cm^–1^, and υ(C=O) acid peak of VAL is shifted from 1732 to 1717 cm^–1^. It is worth noting that the υ(C=O)^acid^ peak of VAL is noticeably shifted only in the spectra of the VAL/NIC LAG and VAL/NIC ball mill solid dispersions, suggesting that the interactions between components in these formulations are different from those for the abovementioned VAL/NIC slurry dispersion. However, the additional peak at 1682 cm^–1^ corresponding to the unreacted NIC is additionally visible in the spectrum of the VAL/NIC LAG 30 min dispersion. Moreover, carbonyl bands are merged and broadened in the case of all formulations investigated, which suggests the formation of supramolecular heterosynthons that involve interactions of these C=O groups. The assignment and shift interpretation of the υ(OH) and υ(NH) vibrations of VAL is difficult due to peak broadening caused by the presence of ethanol OH groups (solvated dispersions). In the case of the VAL/NIC slurry dispersion, υ(NH_2_)^as^ of NIC is shifted from 3367 to 3204 cm^–1^ and the υ(NH_2_)^s^ vibration is shifted from 3160 to 3056 cm^–1^. Smaller shifts are observed in the case of the VAL/NIC ball mill solid dispersion, where υ(NH_2_)^as^ of NIC is shifted from 3367 to 3352 cm^–1^ and the υ(NH_2_)^s^ vibration is shifted from 3160 to 3192 cm^–1^. Such shifts in the υ(NH_2_) frequencies are not observed in the case of the VAL/NIC LAG dispersions, for which spectra reveal only υ(NH_2_)^s^ vibration shifted from 3160 to 3183 cm^–1^ (VAL/NIC LAG 30 min) and 3182 cm^–1^ (VAL/NIC LAG 60 min). Taking into account the presence of an additional peak at 1682 cm^–1^ with a shoulder at 1697 cm^–1^ in the FT-IR spectrum of the VAL/NIC LAG 30 min dispersion, which corresponds to the υ(C=O) peak of NIC and the υ(C=O)^acid^ peak of VAL at 1732 cm^–1^, it can be assumed that the VAL/NIC LAG 30 min dispersion is not fully co-amorphized and contains additionally free VAL and NIC.

**Figure 3:**
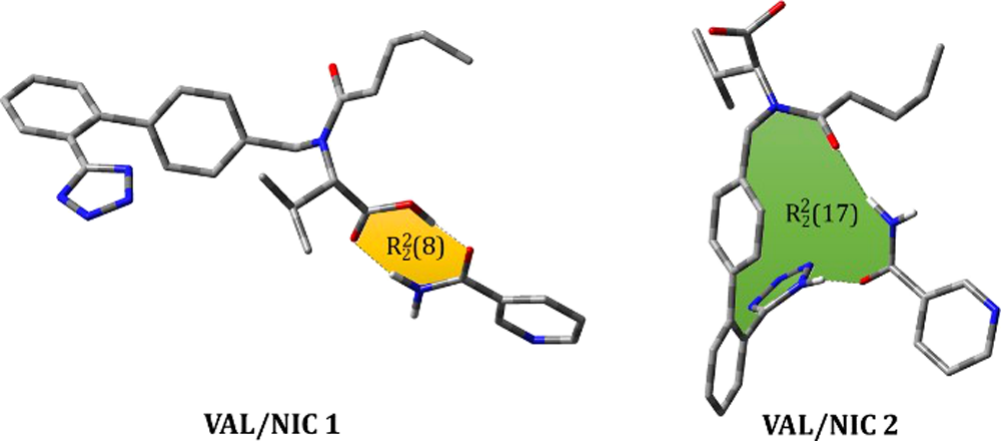
Optimized structures of the designed systems VAL/NIC1 (left) and VAL/NIC2 (right). Hydrogen atoms not involved in hydrogen bonds have not been shown for clarity.

**Figure 4:**
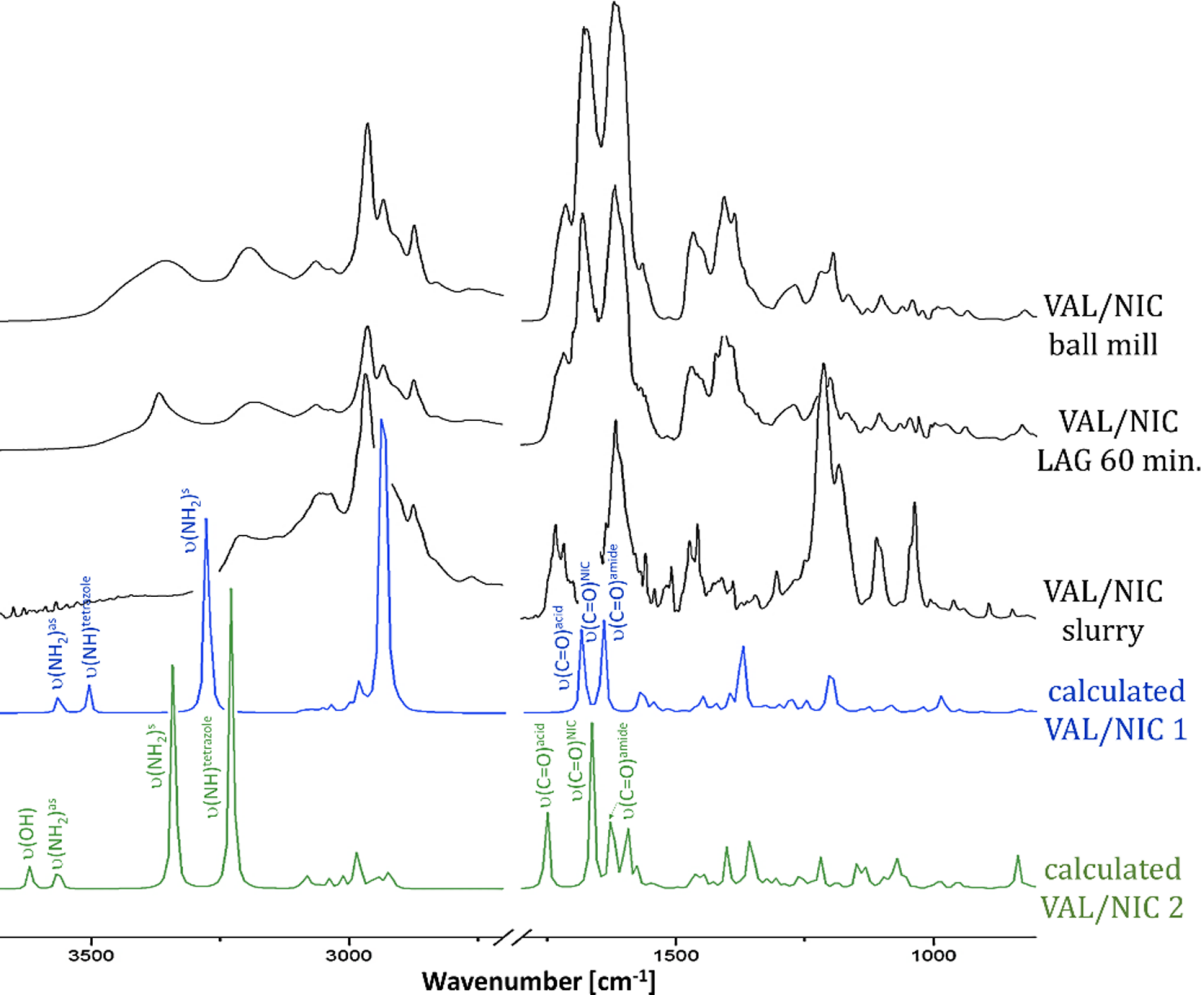
Comparison of experimental (VAL/NIC ball mill, LAG 60 min, and slurry) and theoretical (VAL/NIC1 and 2) FT-IR spectra.

In order to investigate what kind of intermolecular interactions could dominate in the synthesized co-amorphous solid dispersions, two systems differing in the existing supramolecular heterosynthons were designed and the theoretical IR spectra were calculated with the hybrid functional B3LYP combined with the 6-311++G(d,p) basis set (Figure 3). Then, the experimental IR frequencies of synthesized co-amorphous solid dispersions were compared with the calculated ones for VAL/NIC1 and VAL/NIC2 (Figure 4). In the designed VAL/NIC1 system, two hydrogen bonds, that is, N–H···O and O–H···O hydrogen bonds, connect VAL and NIC molecules into an acid–amide heterosynthon, which can be described by the *R*_2_2(8) graph-set notation,^64^ whereas in the VAL/NIC2 system, a combination of two hydrogen bonds (i.e., the first one between the tetrazolyl NH group of VAL and amide oxygen of NIC and the second between the amide oxygen of VAL and amide NH group of NIC) leads to the formation of a ring with the descriptor *R*_2_^2^(17).

A detailed comparison of experimental vibrations of co-amorphous solid dispersions with calculated frequencies for VAL/NIC1 and VAL/NIC2 is presented in the Supporting Information (Tables S7–S9).

The comparative analysis of the experimental and theoretical FT-IR spectra shows that the interactions occurring in the VAL/NIC slurry co-amorphous solid dispersion are mainly of the VAL/NIC1 type. A significant shift to a lower wavenumber of the υ(NH_2_)^s^ vibration is observed both in the experimental VAL/NIC slurry and calculated spectra. Moreover, the position and intensity of υ(C=O) vibrations in the VAL/NIC slurry spectrum are analogous to those calculated for VAL/NIC1 (two merged peaks for three υ(C=O) vibrations). Smaller shifts, due to υ(NH_2_)^s^ and υ(NH_2_)^as^ vibrations, are observed in the case of the calculated VAL/NIC2 system. The position and intensity of υ(C=O) vibrations in VAL/NIC2 are different from in the case of VAL/NIC1. This υ(C=O) vibration pattern, where three separate υ(C=O) bands are observed, is analogous to VAL/NIC LAG 60 min and VAL/NIC ball mill spectra; however, due to the overlapping of υ(C=O) vibrations, the presence of coexisting VAL/NIC1-type interactions in these samples may be suggested.

**Figure 5:**
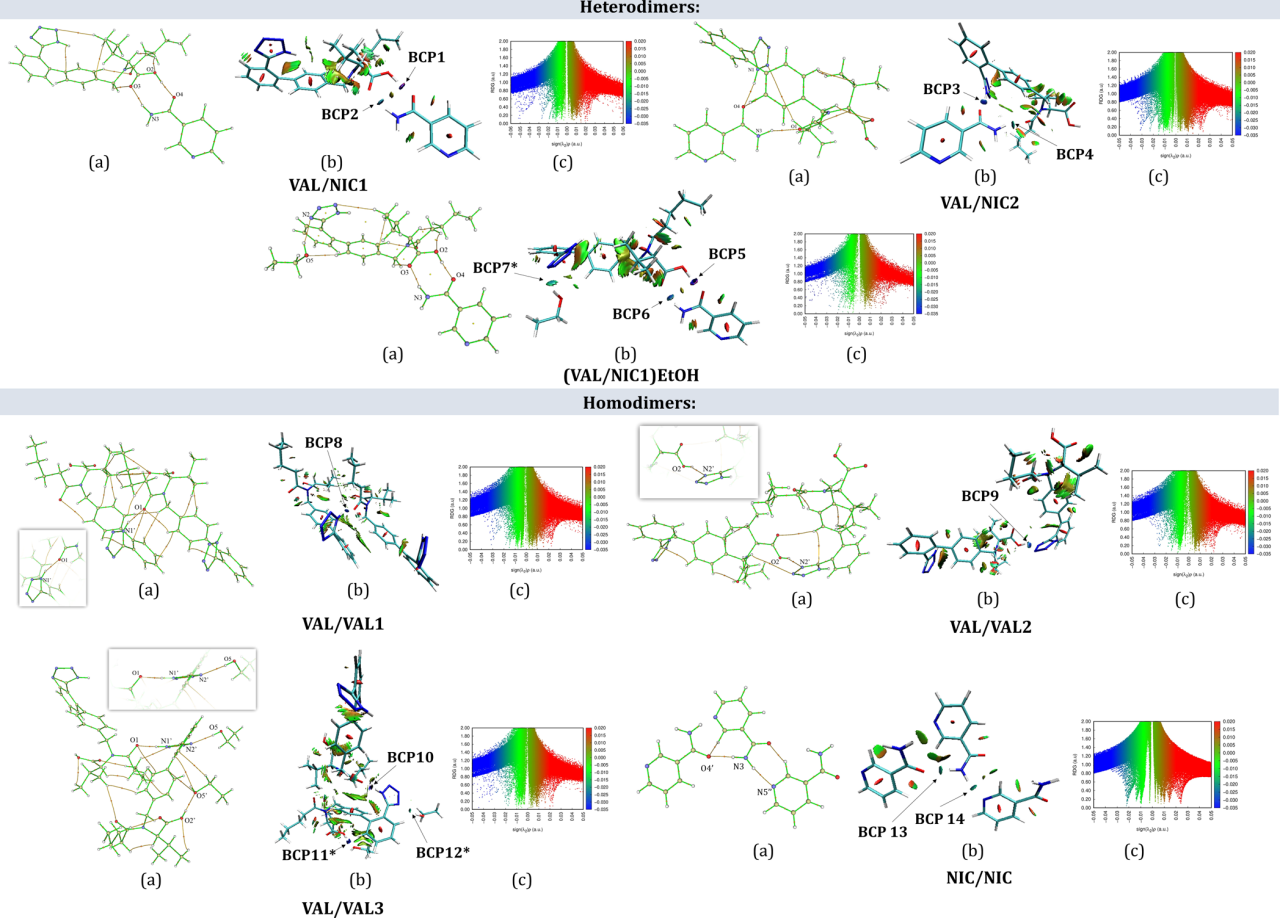
Molecular graphs with the BCPs (3, −1) as orange dots (a), 3D-NCI plot with color-filled RDG isosurfaces (b), and 2D-NCI scatter plots of the RDG vs sign (λ_2_)·ρ (c) for designed heterodimers [VAL/NIC1, VAL/NIC2, and (VAL/NIC1)EtOH], homodimers (VAL/VAL1-3) and homotrimers (NIC/NIC).

The results of QTAIM analysis, performed for two VAL/NIC heterodimers at the ground state in the gas phase, showed the BCPs expected for the interactions between the VAL and NIC molecules (Figure 5a). Further analysis of the topological parameters listed in Table 2 indicates that the N3–H3a···O3 interaction (BCP2 in VAL/NIC1) and N1–H1···O4 and N3–H3a···O3 interactions (BCP3 and BCP4, respectively, in VAL/NIC2) are characterized by small values of electron density and its Laplacian (0.023 < ρ_BCP_ < 0.032, 0.094 < ∇^2^ρ_BCP_ < 0.123) and fulfill the first three KP criteria for the existence of a hydrogen bond. Taking into account the fact that the fourth criterion (i.e., Δ*r*_H_ + Δ*r*_A_ > 0 and Δ*r*_H_ – Δ*r*_A_ > 0) is also met, all these interactions may be regarded as the hydrogen bonds. The positive values of ∇^2^ρ_BCP_ and *H*_BCP_ values indicate that accordingly,^65^ these three interactions may be classified as weak hydrogen bonds of electrostatic nature. The fourth KP criterion is also fulfilled for the O2–H2···O4 interaction (BCP1) in the VAL/NIC1 heterodimer. The BCP for this interaction is characterized by the highest positive values of charge density and its Laplacian (ρ_BCP_ = 0.0514 a.u, ∇^2^ρ_BCP_ = 0.1413 a.u), which both lie above the upper limits of these properties proposed by KP (i.e., ρ_BCP_ = 0.034 a.u, ∇^2^ρ_BCP_ = 0.139 a.u).^59^ The positive ∇^2^ρ_BCP_ value and negative *H*_BCP_ value allow us to classify this interaction as a medium H-bond^65^ with a partially covalent character. The medium strength of the O2–H2···O4 hydrogen bond is additionally confirmed by the value of hydrogen bond energy *E*_bin_ = −15.68 kcal/mol. The isosurface shape and color indicate that it is an interaction of type I, that is, strong stabilizing interaction.^66^

**Table 2:** Geometrical and Topological Parameters Corresponding to Conventional H-Bonds Involved in Intermolecular Interactions in Homo-/Heterodimers and Homotrimers

System	D–H···A	BCP no.	H···A[Å]	D···A[Å]	D–H···A[deg]	ρ_BCP_	∇^2^ρ_BCP_	*H*(*r*_BCP_)	*E*_bin_ [kcal/mol]
VAL/NIC1	O2–H2···O4	1	1.638	2.642	175.234	0.0514	0.1413	–0.0073	–15.68
	N3–H3a···O3	2	1.909	2.911	165.671	0.0271	0.0967	0.0019	–6.40
VAL/NIC2	N1–H1···O4	3	1.794	2.809	168.946	0.0316	0.1229	0.0017	–8.56
	N3–H3a···O1	4	1.936	2.915	160.112	0.0231	0.0938	0.0030	–5.45
(VAL/NIC1)EtOH	O2–H2···O4	5	1.642	2.646	175.332	0.0507	0.1408	–0.0069	–15.36
	N3–H3a···O3	6	1.904	2.906	165.816	0.0274	0.0976	0.0018	–6.49
	O5–H5 N2	7^a^	2.100	2.986	151.243	0.0195	0.0656	0.0023	–3.72
VAL/VAL1	N1′–H1′···O1	8	1.818	2.645	160.76	0.03176	0.1495	0.003757	–9.36
VAL/VAL2	O2–H2···N2′	9	1.985	2.712	147.37	0.02674	0.1021	0.002160	–6.65
VAL/VAL3	N1′–H1′···O1	10	1.713	2.670	176.77	0.04425	0.1497	–0.002667	–13.41
	O2′–H2′···O5′	11^a^	1.783	2.619	173.43	0.03490	0.1472	0.001451	–10.63
	O5–H5···N2′	12^a^	2.065	2.885	165.15	0.02171	0.0083	0.002595	–4.85
NIC/NIC	N3–H3a···O4′	13	2.076	3.075	169.31	0.02224	0.0699	0.001886	−4.30
	N3–H3b···N5′′	14	1.953	2.960	174.65	0.02152	0.0921	0.003456	−5.05

a Interaction with ethanol.

Although other hydrogen bonds between VAL and NIC molecules in the considered heterodimers are also the interactions of this type, the highest intensity of blue color of the isosurface and the lowest sign(λ_2_)·ρ value in the case of the O2–H2···O4 hydrogen bond in the VAL/NIC1 heterosynthon are further confirmation that it is the strongest interaction. In addition, the second hydrogen bond forming this heterosynthon, that is, the N3–H3a···O3 hydrogen bond, is slightly stronger than the N3–H3a···O1 hydrogen bond in the VAL/NIC2 heterodimer. Hence, it can be assumed that formation of the VAL/NIC1-type heterodimers is more preferred. This assumption is in agreement with the experimental FT-IR results, which suggest that VAL/NIC1-type interactions occur in the VAL/NIC slurry co-amorphous solid dispersion. It is not so clear in the case of the VAL/NIC LAG and ball mill solid dispersions where there are probably heterodimers of both types. It can be suspected that VAL/NIC1 and VAL/NIC2 heterodimers do not form a long-range order, remaining in a homogenous co-amorphous phase with a short-range order. This is due to the fact that the formation of non-covalent interactions connecting heterodimers with each other can be limited. For example, the VAL molecule in VAL/NIC1 adopts a conformation, such that the tetrazole ring is at an angle of 21.21° to the ring to which it is attached. In this way, the donor tetrazolyl N1–H1 group is isolated from any possible intermolecular hydrogen bonds which could lead to the formation of a co-crystal (Table 2, Figure 5). It is worth adding that the dihedral angle between the planes of abovementioned two rings of the VAL molecule in the crystal structure KIPLIG and KIPLEC is 57.51(1) and 48.37(5)°, respectively. The N1–H1 bond in VAL/NIC1 is directed, so that it participates in the formation of intramolecular contacts. They are revealed in the form of a relatively large green isosurface on the 3D NCI-plot (Figure 5b), which correspond to the sign (λ_2_)·ρ values of c.a. −0.005 a.u. in the 2D NCI-plot. As our solid dispersions are ethanol-solvated, we investigate the possible intermolecular interactions of ethanol with the VAL/NIC heterodimer by designing the (VAL/NIC1)EtOH system and doing the calculations in the framework of QTAIM and NCI methods—as in the case of earlier structures. The same, additional calculations were made for the VAL ethanol solvate crystal (CSD refcode KIPLEC) to compare the energy of interactions with ethanol. It can be seen that in the case of the VAL/NIC1 heterodimer, ethanol can interact only with VAL’s tetrazole ring because VAL’s carboxylic acid group is occupied by creating two strong hydrogen bonds (one with a partially covalent character) with NIC (binding energy −15.68 and −6.40 kcal/mol). The same O5–H5···N2′ intermolecular interaction, where the tetrazole ring acts as a hydrogen bond acceptor, occurs in the VAL ethanol solvate crystal (VAL/VAL3). The calculated energy of this interaction is −4.85 kcal/mol for the VAL/VAL3 and −3.72 kcal/mol for the (VAL/NIC)EtOH. Therefore, it has been shown that ethanol can form intermolecular interactions with the VAL/NIC system, which were further confirmed by the fact that this solvent could not be evaporated from the samples despite the use of high vacuum, which suggests strong binding of the solvent. It is worth mentioning that the previously described dihedral angle regarding the tetrazole ring in (VAL/NIC1)EtOH is 41.50° (almost two times higher than in VAL/NIC1 without ethanol); therefore, this interaction caused important conformational changes in the VAL molecule.

In order to compare the energies of intermolecular hydrogen bonds in VAL/NIC heterodimers with those identified in the crystal structures of VAL and NIC,^67^ analogous QTAIM and NCI calculations were conducted for molecular pairs cut out from these crystals. VAL crystallizes in an orthorhombic space group *P*2_1_2_1_2_1_ in two possible polymorphic forms E and F, which due to the presence of an ethanol molecule in one of them differ in both the molecular conformation and the packing motifs.^15^ In the crystal structure of form E, the VAL molecules are connected by the O–H_(carboxylic)_···N_(tetrazole)_ (VAL/VAL1) and N–H_(tetrazole)_···O=C_(carbonyl)_ (VAL/VAL2) hydrogen bonds into the two-dimensional framework. In the crystal structure of the solvated form F (VAL/VAL3), there are three different hydrogen bonds: one—between N–H atoms of the tetrazole ring and carbonyl O atom, linking the VAL molecules (i.e., N–H_(tetrazole)_···O_(carbonyl)_) and two bonds connecting the VAL molecule with the ethanol molecule (i.e., O–H_(carboxylic)_···O_(ethanol)_ and O–H_(ethanol)_···N_(tetrazole)_). The obtained results show that the energy of intermolecular hydrogen bonds in the NIC/NIC trimer is significantly lower than the energy between VAL and NIC in the designed heterodimers (Figure 5, Table 2). Based on the energy of intermolecular interactions, one can conclude that the VAL/NIC1 heterodimer forms stronger hydrogen bonds than VAL/VAL homodimers. Therefore, the abovementioned data further confirm the preference of VAL to interact with NIC and this way to form heterodimers instead of individual homodimers/trimers, especially in the VAL/NIC1 system, where the intermolecular bonds are the strongest of all systems studied.

Due to the fact that the solid dispersions were obtained in a solvated form, additional DFT, QTAIM, and NCI calculations were carried out for heterodimers optimized in ethanol. In general, the results obtained both in the gas phase and in ethanol were comparable. The heterodimers seemed to be more stable in the presence of ethanol due to a slightly lower total energy of the system (Tables S4a,b and S5a,b). It was noted that the strongest intermolecular hydrogen bonds (O2–H2···O4 in VAL/NIC1 and N1–H1···O4 in VAL/NIC2) were further strengthened in the presence of ethanol. It is revealed by the highest binding energy of these hydrogen bonds (Table S10, SM) and elongation of the D(donor)–H length and shortening of the H···A(acceptor) and D···A lengths in the presence of ethanol (Table S11).

The interactions between solutes represented by the designed heterodimers and solvents (water and ethanol) were investigated by the PCM, first described in 1981 by Tomasi et al.^68−73^ Interactions with water are important to get the initial view of the expected solubility of the designed heterodimers. Since the obtained solid dispersions are ethanol-solvated, the solvation energies were also calculated in relation to this solvent. Calculated free energies of solvation, its components, and dipole moments for both heterodimers are presented in Table 3. In general, the greater the value of solvation free energy is, the higher the solubility is expected.

**Table 3:** Free Energies of Solvation, Its Components, and Dipole Moments Calculated with the PCM Model at the B3LYP/6-311++G(d,p) Level of Theory

							dipole moment [*D*]	
heterodimer	Δ*G*_solv_ [kcal/mol]	*E*_cavitation_ [kcal/mol]	*E*_dispersion_ [kcal/mol]	*E*_non-electrost__._ [kcal/mol]	*E*_polar_ [kcal/mol]	*E*_electrost_ [kcal/mol]	in gas phase	in ethanol
Solvent = Ethanol								
VAL/NIC1	4.73	62.30	–42.06	22.75	3.17	–18.02	4.63	4.78
VAL/NIC2	6.96	62.84	–39.06	26.11	2.99	–19.15	8.11	10.06
(VAL/NIC1)EtOH	10.56	69.96	–45.14	27.52	2.81	–16.96	2.37	2.25

							dipole moment [*D*]	
heterodimer	Δ*G*_solv_ [kcal/mol]	*E*_cavitation_ [kcal/mol]	*E*_dispersion_ [kcal/mol]	*E*_non-electrost__._ [kcal/mol]	*E*_polar_ [kcal/mol]	*E*_electrost_ [kcal/mol]_._	in gas phase	in water
Solvent = Water								
VAL/NIC1	19.36	83.24	–47.95	38.32	3.56	–18.96	4.63	4.77
VAL/NIC2	22.11	83.97	–44.54	42.33	3.34	–20.11	8.11	10.17
(VAL/NIC1)EtOH	27.28	93.40	–51.50	45.16	3.18	–17.88	2.37	2.27

As the results of these studies, the VAL/NIC2 heterodimer was slightly more soluble both in water and ethanol. Therefore, these heterodimers may occur in solid dispersions, which contain more ethanol and exhibit higher water solubility. The contributions of electrostatic and non-electrostatic interactions to the solvation free energy are comparable in two heterodimers; however, a slight predominance of non-electrostatic interactions was observed in the case of VAL/NIC2. It has been reported that non-electrostatic interactions, such as van der Waals interactions, play a major role in solubilizing the pharmaceuticals,^74^ which further confirms the assumption that the VAL/NIC2 heterodimer may have a greater solubility than the VAL/NIC1. Moreover, in the case of the VAL/NIC2 heterodimer, the difference in dipole moment values in the gas phase and ethanol has been observed. The dipole moments calculated in the solvent (both in water and ethanol) are higher than those calculated in the gas phase—therefore, this type of heterodimers should be more polar and more soluble in water (especially in the presence of ethanol). This difference in dipole moments is not observed in the case of the VAL/NIC1 heterodimer and (VAL/NIC1)EtOH. In case of the (VAL/NIC1)EtOH system, values of free energy of solvation and its cavitation component are much higher than the same values for VAL/NIC1. Moreover, it can be seen that the presence of ethanol increases the role of non-electrostatic interactions, which are an important factor affecting solubility. Taking into account these results, it can be suspected that VAL/NIC2-type heterodimers may dominate in the VAL/NIC ball mill co-amorphous solid dispersion, causing higher solubility of this sample, what has, in fact, been observed during the solubility tests (*vide infra*).

### 3.2 Solid-State NMR

**Figure 6:**
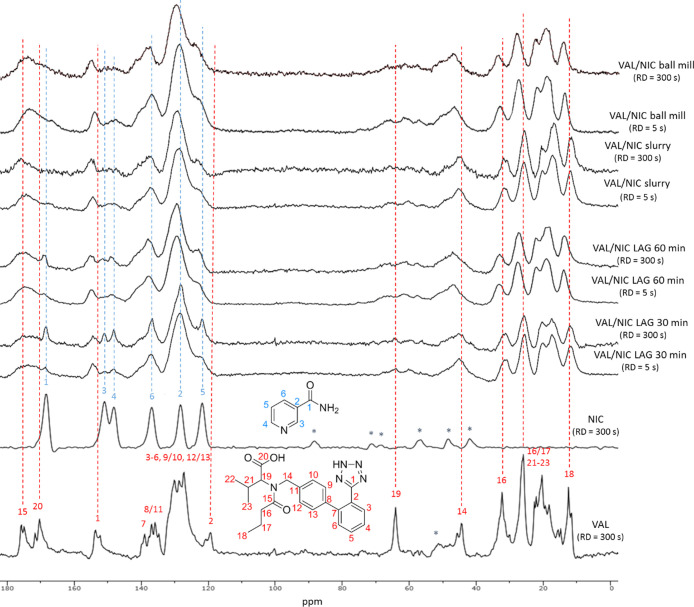
^13^C CPMAS NMR spectra of VAL, NIC, and co-amorphous formulations (RD = 5 and 300 s). Asterisks denote spinning bands.

There is a lack of data regarding the ssNMR studies of co-amorphous mixtures of VAL. Although NIC is a commonly used stabilizer of amorphous drugs, the number of ssNMR studies is mainly limited to its co-crystals.^75−78^ It is worth noting that to obtain a high-quality ^13^C CP-MAS NMR spectrum of NIC, the RDs during the acquisition should be extended to 300 s^75^ For this purpose, ^13^C CP-MAS NMR spectra of components and co-amorphous formulations were recorded with a spinning rate of 12 kHz and recycle delays of both 5 and 300 s (Figure 6). The spectra recorded for VAL with RDs equal to 5 and 300 s were identical (Figure S5), while it was impossible to record the ssNMR spectrum of NIC with the short RD = 5 s (due to very long relaxation times for this compound). However, the ^13^C signals of the latter were visible in the spectra of co-amorphous mixtures and could be recorded at RD = 5 s. This may be due to the fact that relaxation times for new formulations were shortened in comparison for the starting components, which confirms formation of a new phase. Similar results have been reported for the NIC co-crystals.^75−77^ Moreover, the shifted, broadened, and merged carbonyl peaks in co-amorphous formulation spectra (δ = 160–180 ppm) indicate the formation of non-covalent bonds that involve the C=O groups. It is especially evident in the case of fully co-amorphous VAL/NIC slurry and VAL/NIC ball mill samples. In the case of the VAL/NIC LAG 30 min sample, the ssNMR spectrum, recorded with RD = 300 s, revealed strong peaks, which correspond to the signals of NIC, so it can be assumed that this sample contains additionally unreacted NIC (which was also confirmed by other analyses).

### 3.3 Differential Scanning Calorimetry

**Figure 7:**
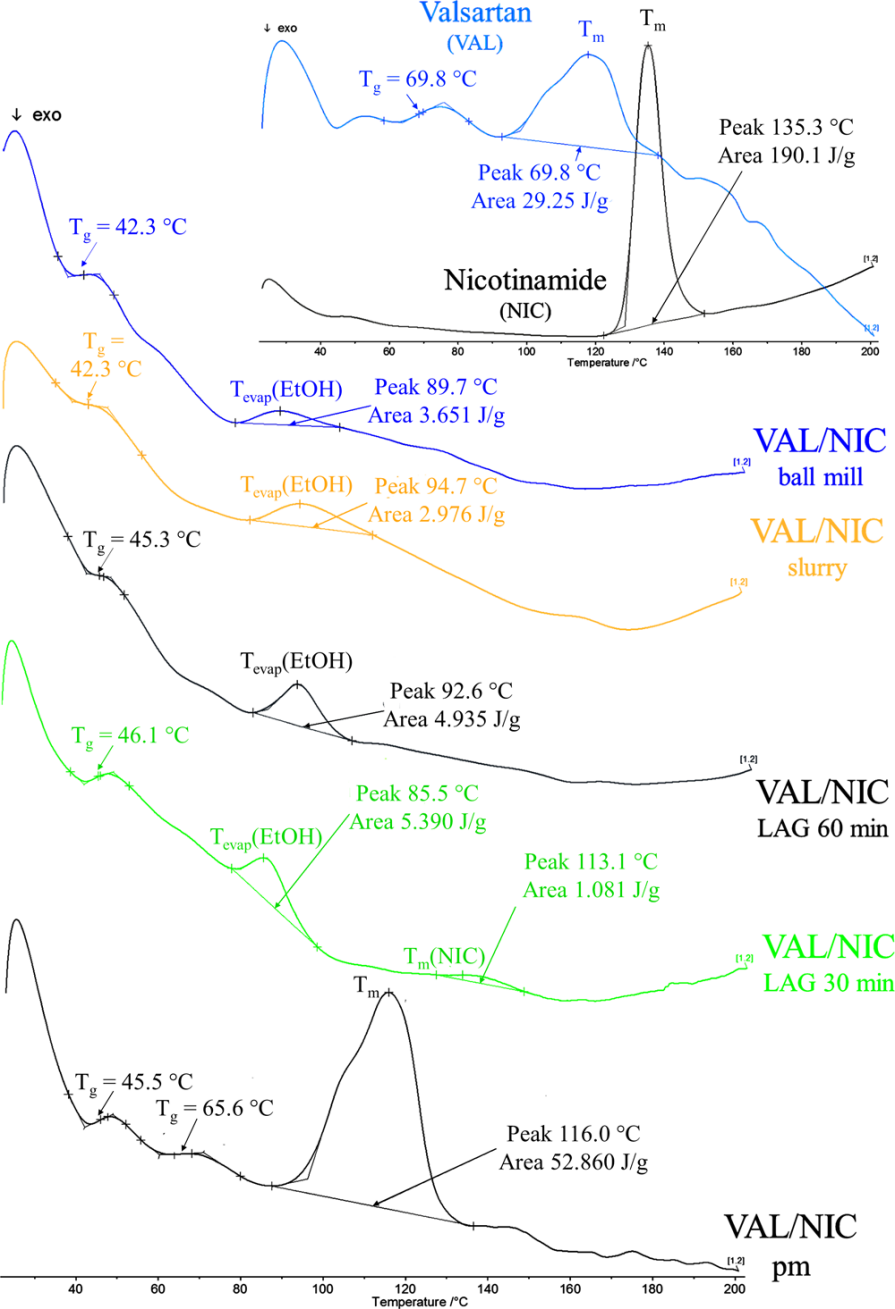
DSC thermograms of co-amorphous mixtures (VAL/NIC LAGs, VAL/NIC slurry, and VAL/NIC ball mill), VAL/NIC physical mixture (pm) and pure components (VAL and NIC).

The DSC thermograms of the VAL/NIC co-amorphous mixtures and the VAL/NIC physical mixture along with the insert of DSC thermograms of components (VAL and NIC) are presented in Figure 7. The NIC spectrum showed a single melting endothermic peak at 135.3 °C, whereas the VAL revealed the melting endothermic peak (*T*_m_) with the maxima at 117.9 °C (Figure 7, insert). The physical mixture of VAL and NIC showed a single broad endothermic peak at 116.0 °C and two glass transition events (*T*_g_) corresponding to NIC (45.5 °C) and VAL (65.6 °C).

What is important is that the peaks corresponding to the VAL/NIC physical mixture and pure components disappeared in thermograms of the co-amorphous dispersions obtained. However, new melting points do not appear, which indicates that the formed samples are amorphous. The co-amorphous formulations studied exhibited two endothermic events: first, glass transition at 44.0 °C (VAL/NIC slurry), 46.1 °C (VAL/NIC LAG 30 min), 45.3 °C (VAL/NIC LAG 60 min), and 42.3 °C (VAL/NIC ball mill) and second, vaporization of ethanol (*T*_evap_(EtOH)) at 94.7 °C (VAL/NIC slurry), 85.5 °C (VAL/NIC LAG 30 min), 92.6 °C (VAL/NIC LAG 60 min), and 89.7 °C (VAL/NIC ball mill).^79^ All studied co-amorphous mixtures exhibited a single glass transition event, which indicated that they are homogeneous in contrast to their physical mixture, which exhibited two separate glass transition events.^19^ In case of the VAL/NIC LAG 30 min, a small peak with a maximum at 133.1 °C corresponding to the melting peak of NIC (135.3 °C) is visible, which again confirms the presence of additional unreacted NIC in a sample. This analysis clearly indicates that elongation of the grinding time from 30 min (VAL/NIC LAG 30 min) to 60 min (VAL/NIC LAG 60 min) increases the conversion of components to give co-amorphous dispersion.

### 3.4 Solution-State NMR Studies

Because the vaporization peak from ethanol was found in DSC thermograms of all co-amorphous dispersions (Figure 7), it could be expected that the synthesized formulations were obtained in the form of ethanol solvates. In order to quantitatively estimate the amount of ethanol, the solution-state ^1^H NMR spectra were recorded (Figure S9). The ethanol content was sensitive to the degree of amorphization. The calculated amounts of ethanol in fully amorphized dispersions VAL/NIC slurry and VAL/NIC ball mill and in the nearly amorphized VAL/NIC LAG 60 min sample remained in the range of 4.43–6.79% (by moles), while in the less-amorphized VAL/NIC LAG 30 min sample, the ethanol content dropped to 1.47% (by moles).

### 3.5 X-ray Powder Diffraction

**Figure 8:**
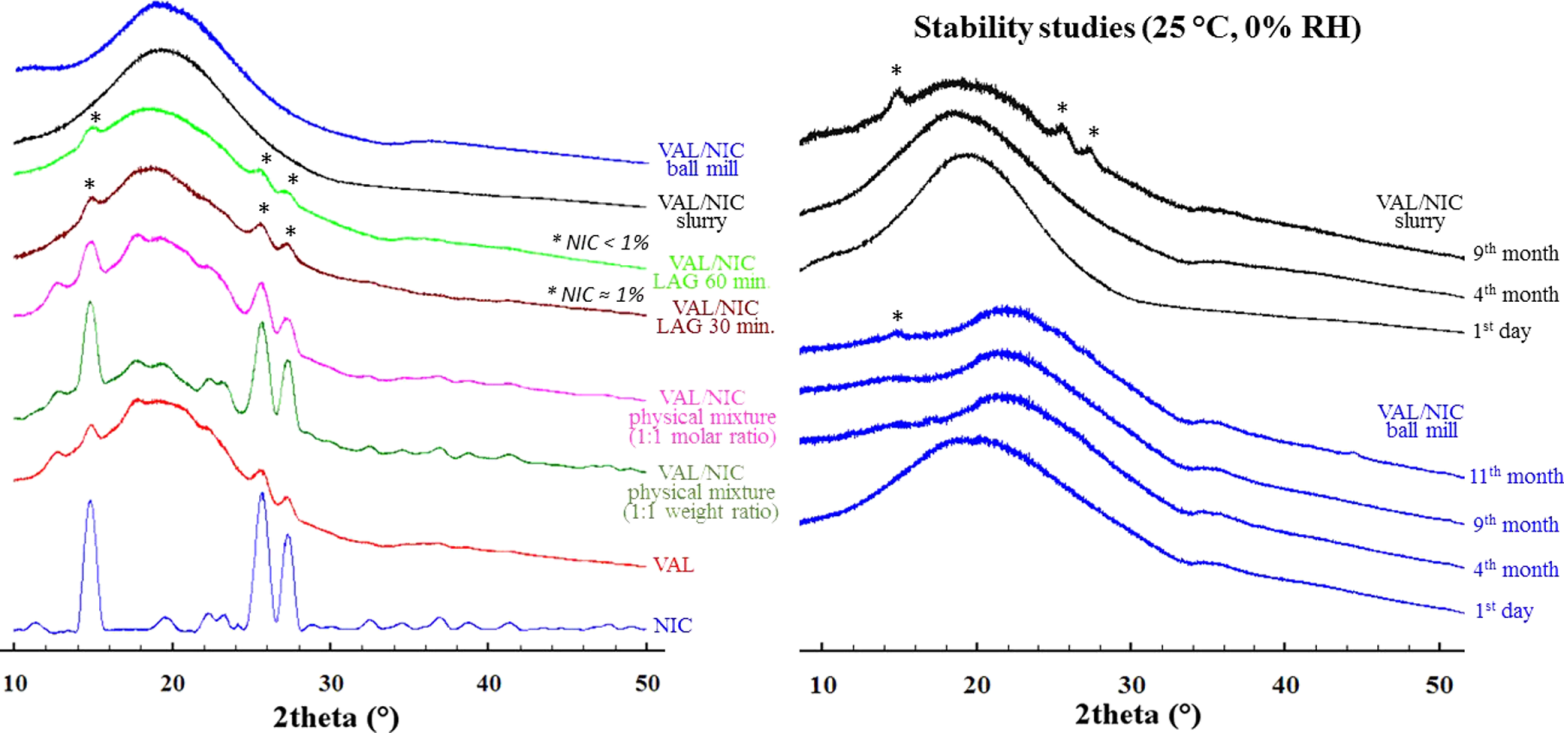
XRPD patterns of co-amorphous solid dispersions, their physical mixtures, and single components. Asterisks denote reflections corresponding to the unreacted crystalline NIC (its amount was calculated based on DSC experiments from the values of melting enthalpy).

The XRPD patterns of co-amorphous solid dispersions (VAL/NIC LAG 30 min, VAL/NIC LAG 60 min, VAL/NIC slurry, and VAL/NIC ball mill), their physical mixtures in a 1:1 molar and weight ratios, and their components (VAL and NIC) are presented in Figure 8. Detailed analysis of the XRPD pattern of VAL is given in the Supporting Information (Figure S7). The XRPD pattern of the NIC sample used in this study can be accurately matched to the simulated XRPD pattern, which was generated from the deposited X-ray data file at the Cambridge Structure Database (CSD—refcode NICOAM06) using Mercury software (Figure S8).

The XRPD analysis revealed that the pattern of the co-amorphous mixtures obtained is different from that for the VAL/NIC physical mixtures. Characteristic reflections of components (VAL and NIC) completely disappeared in the XRPD patterns of the VAL/NIC slurry and VAL/NIC ball mill. The appearance of an amorphous halo in the XRPD pattern of the solid dispersions demonstrated the amorphous nature of the obtained drug dispersions. It can be also concluded that the VAL/NIC slurry and VAL/NIC ball mill dispersions are different from the VAL/NIC LAG (30 and 60 min) dispersions, which exhibit additional three small reflections at 2θ: 14.88, 25.53, and 27.15°. These reflections correspond to the most intensive reflections in the XRPD pattern generated based on the crystal structure of NICOAM06. Thus, they can be related to incomplete amorphization of NIC in VAL/NIC LAG solid dispersions. Moreover, it can be estimated that the amount of non-amorphized NIC is greater in the VAL/NIC LAG 30 min than in the VAL/NIC LAG 60 min solid dispersion, which suggests that longer grinding time results in a more amorphous product; however, the grinding time of 60 min is still too short to fully co-amorphize this system with this technique. All of the abovementioned data prove that as a result of intermolecular interactions, the synthesized VAL solid dispersions formed new phases. The stability of these co-amorphous products, and thus their resistance to recrystallization, was tested under 25 °C, 0% RH conditions (Figure 8). The fact of appearance of a crystalline phase of any of the components of the co-amorphous product (VAL or NIC) indicates a loss of stability.^200^ Of these two components, NIC has much greater tendency to crystallization than VAL. Therefore, it could be expected that during the loss of amorphous stability, the reflections from NIC would first reveal on the diffraction pattern of the product. In fact, the XRPD method showed the presence of at least one reflection derived from the NIC crystalline phase present in the recrystallized products. In case of the VAL/NIC ball mill sample, first small reflection has been observed after 11 months of storage what confirms the excellent durability of this co-amorphous sample. Recrystallization of the VAL/NIC slurry sample was observed after 9 months of storage.

### 3.6 Scanning Electron Microscopy

**Figure 9:**
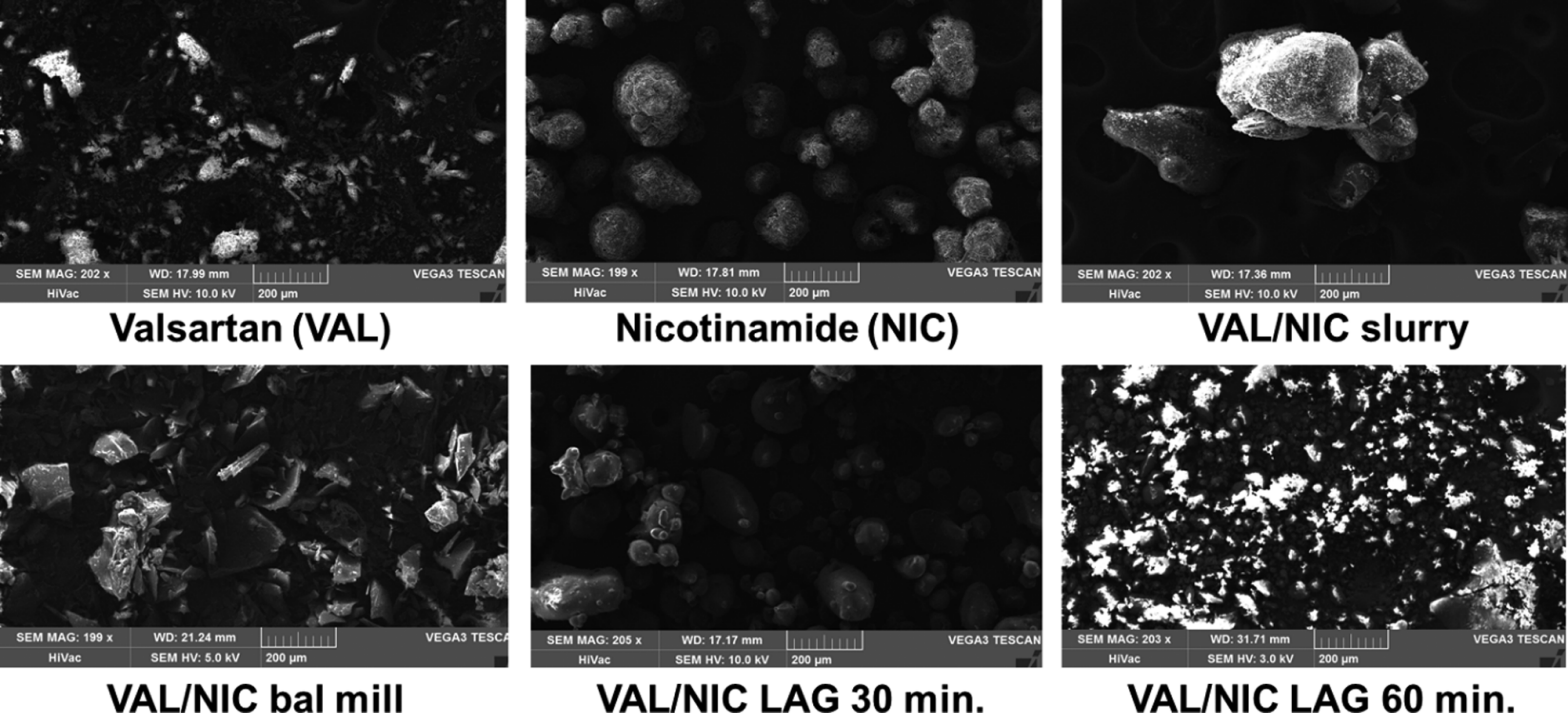
SEM images of VAL, NIC, and co-amorphous mixtures at ×200 magnification.

The SEM image overview of pure VAL, NIC, and co-amorphous formulations is presented in Figure 9. The SEM results reveal that VAL occurs in the form of irregular particles (up to 200 μm) with a rough surface and NIC is in the form of spherical rough grains (50–100 μm). The obtained particles of co-amorphous mixtures exhibited irregular shapes, different from those recorded for the pure VAL and NIC. The size of the VAL/NIC LAG 60 min particles is <50 μm, and the size of the VAL/NIC LAG 30 min particles remains in the range of 50–200 μm, whereas the VAL/NIC slurry particles are larger (>200 μm). The VAL/NIC ball mill particles remain in the range of 50–200 μm and form thin solid plates.

### 3.7 Solubility Studies

The main goal of the solid dispersion synthesis is improving solubility of poorly soluble drugs (in this case—VAL). The solubility studies are performed in suitable medium simulating physiological conditions. In this paper, the solubility of the free VAL and four co-amorphous mixtures was investigated in the phosphate buffer at the physiological pH 7.4 and in distilled water at 37 °C (Table 4). The results showed that the VAL/NIC LAG 30 min and VAL/NIC LAG 60 min solid dispersions exhibited similar solubility both in the phosphate buffer and water. These co-amorphous mixtures caused a 3.5-fold increase in solubility of VAL in water and only a 1.2-fold increase in solubility in the phosphate buffer. On the other hand, co-amorphous VAL/NIC slurry solid dispersion slightly increased solubility of VAL in the phosphate buffer, whereas a significant 14-fold increase in solubility was observed in water. An enhanced 24-fold higher water solubility than free VAL showed in the VAL/NIC ball mill solid dispersion. Therefore, taking into account the difference in the solubility of the VAL/NIC LAG (30 and 60 min) dispersions and the VAL/NIC slurry and VAL/NIC ball mill dispersions, it can be assumed that these co-amorphous formulations involve different intermolecular interactions with different morphologies of the powders, which was confirmed here by the relevant analyses and calculations. Interestingly, the solubility of the VAL/NIC ball mill sample with first signs of recrystallization remained the same in phosphate buffer, whereas its solubility in water decreased. This further confirms that co-amorphization significantly improves the solubility in water and has lesser effect on the solubility in the buffer. This also supports the thesis that lower solubility of VAL/NIC LAG samples in water is a consequence of their not fully amorphous nature.

**Table 4:** Solubilities of Free VAL,VAL Co-Amorphous Products and VAL/NIC Physical Mixture in the Phosphate Buffer and Water (37 °C) (*n* = 3)^a^

	solubility [mg/mL]	
drug/soliddispersion	phosphate buffer	water
VAL	8.53 ± 0.51,6.5^80^	0.16 ± 0.05,0.192^80^
VAL/NIC physical mixture	12.29 (9.59) ± 0.50	0.49 (0.38) ± 0.01
VAL/NIC LAG 30 min	13.25 (10.34) ± 0.36	0.70 (0.55) ± 0.02
VAL/NIC LAG 60 min	13.03 (10.09) ± 0.28	0.74 (0.57) ± 0.16
VAL/NIC slurry	10.83 (8.40) ± 0.20	2.93 (2.27) ± 0.06
VAL/NIC ball mill	10.55 (8.12) ± 0.26	5.01 (3.87) ± 0.76
VAL/NIC ball mill^b^	10.55 (8.12) ± 0.67	3.06 (2.36) ± 0.20

a The values recalculated in reference to pure VAL are shown in brackets.

b Sample after 11 months of storage at 0% RH and 25 °C.

## 4 Conclusions

Four solvated solid dispersions of the hypertension drug VAL with enhanced solubility in water and the phosphate buffer were synthesized. NIC, as a nutraceutical, was used as a suitable co-former to give dually acting pharmaceutical solid dispersions. The VAL/NIC slurry and VAL/NIC ball mill dispersions revealed full amorphization and the best solubility. These compositions could be used to treat two seemingly unrelated diseases, such as hypertension and the COVID-19 disease.^81^ This is due to the fact that SARS-CoV-2 enters host cells by utilizing angiotensin-converting enzyme 2 (ACE2), an enzyme which is also a target for treatment of hypertension. The described co-amorphous solids of VAL may contribute to establishing a new perspective in the hypertension treatment, in which in addition to taking anti-hypertensive drugs (VAL), patients will also support their anti-viral immune response (NIC). Moreover, the anxiety-reducing potential of NIC is particularly important in COVID-19 pandemic associated with an increased risk of mental disorders, which can potentially compromise blood pressure control.^82^ It has been demonstrated that applying FT-IR analysis assisted by DFT, QTAIM, and NCI calculations in the field of co-amorphous solid dispersions allows us to estimate what kind of intermolecular interactions occurs in the pharmaceutical amorphous, solid forms. Analysis of VAL/NIC systems *via* the FT-IR technique confirmed that strong hydrogen bonds between the two components play a significant role in their stability, while QTAIM and NCI calculations provided a clear insight into the stability mechanism at the molecular level. The described approach revealed the undeniable importance of hydrogen bonds between VAL and NIC in the formation of co-amorphous compositions. These interactions cause important changes in the arrangement of the VAL tetrazole ring, which results in the reduced participation of the tetrazolyl N–H donor and thus reduced co-crystallization. Therefore, the proposed methodology can be used as a general approach to the design of the desired co-amorphous solid materials including drugs. Based on the analyses performed, it can be concluded that co-amorphous solid dispersions obtained by the solution- and solid-state approaches involved different kinds of intermolecular interactions, which also resulted in a slightly different solubility of the dispersions studied. Moreover, it can be assumed that the solution (slurry) and ball mill techniques provide more homogenous co-amorphous solid dispersions than the liquid-assisted grinding.

## Supporting Information Available

Chemical analyses (DSC curve, FT-IR, NMR, HRMS spectra, and XRPD patterns) of components. DFT calculations: cartesian coordinates and total energies for the optimized structures (DFT), theoretical FT-IR spectra, topological and geometrical parameters corresponding to H-bonds along with RDG isosurfaces, and scatter graphs (QTAIM) (PDF)
