# Supplementary material for: Role of Hydrogen Bonds in Formation of Co-amorphous Valsartan/Nicotinamide Compositions of High Solubility and Durability with Anti-hypertension and Anti-COVID-19 Potential
Source: Mol Pharm. 2021 Apr 1;18(5):1970–84. doi: 10.1021/acs.molpharmaceut.0c01096 (PMC8029446; doi:10.1021/acs.molpharmaceut.0c01096)
Supplement: Supplementary file 1 — mp0c01096_si_001.pdf [file mp0c01096_si_001.pdf]

# The role of hydrogen bonds in formation of co-amorphous valsartan/nicotinamide compositions of high solubility and durability with anti-hypertension and anti-COVID-19 potential

Marika Turek [a*], Ewa Różycka-Sokołowska [a*], Marek Koprowski [b], Bernard Marciniak [a], Piotr Bałczewski [a,b*]

[a] Institute of Chemistry, Faculty of Science and Technology, Jan Długosz University in Częstochowa, Armii Krajowej 13/15, Częstochowa, 42-201, Poland;

[b] Division of Organic Chemistry, Centre of Molecular and Macromolecular Studies, Polish Academy of Sciences, Sienkiewicza 112, Łódź, 90-363, Poland.

* Piotr Bałczewski, e-mail: pbalczew@cbmm.lodz.pl
* Marika Turek, e-mail: marika.turek@ajd.czest.pl;
* Ewa Różycka-Sokołowska, e-mail: e.sokolowska@ajd.czest.pl

## SUPPLEMENTARY MATERIAL

**1. Valsartan (VAL)** ........ **3**

1.1. Extraction of VAL from tablets ........ 3

1.2. DSC analysis ........ 3

**Fig. S1.** DSC curve of VAL extracted from tablets. ........ 3

1.3. FT-IR spectroscopy ........ 4

**Fig. S2.** The IR spectrum of VAL extracted from tablets. ........ 4

**Table S1.** FT-IR characteristic vibrations of isolated VAL compared with published data [4,5] ........ 4

1.4. NMR analysis ........ 4

**Fig. S3a.** $^1$H NMR spectra of VAL extracted from tablets (δ 8-3 ppm). ........ 5

**Fig. S3b.** $^1$H NMR spectra of VAL extracted from tablets (δ 3-0 ppm). ........ 5

**Fig. S4.** $^{13}$C NMR spectra of VAL extracted from tablets. ........ 6

1.5. ssNMR analysis ........ 6

**Fig. S5.** $^{13}$C ssNMR spectra of VAL extracted from tablets and purchased (referenced). Asterisks denote spinning sidebands. ........ 7

1.6. HRMS analysis ........ 8

**Fig. S6.** MS spectrum of VAL extracted from tablets. ........ 8

1.7. XRPD ........ 8

**Fig. S7.** XRPD pattern of (a) VAL used in this study; (b) VAL purely amorphous; (c) VAL polymorphic form VII. 9

**2. Nicotinamide (NIC)** ........ **9**

2.1. XRPD ........ 9

**Fig. S8.** XRPD pattern of NIC used in this study along with designation of major reflexes. ........ 9

**3. Solid dispersions (VAL/NIC)** ........ **10**

3.1. Solution-state NMR studies ........ 10

**Fig. S9.** $^1$H NMR spectra for (a) VAL/NIC slurry, (b) VAL/NIC LAG 30 min, (c) VAL/NIC LAG 60 min, (d) VAL/NIC ball mill along with calculations regarding the content of EtOH. ........ 11

**4. Preliminary semi-empirical calculations** ........ **12**

**Fig. S10.** Chemical structures of 5 possible VAL/NIC heterodimers optimized by using AM1 method along with calculated total energy (Hartree). ........ 12

**5. DFT calculations** ........ **13**

5.1. Cartesian coordinates and total energies for the optimized structures ........ 13

**Table S2.** Atom coordinates (Å), total energy (Hartree) and the number of imaginary vibrational frequencies for the geometry of **VAL** optimized at the B3LYP/6-311++(d,p) level in the gas phase using Gaussian 09. ........ 13

**Table S3.** Atom coordinates (Å), total energy (Hartree) and the number of imaginary vibrational frequencies for the geometry of **NIC** optimized at the B3LYP/6-311++(d,p) level in the gas phase using Gaussian 09. ........ 14

**Table S4a.** Atom coordinates (Å), total energy (Hartree) and the number of imaginary vibrational frequencies for the geometry of **VAL/NIC1** optimized at the B3LYP/6-311++(d,p) level in the **gas phase** using Gaussian 09. ........ 14

**Table S4b.** Atom coordinates (Å), total energy (Hartree) and the number of imaginary vibrational frequencies for the geometry of **VAL/NIC1** optimized at the B3LYP/6-311++(d,p) level in the **ethanol** using Gaussian 09. 16

**Table S5a.** Atom coordinates (Å), total energy (Hartree) and the number of imaginary vibrational frequencies for the geometry of **VAL/NIC2** optimized at the B3LYP/6-311++(d,p) level in the **gas phase** using Gaussian 09. ........17

**Table S5b.** Atom coordinates (Å), total energy (Hartree) and the number of imaginary vibrational frequencies for the geometry of **VAL/NIC2** optimized at the B3LYP/6-311++(d,p) level in the **ethanol** using Gaussian 09. 19

5.2. Vibrational spectra ........ 21

**Table S6.** Comparison of experimental vibrations of VAL and NIC with calculated frequencies along with potential energy distribution (PED, %). ........ 21

**Fig. S11.** Comparison of the experimental FT-IR spectrum of VAL (a) and NIC (b) with the theoretical frequencies. ........24

**Table S7.** Comparison of experimental vibrations of **VAL/NIC slurry** with calculated frequencies. ........25

**Table S8.** Comparison of experimental vibrations of **VAL/NIC LAG 60 min.** with calculated frequencies. ....25

**Table S9.** Comparison of experimental vibrations of **VAL/NIC ball mill** with calculated frequencies. ........25

5.3. Quantum Theory of Atoms in Molecules (QTAIM) ........26

**Table S10.** Topological parameters corresponding to H-bonds involved in intermolecular interactions. ........26

**Table S11.** Geometrical parameters corresponding to H-bonds involved in intermolecular interactions along with molecular graphs with the BCPs (3, -1) as orange dots. ........ 27

**Fig. S12a.** 3D-NCI plot with color-filled reduced density gradient (RDG) isosurfaces depicting non-covalent interactions in VAL/NIC1 and VAL/NIC2 along with 2D-NCI scatter plots (calculated in gas phase). ........28

**Fig. S12b.** 3D-NCI plot with color-filled reduced density gradient (RDG) isosurfaces depicting non-covalent interactions in VAL/NIC1 and VAL/NIC2 along with 2D-NCI scatter plots (calculated in ethanol). ........29

**Table S12.** Topological parameters corresponding to H-bonds involved in intramolecular interactions in homodimers. ........30

**Table S13.** Geometrical parameters determined from X-ray structures, corresponding to H-bonds involved in intramolecular interactions in homodimers. ........30

**Fig. S13.** 3D-NCI plot with color-filled reduced density gradient (RDG) isosurfaces depicting non-covalent interactions in homodimers along with 2D-NCI scatter plots. ........ 31

# 1. Valsartan (VAL)

## 1.1. Extraction of VAL from tablets

Valsartan (VAL) was extracted from tablets of Vanatex (Polpharma SA) and Axudan (Sandoz GmbH). The tablets without envelopes were crushed by using a mortar and pestle, and VAL was extracted with acetonitrile. It was purified by a slurry crystallization from the *n*-heptane and diethyl ether (v/v, 1:1) mixture (30 °C, 72 h) .[1] Then, the mixture was cooled, filtered and the residue was dried under vacuum for 24 h to give pure VAL in 64% yield (m.p. 114-118 °C), which is consistent with literature data.[2]

The $^{1}H$ NMR and $^{13}C$ NMR spectra were measured on a Bruker AV III 500 spectrometer in MeOD with chemical shifts (δ) given in ppm relative to TMS as an internal standard. The $^{13}C$ cross-polarization magic angle spinning ($^{13}C$ CPMAS), ssNMR experiments were performed on a Bruker Avance III 600 spectrometer with resonance frequencies of 150.918 MHz. The spectra were collected with a spinning rate of 12 kHz and the relaxation delays (RDs) during the acquisitions of 5 s and 300 s. The Fourier transformation mid infrared (MIR) spectra of VAL in the region 4000-400 $cm^{-1}$ were measured at 2 $cm^{-1}$ resolution with co-addition of 32 scans on a Nicolet-Nexus spectrometer using the KBr pellet technique. The differential scanning calorimetry (DSC) measurements were performed using a Netzsch STA 409 C/CD instrument. The samples were placed in sealed non-hermetic aluminum pans and scanned at a heating rate 10.0 K/min under an argon atmosphere. High-resolution mass spectrometry (HRMS) measurements were performed using Synapt G2-Si mass spectrometer (Waters) equipped with an ESI source and quadrupole-Time-of-Flight mass analyser. The mass spectrometer was operated in the positive ion detection mode. The measurement was performed with capillary voltage set to 2.7 kV and sampling cone to 20 V. The source temperature was 110 °C. The results of the measurements were processed using the MassLynx 4.1 software (Waters) incorporated with the instrument. X-ray powder diffraction (XRPD) data were collected at room temperature on an Xcalibur™3 CCD diffractometer using CuKα radiation. XRPD experiments were performed using transmission geometry at room temperature with powdered samples sealed in capillary tubes and rotated about Phi over 360° at 0.5°/S. CCD image data were processed by CrysAlisPRO software. The obtained data were analyzed by using the Fullprof software.

## 1.2. DSC analysis

Nalluri *et al.* reported that recrystallization of valsartan from methanol, ethanol, isopropanol, and acetonitrile yields several endothermic (broad) peaks according to their DSC thermograms.[3] The DSC curve of the obtained VAL shows the tallest endothermic peak ($T_m$) at 117.9 °C with enthalpy 29.25 J/g related to the melting point and glass transition ($T_g$) event at 69.8 °C (**Fig. S1**), what is consistent with literature data, where the reported glass transition events of valsartan were at 67, 69 and 78 °C.[4] Decomposition of valsartan starts around 160°C, which is consistent with the experimental DSC curve.

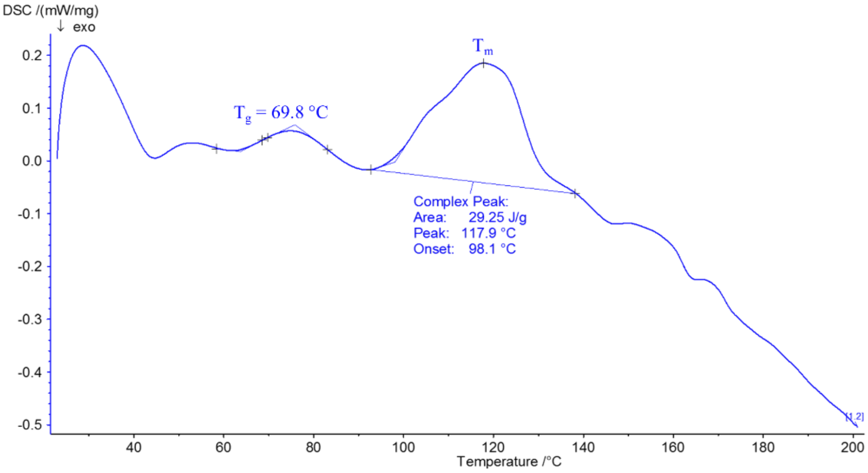


**Fig. S1.** DSC curve of VAL extracted from tablets.

---

[1] J.R. Wang, X. Wang, L. Lu, X. Mei, Highly crystalline forms of valsartan with superior physicochemical stability. *Cryst. Growth Des.*, **2013**, *13 (7)*, 3261-3269.
[2] Merck & Co., Inc., The Merck Index, Merck & Co., Inc., Whitehouse Station, NJ, USA, **2008**.
[3] B.N. Nalluri, R.M. Krishna, T.P. Rao, P.A. Crooks, Effect of recrystallization on the pharmaceutical properties of valsartan for improved therapeutic efficacy, *J. Appl. Pharm. Sci.*, **2012**, *2 (10)*, 126–132.
[4] J. J. M. Ramos, H. P. Diogo, Thermal behavior and molecular mobility in the glassy state of three anti-hypertensive pharmaceutical ingredients, RSC Adv., **2017**, 7, 10831-10840.

## 1.3. FT-IR spectroscopy

The FT-IR spectrum of VAL in the KBr pellet is shown in **Fig. S2** with main peaks and their assignments listed in **Table S1**. The IR characteristic vibrations of VAL are consistent with the literature data.[5,6]

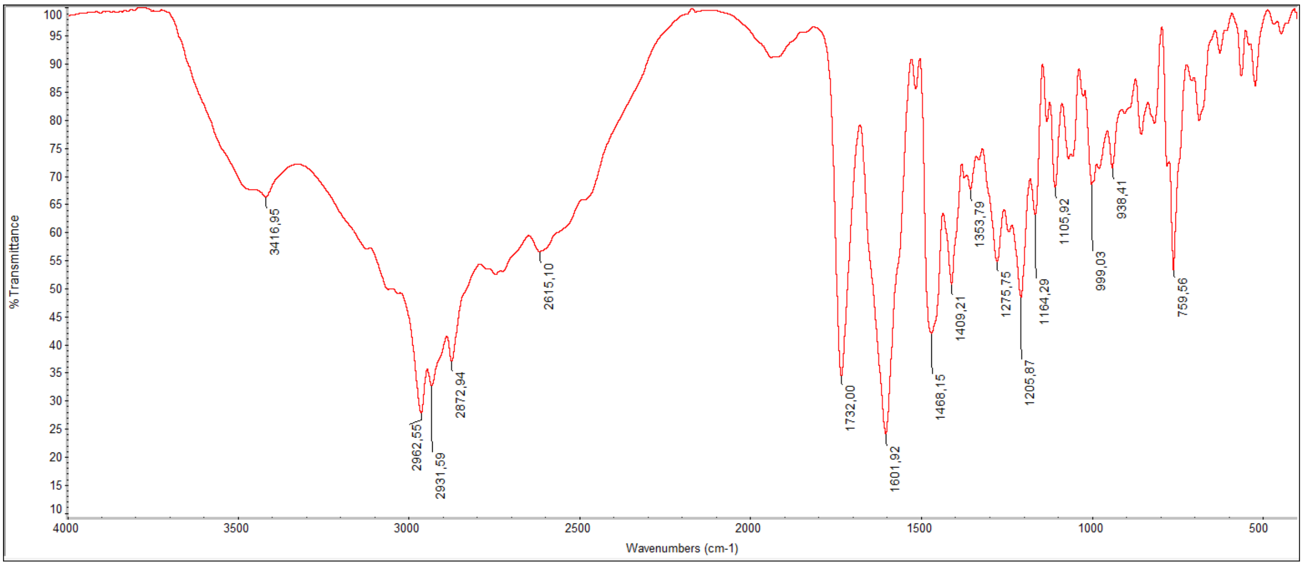


**Fig. S2.** The IR spectrum of VAL extracted from tablets.

**Table S1.** FT-IR characteristic vibrations of isolated VAL compared with the published data [4,5]

| Assignment | Wavenumber [$cm^{-1}$] | |
|---|---|---|
| | Experimental | Literature |
| υ(OH) | 3121 | 3121 [4] |
| υ(NH) | 3416 | 3435 [4] |
| υ(C=O)$^{acid}$ | 1732 | 1733 [4], 1602 [5] |
| υ(C=O)$^{amide}$ | 1602 | 1603 [4], 1602 [5] |
| υ(C-O) | 1396 | 1391 [4] |

υ - stretching

## 1.4. NMR analysis

Two *cis/trans* rotamers of valsartan, major and minor ones have been reported by Li *et al.* who suggested that these species existed as the result of rotation around the amide C(O)-N bond.[7] Further studies were conducted by Chashmniam and Tafazzoli, who identified four different rotamers.[8] Consequently, $^{1}H$ and $^{13}C$ NMR spectra of valsartan indicated two sets of data which are presented in **Fig. S3** and **S4**. Calculated major/minor rotamers ratio is 2 : 1 (m/M = 0.50 ratio) and is consistent with the literature data, where m/M = 0.48 ratio was reported in MeOD.[7]

[5] K. R. Rajeswari, K. Abbulu, M. Sudhakar, R. Karki, B. Rajkumar, Development and characterization of valsartan loaded hydrogel beads, *Pharm. Lett.*, **2012**, *4 (4)*, 1044–1053.
[6] A. Sampath, A.R. Reddy, B. Yakambaran, A. Thirupathi, M. Prabhakar, P.P. Reddy, V.P. Reddy, Identification and characterization of potential impurities of valsartan AT1 receptor antagonist, *J. Pharm. Biomed. Anal.*, **2009**, *50*, 405–412.
[7] F. Li, H. Zhang, L. Jiang, W. Zhang, J. Nie, Y. Feng, M. Yang, M. Liu, Dynamic NMR study and theoretical calculations on the conformational exchange of valsartan and related compounds, *Magn. Reson. Chem.*, **2007**, *45*, 929–936.
[8] S. Chashmniam, M. Tafazzoli, NMR investigation and theoretical calculations of the solvent effect on the conformation of valsartan, *J. Mol. Struct.*, **2017**, *1148*, 73-80.

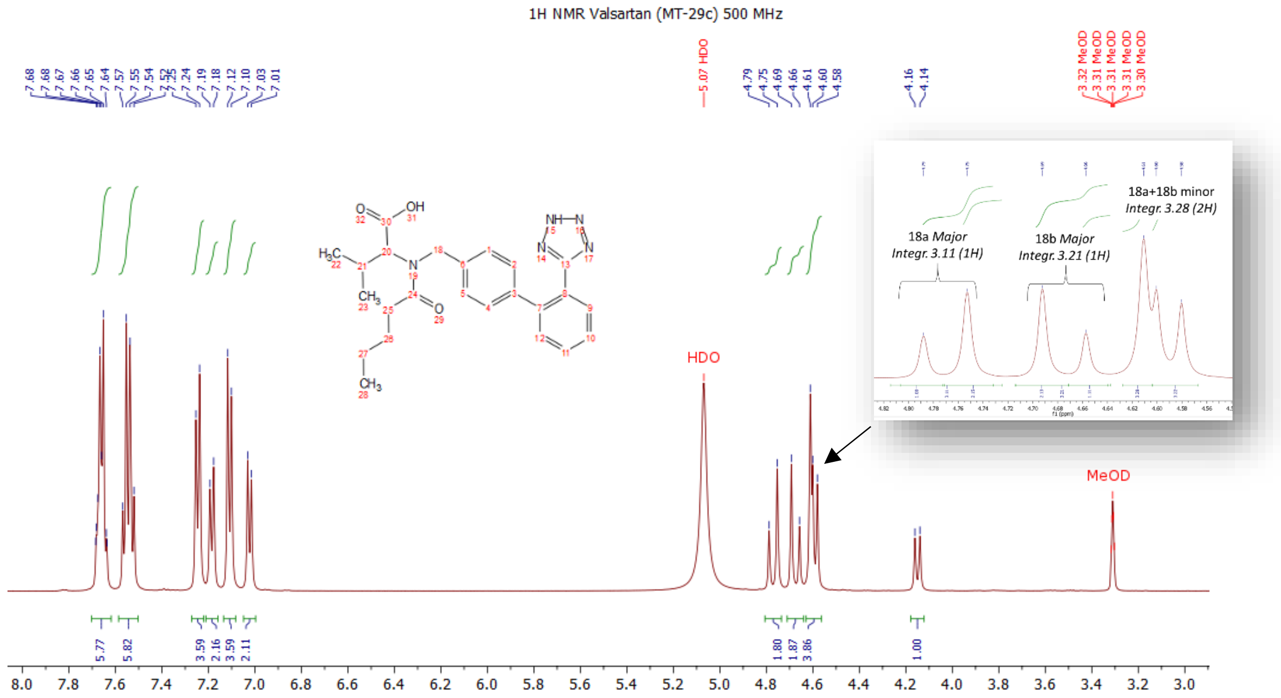


**Fig. S3a.** $^{1}$H NMR (MeOD) spectra of VAL extracted from tablets (δ 8-3 ppm).

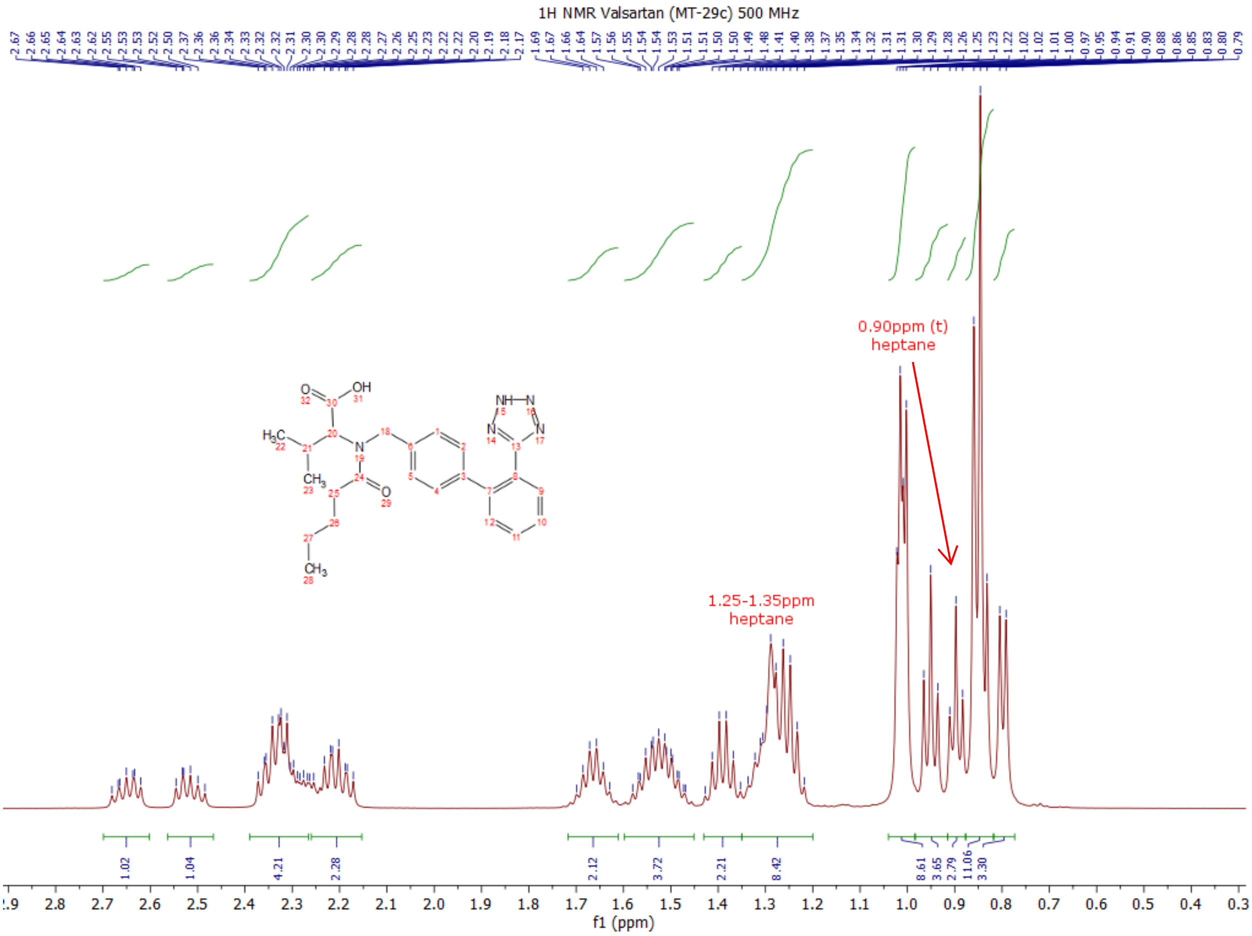


**Fig. S3b.** $^{1}$H NMR (MeOD) spectra of VAL extracted from tablets (δ 3-0 ppm).

* the residual heptane comes from the crystallization process

$^{1}$H NMR (*major conformer*) (500 MHz, MeOD): δ 7.54 (q, *J* = 8.1 Hz, 4H), 7.25 (d, *J* = 7.9 Hz, 1H), 7.24 (s, 1H), 7.12 (s, 1H), 7.11 (d, *J* = 7.9 Hz, 1H), 4.77 (d, *J* = 17.5 Hz, 1H), 4.67 (d, *J* = 17.5 Hz, 1H), 4.59 (d, *J* = 10.0 Hz, 1H), 2.25-

2.37 (m, 1H), 2.33 (s, 1H), 2.17-2.23 (m, 1H), 1.37-1.43 (m, 2H), 1.22-1.34 (m, 2H), 1.01 (d, *J* = 6.5, 3.5 Hz, 3H), 0.83-0.86 (m, 3H), 0.80 (d, *J* = 6.7 Hz, 3H).

$^{1}$H NMR (*minor conformer*) (500 MHz, MeOD): δ 7.65-7.68 (m, 4H), 7.19 (d, *J* = 7.9 Hz, 1H), 7.18 (s, 1H), 7.03 (s, 1H), 7.02 (d, *J* = 7.9 Hz, 1H), 4.61 (s, 2H), 4.15 (d, *J* = 10.7 Hz, 1H), 2.62-2.68 (m, 1H), 2.48-2.55 (m, 1H), 2.22 (s, 1H), 1.63-1.70 (m, 2H), 1.47-1.58 (m, 2H), 1.02 (d, *J* = 3.5 Hz, 3H), 0.95 (t, *J* = 7.4 Hz, 3H), 0.83-0.86 (m, 3H).

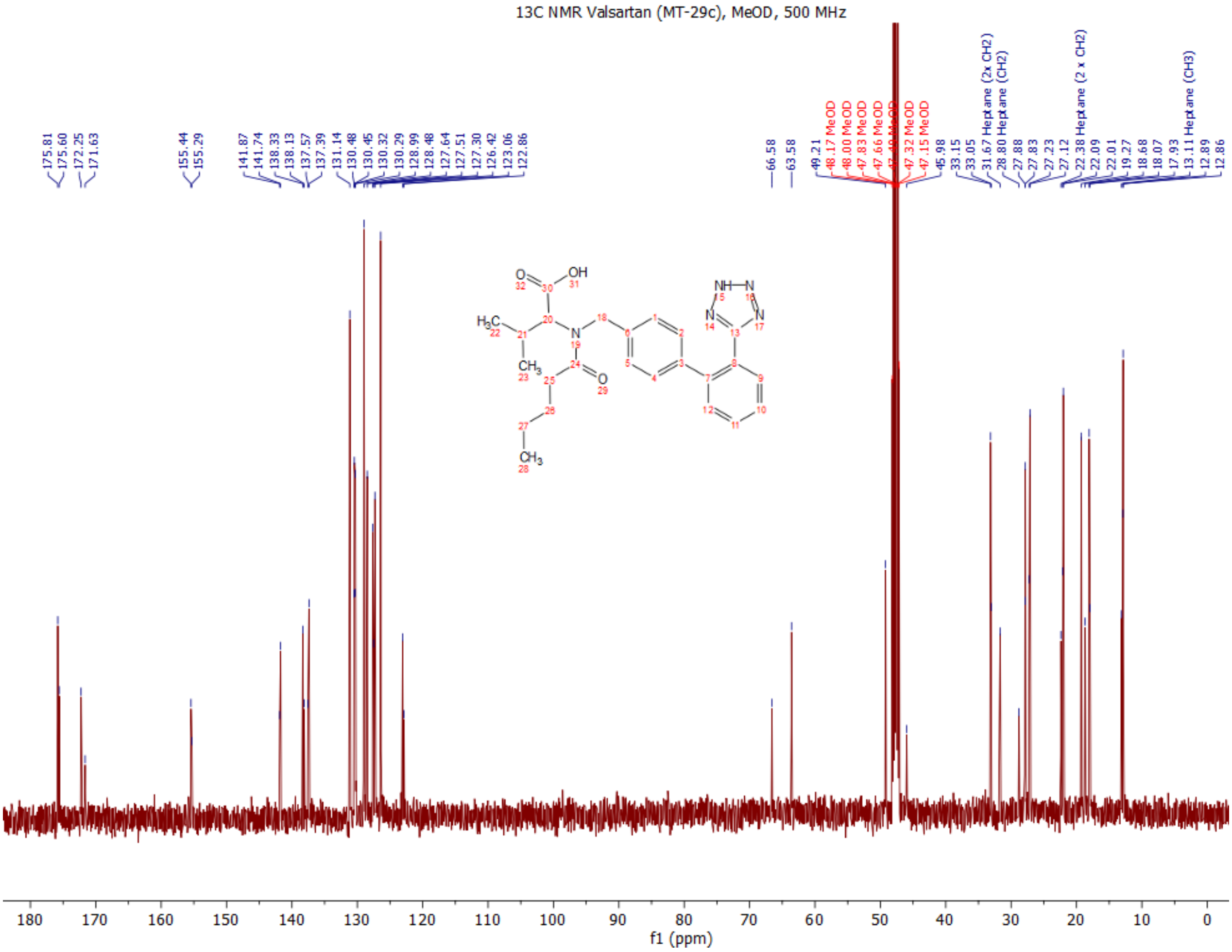


**Fig. S4.** $^{13}$C NMR (MeOD) spectra of VAL extracted from tablets.
* the residual heptane comes from the crystallization process

$^{13}$C NMR (*major conformer*) (126 MHz, MeOD) δ 175.81 (C-24), 172.25 (C-30), 155.44 (C-13), 141.87 (C-7), 138.33 (C-3), 137.57 (C-6), 131.14 (C-9), 130.45 (C-11), 130.32 (C-12) 128.99 (C-4, C-8), 128.48 (C-2), 126.42 (C-1), 127.51 (C-5), 123.06 (C-10), 63.58 (C-20), 49.21 (C-18), 33.15 (C-25), 27.88 (C-21), 27.23 (C-26), 22.09 (C-27), 19.27 (C-22), 18.07 (C-23), 12.89 (C-28).

$^{13}$C NMR (*minor conformer*) (126 MHz, MeOD) δ 175.60 (C-24), 171.63 (C-30), 155.29 (C-13), 141.74 (C-7), 138.13 (C-3), 137.39 (C-6), 131.14 (C-9), 130.48 (C-11), 130.29 (C-12), 128.99 (C-4), 127.64 (C-2), 127.30 (C-5), 126.42 (C-1, C-8), 122.86 (C-10), 66.58 (C-20), 45.98 (C-18), 33.05 (C-25), 27.83 (C-21), 27.12 (C-26), 22.01 (C-27), 18.68 (C-22), 17.93 (C-23), 12.86 (C-28).

## 1.5. ssNMR analysis

The structure of the solid form of VAL was also confirmed by utilizing solid-state NMR (ssNMR). Spectra were recorded with the spinning rate of 12 kHz and relaxation delays (RDs) equal 5 s and 300 s. The $^{13}$C ssNMR spectra of VAL extracted from tablets were compared with the same spectra of the VAL sample purchased from Sigma-Aldrich. The spectra recorded with RDs of 5 s and 300 s were identical, both in case of VAL extracted from tablets and VAL purchased (**Fig. S5**).

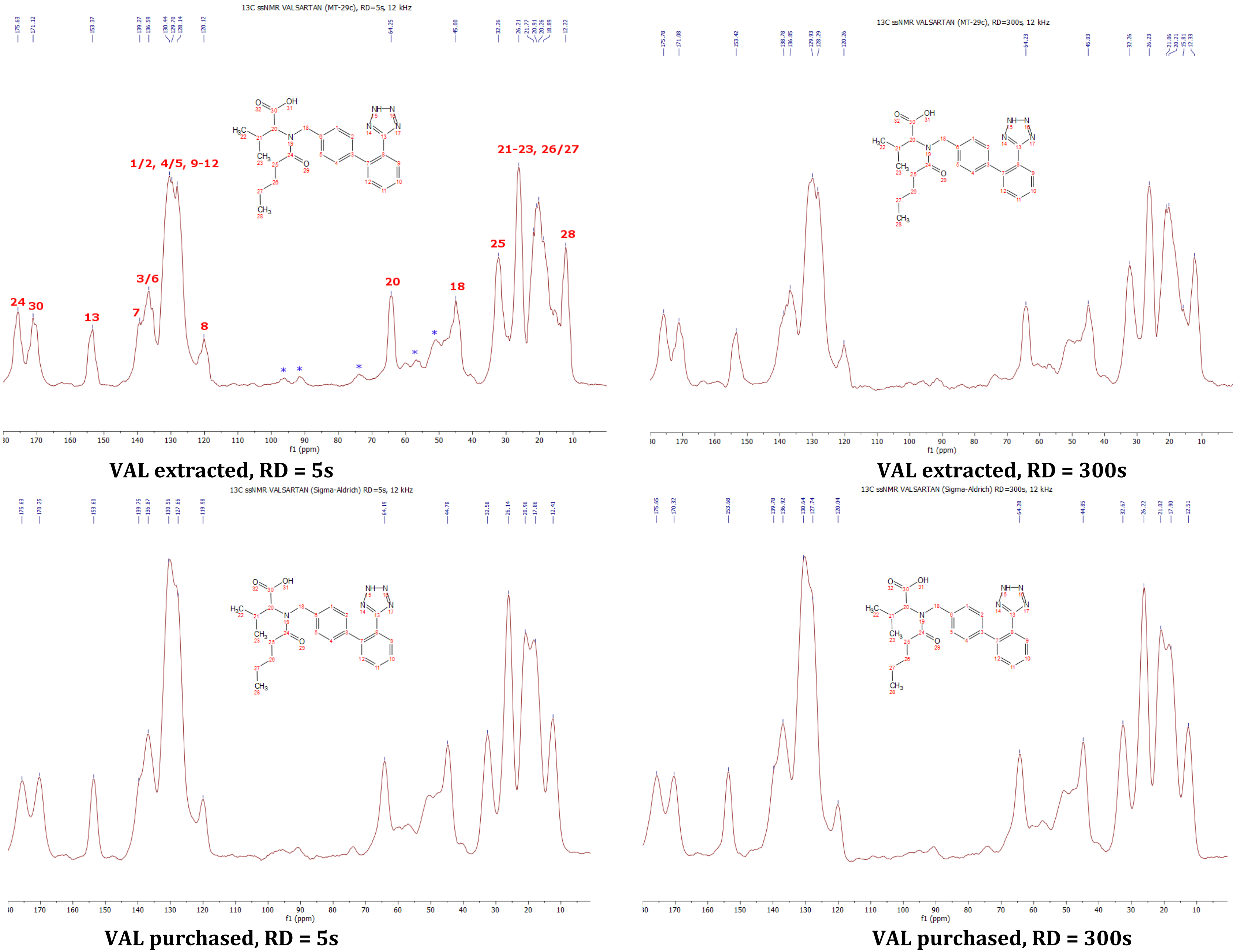


**Fig. S5.** $^{13}$C ssNMR spectra of VAL extracted from tablets and purchased (Sigma-Aldrich, as a reference).Asterisks denote spinning sidebands.

## 1.6. HRMS analysis

The ESI-MS spectrum of VAL used in this study is shown in **Fig. S6**. The MS data are similar to that described in the literature.[9] The important fragments (*m/z*) are 436 (valsartan (acid), $[M+H]^+$) and 458 (valsartan sodium salt, $[M+Na]^+$).

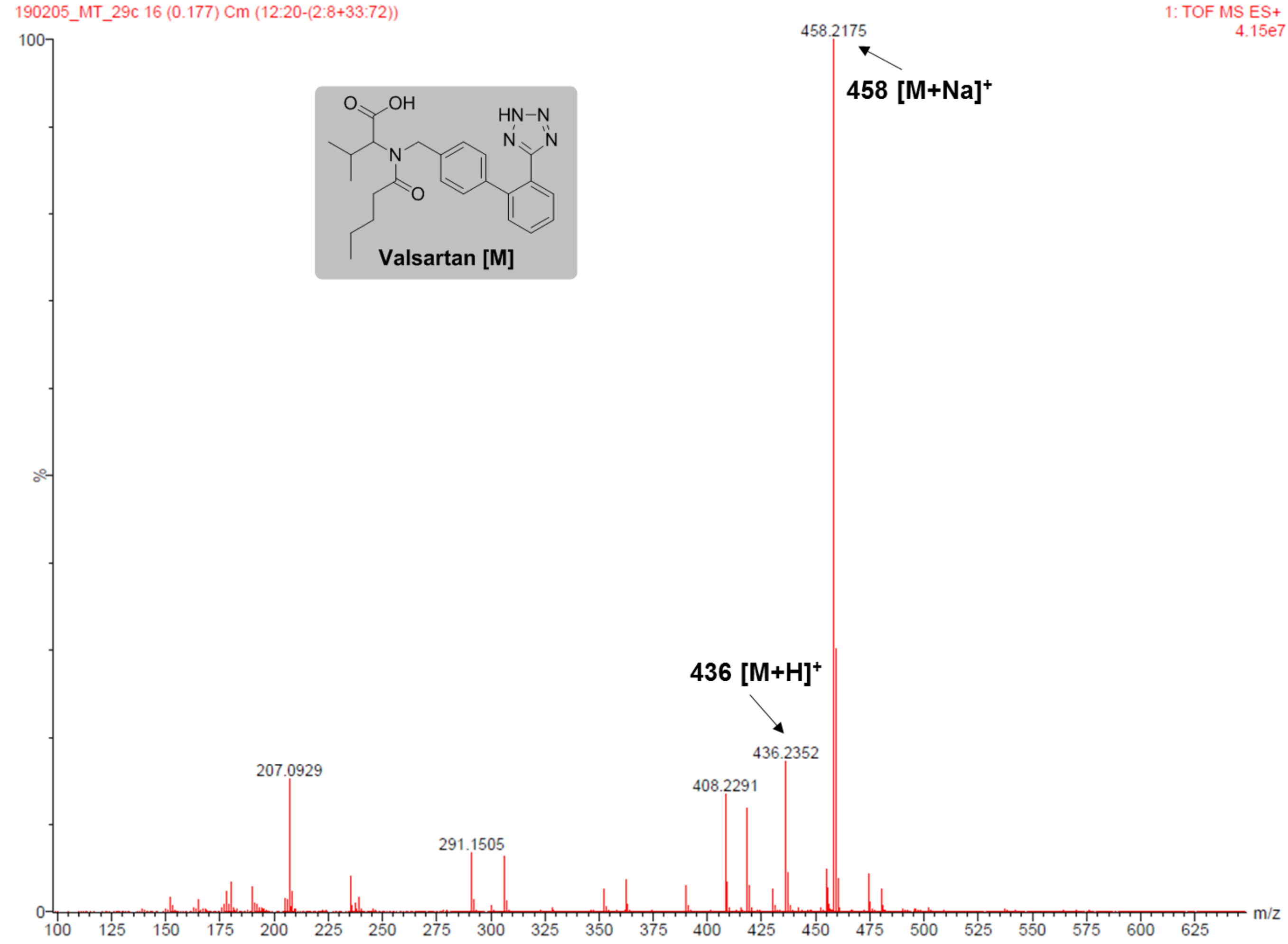


**Fig. S6.** MS spectrum of VAL extracted from tablets.

MS - ES (+): m/z (%) = 436 [$(M + H)^+$, 20]; 458 [$(M + Na)^+$, 100]
HRMS (TOF MS ES+) (m/z): calcd for $C_{24}H_{30}N_5O_3$ $[M+H]^+$: 436.2349; found: 436.2352.
HRMS (TOF MS ES+) (m/z): calcd for $C_{24}H_{29}N_5O_3Na$ $[M+Na]^+$: 458.2168; found: 458.2175.

## 1.7. XRPD

According to the literature, VAL may occur in 12 different polymorphs and an amorphous form which can be distinguished from each other with X-ray powder diffraction pattern.[10] The XRPD pattern of VAL extracted from tablets and two literature forms are presented in **Fig. S7**. VAL used in this study is characterized by an XRPD pattern with broad amorphous halo together with several reflections at 12.60, 14.84, 17.68, 22.19, 25.57, 27.23° which correspond to VAL form VII reflections described in the literature.[10] So it can be concluded that VAL extracted from tablets occurs mostly in amorphous form with the addition of polymorphic form VII.

[9] N. Koseki, H. Kawashita, H. Hara, M. Niina, M. Tanaka, R. Kawai, Y. Nagae, N. Masuda, Development and validation of a method for quantitative determination of valsartan in human plasma by liquid chromatography-tandem mass spectrometry, *J. Pharm. Biomed. Anal.,* **2007**, *43*, 1769–1774.
[10] I. Rukhman, T. Flyaks, J. Koltai, R. Aronhime, Polymorphs of valsartan, US Patent 7105557 B2, 12.09.2006

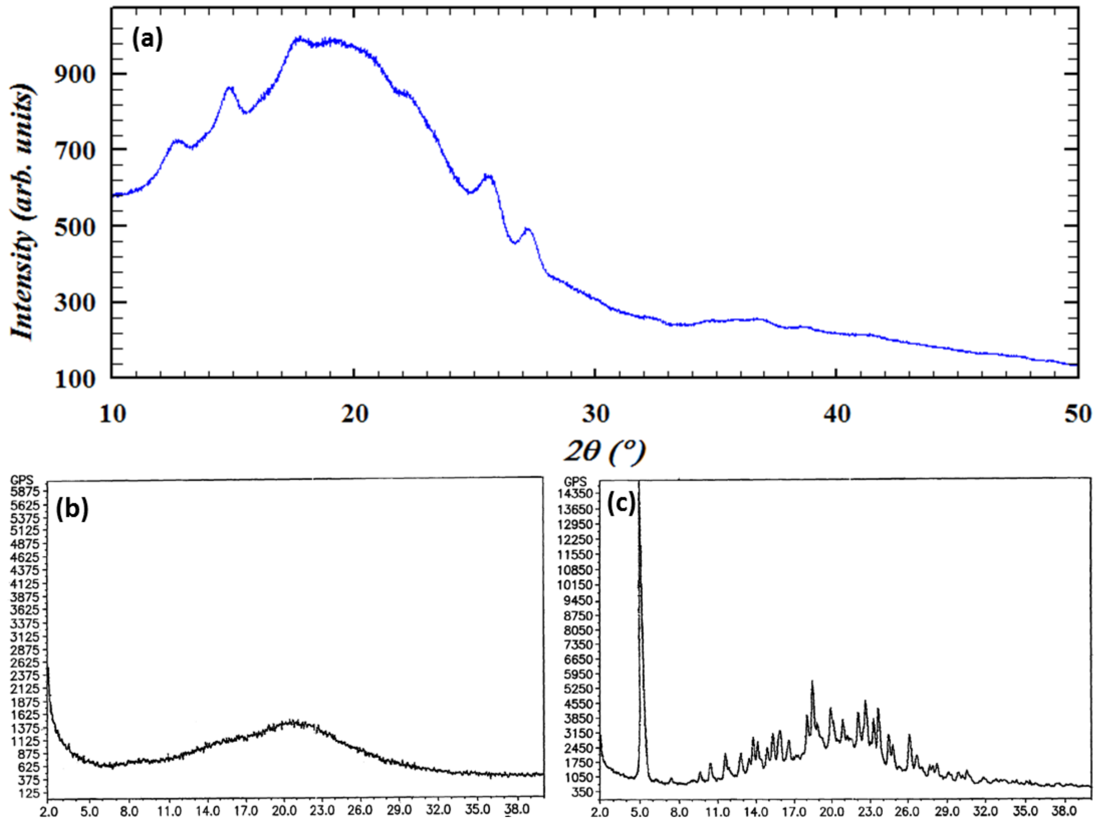


**Fig. S7.** XRPD pattern of (a) VAL used in this study; (b) VAL purely amorphous[10]; (c) VAL polymorphic form VII[10].

# 2. Nicotinamide (NIC)

## 2.1. XRPD

The XRPD of the NIC sample used in this study can be accurately matched to the simulated XRPD pattern which was generated from the deposited X-ray data file at the Cambridge Structure Database (CSD – Refcode NICOAM06) using the Mercury software (**Fig. S8**).

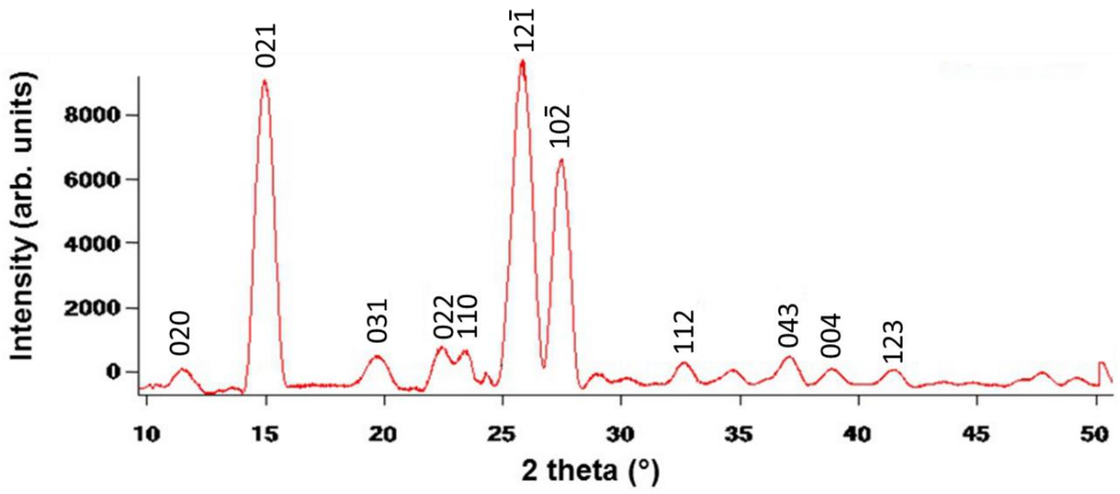


**Fig. S8.** XRPD pattern of NIC used in this study along with designation of major reflections.

# 3. Solid dispersions (VAL/NIC)

## 3.1. Solution-state NMR studies

The solution-state $^1H$ NMR spectra ($CDCl_3$) were collected for co-amorphous solid dispersions to quantitatively estimate the amount of ethanol in obtained formulations. The spectra are presented in **Fig. S8**. The calculated composition of obtained materials is the following:

- VAL/NIC slurry –VAL 47.78%, NIC 47.78%, EtOH 4.43% (by moles); VAL 77.51%, NIC 21.73%, EtOH 0.76% (by weight);
- VAL/NIC LAG 30 min - VAL 49.26%, NIC 49.26%, EtOH 1.47% (by moles); VAL 77.91%, NIC 21.85%, EtOH 0.25% (by weight);
- VAL/NIC LAG 60 min - VAL 47.14%, NIC 47.14%, EtOH 5.73% (by moles); VAL 77.32%, NIC 21.68%, EtOH 0.99% (by weight);
- VAL/NIC ball mill - VAL 46.61%, NIC 46.61%, EtOH 6.79% (by moles); VAL 77.17%, NIC 21.64%, EtOH 1.19% (by weight).

$^1H$ NMR spectra in $CDCl_3$ show two valsartan rotamers (N and n) that differ from those mentioned in MeOD (**Fig. S3a**). Chashmniam and Tafazzoli reported valsartan's n/N rotamers ratio in $CDCl_3$ = 0.22.[8]

The major/minor rotamers ratios in currently investigated solid dispersions are the following:

- VAL/NIC slurry – 1.7 : 1.0 (n/N = 0.59)
- VAL/NIC LAG 30 min – 2.6 : 1.0 (n/N = 0.38)
- VAL/NIC LAG 60 min – 2.0 : 1.0 (n/N = 0.50)
- VAL/NIC ball mill – 2.3 : 1.0 (n/N = 0.43)

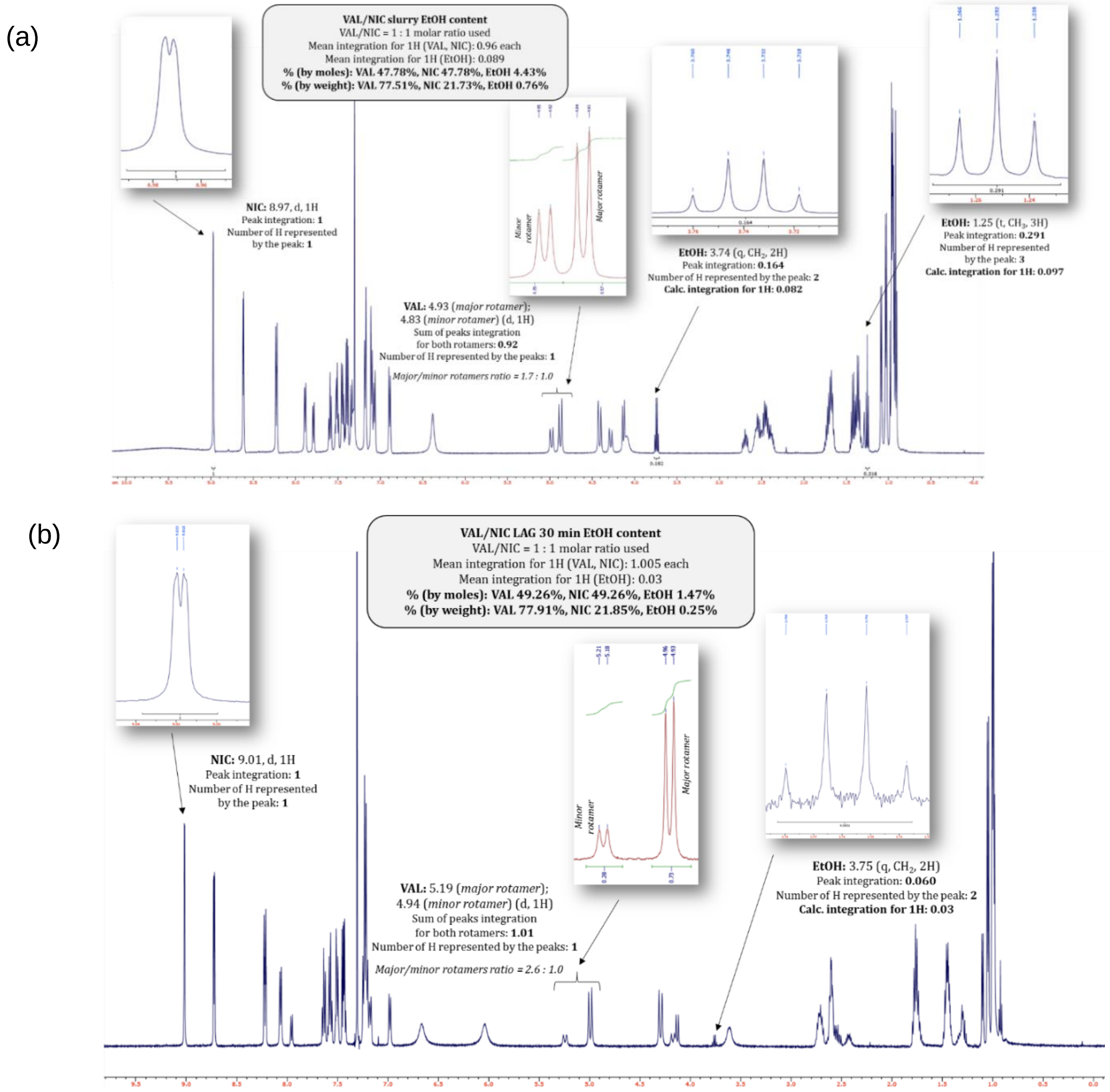

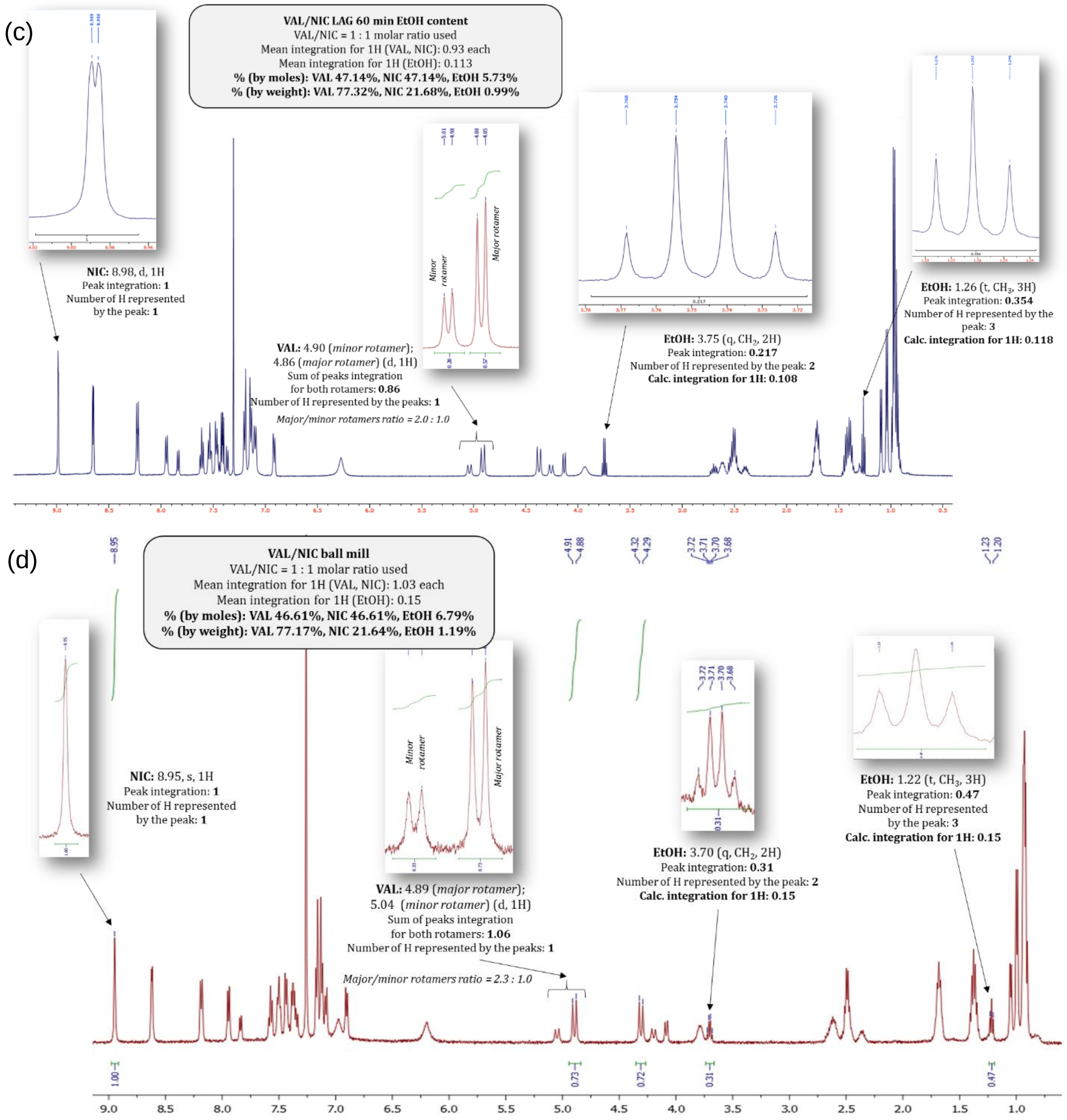


**Fig. S9.** $^1$H NMR ($CDCl_3$) spectra for (a) VAL/NIC slurry, (b) VAL/NIC LAG 30 min, (c) VAL/NIC LAG 60 min, (d) VAL/NIC ball mill along with calculations of the EtOH content.

# 4. Preliminary semi-empirical calculations

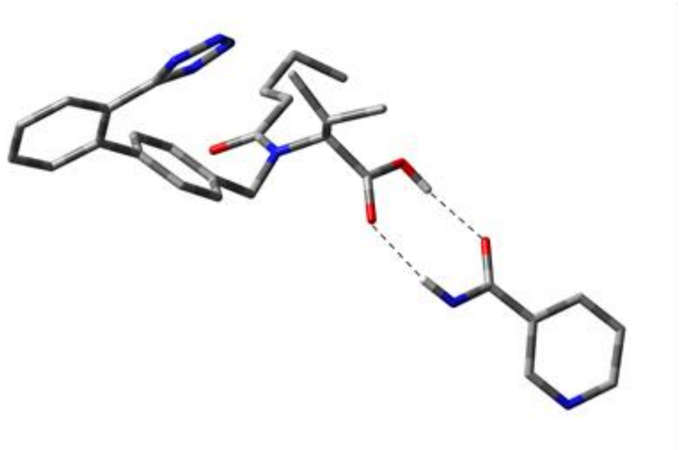

**VAL/NIC1**
Total energy: -0.01474049

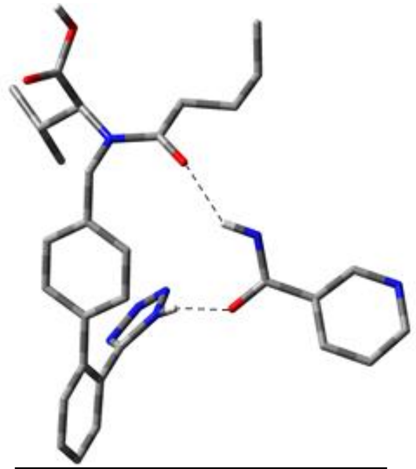

**VAL/NIC2**
Total energy: -0.01466401

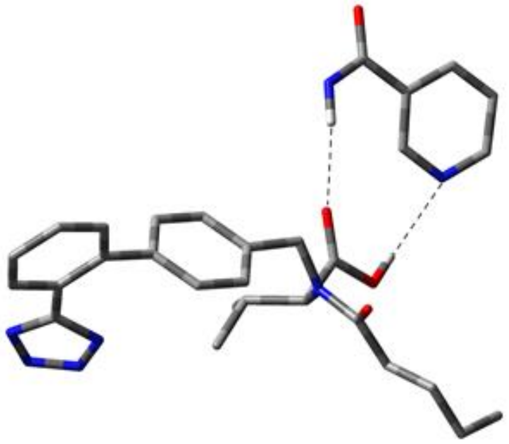

**VAL/NIC3**
Total energy: -0.01072872

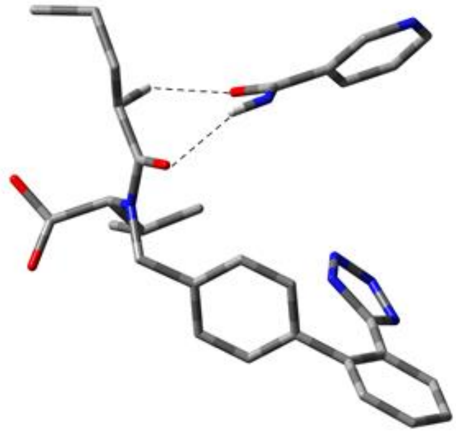

**VAL/NIC4**
Total energy: -0.01058827

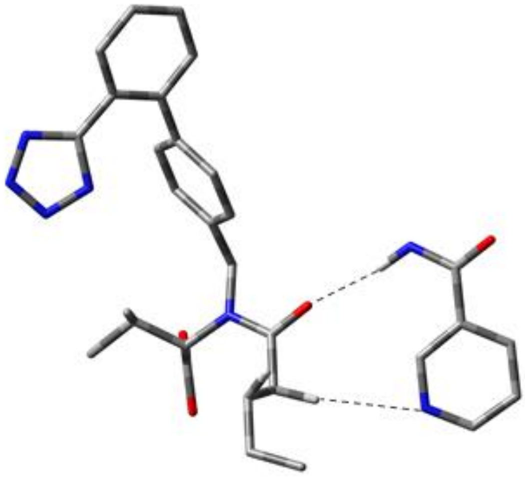

**VAL/NIC5**
Total energy: -0.01123121

**Fig. S10.** Chemical structures of 5 possible VAL/NIC heterodimers optimized by using AM1 method along with calculated total energy (Hartree).

# 5. DFT calculations

## 5.1. Cartesian coordinates and total energies for the optimized structures

**Table S2.** Atom coordinates (Å), total energy (Hartree) and the number of imaginary vibrational frequencies for the geometry of **VAL** optimized at the B3LYP/6-311++(d,p) level in the gas phase using Gaussian 09.

| Atom | X | Y | Z |
|---|---|---|---|
| O | -2.7684850 | -2.4280240 | -0.5030770 |
| O | -4.9688980 | 1.5794580 | -1.4970850 |
| H | -5.3945520 | 1.8758640 | -2.3173040 |
| O | -3.1402920 | 2.1579170 | -2.6673180 |
| N | 2.9384080 | 1.1781310 | 1.2818500 |
| H | 2.1291850 | 0.6245730 | 1.0399720 |
| N | -2.4567900 | -0.1953950 | -0.7193170 |
| N | 2.8065650 | 2.3493290 | 1.9351560 |
| N | 4.0017540 | 2.8172600 | 2.0718510 |
| N | 4.9172680 | 1.9849780 | 1.5246770 |
| C | 4.2465240 | 0.9555310 | 1.0245500 |
| C | 4.8506680 | -0.2160610 | 0.3748400 |
| C | 4.1286850 | -1.1295350 | -0.4237600 |
| C | 2.6848860 | -0.9496460 | -0.7664370 |
| C | 2.2806840 | 0.0319720 | -1.6787940 |
| H | 3.0230590 | 0.6750980 | -2.1386440 |
| C | 0.9340020 | 0.1908710 | -1.9987020 |
| H | 0.6458840 | 0.9587390 | -2.7097350 |
| C | -0.0479470 | -0.6214580 | -1.4232470 |
| C | -1.5031680 | -0.4500070 | -1.8230730 |
| H | -1.5848220 | 0.3718810 | -2.5333660 |
| H | -1.8420010 | -1.3600440 | -2.3214920 |
| C | -3.0951540 | -1.2967750 | -0.1706330 |
| C | -4.1982210 | -1.0656280 | 0.8610490 |
| H | -4.9806410 | -0.4495160 | 0.4078120 |
| H | -3.7910490 | -0.4804830 | 1.6949360 |
| C | -4.7866400 | -2.3753890 | 1.3947050 |
| H | -3.9737230 | -2.9969220 | 1.7788290 |
| H | -5.2239960 | -2.9384480 | 0.5643000 |
| C | -5.8377730 | -2.1699640 | 2.4957720 |
| H | -6.1036290 | -3.1529540 | 2.8992770 |
| H | -5.3895770 | -1.6147110 | 3.3296310 |
| C | -7.1176950 | -1.4603180 | 2.0387080 |
| H | -6.9255250 | -0.4351150 | 1.7098640 |
| H | -7.8483930 | -1.4087520 | 2.8504620 |
| H | -7.5861090 | -1.9934880 | 1.2049580 |
| C | 1.7032670 | -1.7780520 | -0.2040420 |
| H | 1.9983910 | -2.5553360 | 0.4928750 |
| C | 0.3572390 | -1.6183210 | -0.5277980 |
| H | -0.3858440 | -2.2787860 | -0.0978260 |
| C | 6.2197620 | -0.4279240 | 0.6043990 |
| H | 6.7556240 | 0.2862730 | 1.2164440 |
| C | 6.8690490 | -1.5323840 | 0.0709270 |
| H | 7.9256710 | -1.6800980 | 0.2611530 |
| C | 6.1556720 | -2.4499970 | -0.6982060 |
| H | 6.6519030 | -3.3191620 | -1.1147450 |
| C | 4.8010830 | -2.2439340 | -0.9395240 |
| H | 4.2480400 | -2.9452850 | -1.5538880 |

| | | | |
|---|---|---|---|
| C | -2.8612560 | 1.1957770 | -0.4370990 |
| H | -3.5643530 | 1.1689970 | 0.3909030 |
| C | -3.6308990 | 1.7172030 | -1.6580800 |
| C | -1.7009510 | 2.1320370 | -0.0308550 |
| H | -0.9702140 | 2.1459390 | -0.8445350 |
| C | -2.2125760 | 3.5670770 | 0.1731850 |
| H | -2.9853710 | 3.6028650 | 0.9491180 |
| H | -2.6245730 | 3.9933650 | -0.7437770 |
| H | -1.3935660 | 4.2130590 | 0.4975700 |
| C | -1.0207830 | 1.6235050 | 1.2471300 |
| H | -0.1615670 | 2.2507890 | 1.4978270 |
| H | -0.6770970 | 0.5928060 | 1.1424730 |
| H | -1.7109370 | 1.6624420 | 2.0971520 |
| Total energy | | -1431.83718410 | |
| Number of imaginary vibrational frequencies | | 0 | |

**Table S3.** Atom coordinates (Å), total energy (Hartree) and the number of imaginary vibrational frequencies for the geometry of **NIC** optimized at the B3LYP/6-311++(d,p) level in the gas phase using Gaussian 09.

| Atom | X | Y | Z |
|---|---|---|---|
| C | 1.7046700 | 0.1438590 | -0.0298970 |
| N | 2.4042190 | -1.0056210 | 0.2221040 |
| O | 2.2627750 | 1.2006900 | -0.2746680 |
| H | 3.4073790 | -0.9208750 | 0.2850020 |
| H | 1.9733640 | -1.8069080 | 0.6521700 |
| C | 0.2057170 | 0.0343940 | -0.0103280 |
| C | -0.4915110 | -1.1705240 | -0.1493110 |
| C | -0.5423180 | 1.2070130 | 0.1226570 |
| H | 0.0387280 | -2.1064230 | -0.3046240 |
| C | -1.9274670 | 1.1214020 | 0.1391840 |
| H | -0.0234880 | 2.1542570 | 0.2067700 |
| H | -2.5427080 | 2.0066600 | 0.2472230 |
| N | -1.8231190 | -1.2655150 | -0.1387610 |
| C | -2.5195110 | -0.1352910 | 0.0114710 |
| H | -3.6006590 | -0.2393950 | 0.0247490 |
| Total energy | | -417.10989111 | |
| Number of imaginary vibrational frequencies | | 0 | |

**Table S4a.** Atom coordinates (Å), total energy (Hartree) and the number of imaginary vibrational frequencies for the geometry of **VAL/NIC1** optimized at the B3LYP/6-311++(d,p) level in the **gas phase** using Gaussian 09.

| Atom | X | Y | Z |
|---|---|---|---|
| O | 0.0916940 | 2.7215260 | -2.0646030 |
| O | 3.3184970 | 0.9107470 | 0.8369690 |
| H | 4.1610180 | 0.3989040 | 0.6350960 |
| O | 2.2791030 | -1.0535550 | 0.4903180 |
| N | -4.9289920 | -0.4874130 | 1.6536810 |
| H | -4.2580300 | 0.0505370 | 1.1243090 |
| N | 0.3874900 | 1.2620780 | -0.3588480 |
| N | -4.7940730 | -0.6432530 | 2.9853530 |
| N | -5.7764920 | -1.3930280 | 3.3574680 |
| N | -6.5546150 | -1.7364050 | 2.3047570 |
| C | -6.0176190 | -1.1691140 | 1.2328870 |
| C | -6.5668400 | -1.2348910 | -0.1286650 |
| C | -5.8144500 | -0.9581430 | -1.2910120 |

| | | | |
|---|---|---|---|
| C | -4.3503620 | -0.6623770 | -1.2636670 |
| C | -3.4218180 | -1.6574890 | -0.9360400 |
| H | -3.7694130 | -2.6552850 | -0.6912140 |
| C | -2.0575550 | -1.3763530 | -0.9207140 |
| H | -1.3576160 | -2.1650500 | -0.6633890 |
| C | -1.5763530 | -0.1005190 | -1.2309920 |
| C | -0.0810480 | 0.1668640 | -1.2370880 |
| H | 0.4478410 | -0.7402580 | -0.9480350 |
| H | 0.2264780 | 0.4288380 | -2.2512010 |
| C | 0.5031070 | 2.5189090 | -0.9292560 |
| C | 1.1419970 | 3.6397180 | -0.1109880 |
| H | 2.1491390 | 3.3294410 | 0.1818210 |
| H | 0.5773220 | 3.7688850 | 0.8206810 |
| C | 1.1859190 | 4.9690540 | -0.8701920 |
| H | 0.1735350 | 5.2252840 | -1.1931690 |
| H | 1.7669180 | 4.8396550 | -1.7885480 |
| C | 1.7687450 | 6.1256150 | -0.0437730 |
| H | 1.6302460 | 7.0527650 | -0.6105450 |
| H | 1.1846960 | 6.2470090 | 0.8775810 |
| C | 3.2539610 | 5.9785840 | 0.3075840 |
| H | 3.4431100 | 5.1139660 | 0.9499180 |
| H | 3.6208010 | 6.8624660 | 0.8367890 |
| H | 3.8603810 | 5.8578410 | -0.5959300 |
| C | -3.8679480 | 0.6114440 | -1.5965560 |
| H | -4.5704730 | 1.3920540 | -1.8688450 |
| C | -2.5025530 | 0.8889390 | -1.5822170 |
| H | -2.1460690 | 1.8738360 | -1.8582130 |
| C | -7.9271230 | -1.5630060 | -0.2442630 |
| H | -8.4843200 | -1.7753950 | 0.6592690 |
| C | -8.5477510 | -1.6008970 | -1.4851260 |
| H | -9.5992780 | -1.8537560 | -1.5543880 |
| C | -7.8164250 | -1.3032560 | -2.6336070 |
| H | -8.2927210 | -1.3240190 | -3.6072320 |
| C | -6.4654690 | -0.9875740 | -2.5303920 |
| H | -5.8891970 | -0.7778910 | -3.4243350 |
| C | 0.9430730 | 0.9226220 | 0.9676690 |
| H | 1.2205890 | 1.8557300 | 1.4504330 |
| C | 2.2477620 | 0.1402950 | 0.7514630 |
| C | -0.0455950 | 0.1930640 | 1.9042080 |
| H | -0.3610370 | -0.7340500 | 1.4170350 |
| C | 0.6420470 | -0.1781580 | 3.2281200 |
| H | 1.0206010 | 0.7137240 | 3.7394080 |
| H | 1.4758210 | -0.8676240 | 3.0802800 |
| H | -0.0724200 | -0.6620570 | 3.8982260 |
| C | -1.2800700 | 1.0633320 | 2.1729970 |
| H | -2.0003930 | 0.5222990 | 2.7921940 |
| H | -1.7748700 | 1.3628220 | 1.2473390 |
| H | -1.0030890 | 1.9742120 | 2.7152900 |
| C | 5.8005660 | -1.5043180 | 0.0215810 |
| N | 4.8002180 | -2.4001590 | -0.0605900 |
| O | 5.5987750 | -0.3153530 | 0.3101840 |
| H | 3.8468410 | -2.0786190 | 0.1191900 |
| H | 4.9779810 | -3.3812660 | -0.1920550 |
| C | 7.1964120 | -1.9737200 | -0.2463120 |
| C | 7.4966800 | -3.1467920 | -0.9476810 |

| | | | |
|---|---|---|---|
| C | 8.2640830 | -1.1890800 | 0.1981670 |
| H | 6.7073790 | -3.7708410 | -1.3590220 |
| C | 9.5602250 | -1.6194600 | -0.0482240 |
| H | 8.0570150 | -0.2644420 | 0.7224700 |
| H | 10.4147160 | -1.0436990 | 0.2862670 |
| N | 8.7379510 | -3.5680260 | -1.1938080 |
| C | 9.7453640 | -2.8157410 | -0.7411060 |
| H | 10.7457610 | -3.1848090 | -0.9473280 |
| Total energy | | -1848.971109 | |
| Number of imaginary vibrational frequencies | | 0 | |

**Table S4b.** Atom coordinates (Å), total energy (Hartree) and the number of imaginary vibrational frequencies for the geometry of **VAL/NIC1** optimized at the B3LYP/6-311++(d,p) level in **ethanol** using Gaussian 09.

| Atom | X | Y | Z |
|---|---|---|---|
| O | 0.1789950 | 2.8987800 | -1.8287450 |
| O | 3.3726180 | 0.7996950 | 0.9166200 |
| H | 4.1987480 | 0.2807480 | 0.6582710 |
| O | 2.2711650 | -1.0716450 | 0.3414150 |
| N | -5.1163990 | -1.0441820 | 1.7132840 |
| H | -4.5745290 | -0.2178300 | 1.4974410 |
| N | 0.4040940 | 1.3289220 | -0.2119180 |
| N | -4.9089590 | -1.7234550 | 2.8529140 |
| N | -5.7381470 | -2.7136670 | 2.8337010 |
| N | -6.4858930 | -2.7021340 | 1.7057800 |
| C | -6.0890600 | -1.6489660 | 1.0002370 |
| C | -6.6653960 | -1.2002440 | -0.2743720 |
| C | -5.9198350 | -0.5278140 | -1.2678560 |
| C | -4.4469170 | -0.3076790 | -1.1808480 |
| C | -3.5568850 | -1.3851240 | -1.0866480 |
| H | -3.9386730 | -2.3994900 | -1.0493990 |
| C | -2.1814070 | -1.1683460 | -1.0525870 |
| H | -1.5140510 | -2.0214120 | -0.9839880 |
| C | -1.6494440 | 0.1238220 | -1.1145510 |
| C | -0.1422830 | 0.3128120 | -1.1374880 |
| H | 0.3417860 | -0.6359940 | -0.9140560 |
| H | 0.1611820 | 0.6070590 | -2.1439450 |
| C | 0.5773950 | 2.6062750 | -0.7014690 |
| C | 1.2553880 | 3.6465990 | 0.1860060 |
| H | 2.2680850 | 3.3008120 | 0.4130520 |
| H | 0.7279110 | 3.6966040 | 1.1452350 |
| C | 1.2933180 | 5.0412170 | -0.4454980 |
| H | 0.2724790 | 5.3466340 | -0.6918730 |
| H | 1.8365420 | 4.9974250 | -1.3948140 |
| C | 1.9319830 | 6.1030790 | 0.4633330 |
| H | 1.7750150 | 7.0845270 | 0.0027960 |
| H | 1.3993780 | 6.1295120 | 1.4220890 |
| C | 3.4319780 | 5.9098020 | 0.7153500 |
| H | 3.6431380 | 4.9792050 | 1.2494850 |
| H | 3.8356290 | 6.7292800 | 1.3166500 |
| H | 3.9870670 | 5.8850140 | -0.2281240 |
| C | -3.9142890 | 0.9876670 | -1.2482270 |
| H | -4.5839780 | 1.8373100 | -1.3237730 |
| C | -2.5382760 | 1.1997920 | -1.2155940 |
| H | -2.1528900 | 2.2095880 | -1.2783900 |
| C | -8.0365460 | -1.4322160 | -0.4641810 |

| | | | |
|---|---|---|---|
| H | -8.5924460 | -1.9445070 | 0.3108430 |
| C | -8.6812530 | -0.9909540 | -1.6126090 |
| H | -9.7412400 | -1.1742340 | -1.7414300 |
| C | -7.9585090 | -0.3023790 | -2.5856210 |
| H | -8.4507360 | 0.0518960 | -3.4839060 |
| C | -6.5962350 | -0.0783380 | -2.4099330 |
| H | -6.0321660 | 0.4329610 | -3.1811750 |
| C | 0.9947950 | 0.8717700 | 1.0658730 |
| H | 1.3054490 | 1.7553510 | 1.6153400 |
| C | 2.2743450 | 0.0819430 | 0.7440960 |
| C | 0.0202760 | 0.0835220 | 1.9693430 |
| H | -0.3451430 | -0.7834560 | 1.4110440 |
| C | 0.7464700 | -0.4292840 | 3.2237790 |
| H | 1.1622050 | 0.4014930 | 3.8037280 |
| H | 1.5585880 | -1.1184680 | 2.9820300 |
| H | 0.0437150 | -0.9628320 | 3.8682030 |
| C | -1.1736700 | 0.9595190 | 2.3718300 |
| H | -1.8805510 | 0.3776850 | 2.9688680 |
| H | -1.7064800 | 1.3513800 | 1.5047830 |
| H | -0.8436570 | 1.8073190 | 2.9813130 |
| C | 5.8279910 | -1.5281710 | -0.1575570 |
| N | 4.8228500 | -2.3851970 | -0.3821240 |
| O | 5.6334690 | -0.3790900 | 0.2831550 |
| H | 3.8700570 | -2.0814120 | -0.1816530 |
| H | 4.9861300 | -3.3454180 | -0.6386770 |
| C | 7.2234160 | -1.9788250 | -0.4518570 |
| C | 7.5182170 | -2.9846380 | -1.3785420 |
| C | 8.2910140 | -1.3514960 | 0.1963020 |
| H | 6.7275060 | -3.4729170 | -1.9401230 |
| C | 9.5849450 | -1.7688980 | -0.0838410 |
| H | 8.0951470 | -0.5576180 | 0.9059370 |
| H | 10.4383440 | -1.3155830 | 0.4046280 |
| N | 8.7601550 | -3.3878470 | -1.6614140 |
| C | 9.7686370 | -2.7907470 | -1.0141860 |
| H | 10.7675850 | -3.1418410 | -1.2536410 |
| Total energy | | -1848.99834351 | |
| Number of imaginary vibrational frequencies | | 0 | |

**Table S5a.** Atom coordinates (Å), total energy (Hartree) and the number of imaginary vibrational frequencies for the geometry of **VAL/NIC2** optimized at the B3LYP/6-311++(d,p) level in the gas phase using Gaussian 09.

| Atom | X | Y | Z |
|---|---|---|---|
| O | 1.5577490 | 1.5359160 | -0.8428210 |
| O | 6.0016830 | 1.0442570 | -1.5588140 |
| H | 6.5142280 | 1.1464500 | -2.3763780 |
| O | 5.6347020 | -0.9774440 | -2.4690790 |
| N | -1.8477440 | -1.1229230 | 1.8864450 |
| H | -1.9554200 | -0.3167770 | 1.2590190 |
| N | 3.1784990 | -0.0269350 | -0.7351790 |
| N | -1.1804150 | -1.0029770 | 3.0468900 |
| N | -1.2751240 | -2.1450030 | 3.6451580 |
| N | -1.9982760 | -3.0218080 | 2.9048250 |
| C | -2.3474400 | -2.3723800 | 1.8045770 |
| C | -3.1631690 | -2.9100710 | 0.6999330 |

| | | | |
|---|---|---|---|
| C | -2.7327230 | -2.8618820 | -0.6433000 |
| C | -1.3815850 | -2.3575090 | -1.0122400 |
| C | -0.2306810 | -2.9091250 | -0.4345380 |
| H | -0.3225030 | -3.7394490 | 0.2563520 |
| C | 1.0281720 | -2.4006020 | -0.7336110 |
| H | 1.8978100 | -2.8548190 | -0.2743710 |
| C | 1.1782430 | -1.3143500 | -1.6005690 |
| C | 2.5411020 | -0.7140540 | -1.8901630 |
| H | 3.2308830 | -1.4924310 | -2.2136380 |
| H | 2.4494080 | 0.0014460 | -2.7111050 |
| C | 2.5810300 | 1.1307910 | -0.2938780 |
| C | 3.1930190 | 1.8807750 | 0.8861650 |
| H | 4.1394370 | 2.3214580 | 0.5534380 |
| H | 3.4429620 | 1.1699940 | 1.6803220 |
| C | 2.2688210 | 2.9666770 | 1.4462510 |
| H | 1.3226550 | 2.5056600 | 1.7428520 |
| H | 2.0274580 | 3.6809070 | 0.6531790 |
| C | 2.8631850 | 3.7131670 | 2.6505690 |
| H | 2.0802500 | 4.3514440 | 3.0734200 |
| H | 3.1186970 | 2.9930560 | 3.4374600 |
| C | 4.0864340 | 4.5788970 | 2.3272320 |
| H | 4.9379390 | 3.9828320 | 1.9863810 |
| H | 4.4153470 | 5.1358550 | 3.2087010 |
| H | 3.8561180 | 5.3069730 | 1.5425870 |
| C | -1.2255530 | -1.3168480 | -1.9340400 |
| H | -2.1018640 | -0.8745070 | -2.3942210 |
| C | 0.0353580 | -0.7995870 | -2.2149750 |
| H | 0.1295680 | 0.0429290 | -2.8904130 |
| C | -4.4082470 | -3.4674610 | 1.0146920 |
| H | -4.7126240 | -3.5096970 | 2.0535170 |
| C | -5.2458080 | -3.9465490 | 0.0134430 |
| H | -6.2079330 | -4.3735990 | 0.2717710 |
| C | -4.8428050 | -3.8695970 | -1.3182460 |
| H | -5.4889810 | -4.2393680 | -2.1065440 |
| C | -3.5978760 | -3.3357220 | -1.6375980 |
| H | -3.2698450 | -3.3111680 | -2.6707270 |
| C | 4.5512540 | -0.4136680 | -0.3484290 |
| H | 4.8858300 | 0.2931940 | 0.4063760 |
| C | 5.4516870 | -0.1936470 | -1.5730780 |
| C | 4.6769710 | -1.8331870 | 0.2471040 |
| H | 4.2698130 | -2.5456490 | -0.4778920 |
| C | 6.1505750 | -2.1987210 | 0.4892520 |
| H | 6.6297030 | -1.4781760 | 1.1614580 |
| H | 6.7232630 | -2.2412610 | -0.4385310 |
| H | 6.2167480 | -3.1801470 | 0.9647950 |
| C | 3.8847720 | -1.9324120 | 1.5584910 |
| H | 3.8796420 | -2.9626860 | 1.9230970 |
| H | 2.8477380 | -1.6135710 | 1.4463150 |
| H | 4.3462570 | -1.3154590 | 2.3369030 |
| C | -2.3112690 | 2.2191540 | -0.2462460 |
| N | -1.1243720 | 2.6693900 | -0.6969740 |
| O | -2.4459230 | 1.1345350 | 0.3247750 |
| H | -0.2730380 | 2.1179300 | -0.5990330 |
| H | -1.0642080 | 3.5051810 | -1.2535200 |
| C | -3.5106280 | 3.0981110 | -0.4690240 |

| | | | |
|---|---|---|---|
| C | -3.4338060 | 4.4695750 | -0.7362070 |
| C | -4.7822100 | 2.5267140 | -0.3755070 |
| H | -2.4746660 | 4.9796870 | -0.7730800 |
| C | -5.8969180 | 3.3283900 | -0.5791950 |
| H | -4.8697150 | 1.4723320 | -0.1442950 |
| H | -6.8987600 | 2.9199400 | -0.5218320 |
| N | -4.4974390 | 5.2519350 | -0.9293750 |
| C | -5.7034440 | 4.6809110 | -0.8585470 |
| H | -6.5525410 | 5.3368900 | -1.0273060 |
| Total energy | | -1848.964769 | |
| Number of imaginary vibrational frequencies | | 0 | |

**Table S5b.** Atom coordinates (Å), total energy (Hartree) and the number of imaginary vibrational frequencies for the geometry of **VAL/NIC2** optimized at the B3LYP/6-311++(d,p) level in **ethanol** using Gaussian 09.

| Atom | X | Y | Z |
|---|---|---|---|
| O | 1.5195280 | 1.2771300 | -0.6289240 |
| O | 5.9237050 | 0.9925450 | -1.6668570 |
| H | 6.3630020 | 1.1253750 | -2.5233970 |
| O | 5.5518250 | -1.0459680 | -2.5347090 |
| N | -2.3053050 | -0.8737060 | 1.7672900 |
| H | -2.2977880 | -0.1886230 | 0.9943840 |
| N | 3.2192040 | -0.2027440 | -0.6006740 |
| N | -1.8760460 | -0.5067400 | 2.9833570 |
| N | -2.0627500 | -1.5225280 | 3.7625620 |
| N | -2.6118760 | -2.5562500 | 3.0831190 |
| C | -2.7580280 | -2.1408970 | 1.8299070 |
| C | -3.3662160 | -2.9168590 | 0.7341970 |
| C | -2.7494720 | -3.0736650 | -0.5265320 |
| C | -1.3891380 | -2.5485470 | -0.8248050 |
| C | -0.2982980 | -2.8414420 | 0.0049730 |
| H | -0.4346280 | -3.4813200 | 0.8695720 |
| C | 0.9674260 | -2.3340040 | -0.2726640 |
| H | 1.7889310 | -2.5863480 | 0.3857200 |
| C | 1.1824000 | -1.5047760 | -1.3764740 |
| C | 2.5490730 | -0.9338000 | -1.7031360 |
| H | 3.2190430 | -1.7413160 | -1.9968000 |
| H | 2.4549810 | -0.2678870 | -2.5649780 |
| C | 2.6081330 | 0.9456220 | -0.1553390 |
| C | 3.2836370 | 1.7736280 | 0.9310810 |
| H | 4.1918410 | 2.2133610 | 0.5057590 |
| H | 3.6114340 | 1.1140780 | 1.7406910 |
| C | 2.3804600 | 2.8730500 | 1.4978550 |
| H | 1.4672190 | 2.4150610 | 1.8885470 |
| H | 2.0688730 | 3.5414280 | 0.6892820 |
| C | 3.0473130 | 3.6938650 | 2.6127750 |
| H | 2.2871090 | 4.3480070 | 3.0530720 |
| H | 3.3704830 | 3.0214180 | 3.4168500 |
| C | 4.2321500 | 4.5515620 | 2.1533930 |
| H | 5.0634830 | 3.9436630 | 1.7852920 |
| H | 4.6149650 | 5.1600080 | 2.9774370 |
| H | 3.9347990 | 5.2314900 | 1.3482540 |
| C | -1.1608830 | -1.7609450 | -1.9602180 |
| H | -1.9861060 | -1.5184270 | -2.6202740 |
| C | 0.1031820 | -1.2429150 | -2.2242340 |

| | | | |
|---|---|---|---|
| H | 0.2446720 | -0.6023170 | -3.0881580 |
| C | -4.6199260 | -3.4936770 | 0.9797630 |
| H | -5.0765950 | -3.3631710 | 1.9533630 |
| C | -5.2847480 | -4.2022600 | -0.0146710 |
| H | -6.2556280 | -4.6381570 | 0.1887620 |
| C | -4.6968690 | -4.3376920 | -1.2712970 |
| H | -5.2052020 | -4.8865020 | -2.0557580 |
| C | -3.4452800 | -3.7810880 | -1.5171790 |
| H | -2.9788590 | -3.9181340 | -2.4858290 |
| C | 4.6430890 | -0.5052450 | -0.3278760 |
| H | 5.0003980 | 0.2297560 | 0.3877100 |
| C | 5.4232180 | -0.2566670 | -1.6288620 |
| C | 4.8903480 | -1.9060210 | 0.2757200 |
| H | 4.4186690 | -2.6517440 | -0.3724920 |
| C | 6.3933120 | -2.2228630 | 0.3516720 |
| H | 6.9228480 | -1.4707860 | 0.9461870 |
| H | 6.8584090 | -2.2734530 | -0.6340480 |
| H | 6.5420530 | -3.1899000 | 0.8379710 |
| C | 4.2707580 | -1.9934030 | 1.6783490 |
| H | 4.3282050 | -3.0180650 | 2.0534870 |
| H | 3.2232470 | -1.6886350 | 1.6929090 |
| H | 4.8154020 | -1.3523740 | 2.3790850 |
| C | -2.2556360 | 2.3233810 | -0.4188990 |
| N | -0.9958440 | 2.7611490 | -0.5653810 |
| O | -2.5320980 | 1.1597430 | -0.0910960 |
| H | -0.1986940 | 2.1367110 | -0.4422300 |
| H | -0.8039910 | 3.6760910 | -0.9413380 |
| C | -3.3621520 | 3.3053420 | -0.6737700 |
| C | -3.2116440 | 4.6867110 | -0.5107710 |
| C | -4.6185200 | 2.8261100 | -1.0541880 |
| H | -2.2690790 | 5.1075690 | -0.1736610 |
| C | -5.6444270 | 3.7334440 | -1.2817730 |
| H | -4.7723060 | 1.7604750 | -1.1694060 |
| H | -6.6284460 | 3.4017330 | -1.5888980 |
| N | -4.1951670 | 5.5682340 | -0.7167100 |
| C | -5.3844880 | 5.0914760 | -1.1043270 |
| H | -6.1645720 | 5.8268840 | -1.2753330 |
| Total energy | | -1848.99399892 | |
| Number of imaginary vibrational frequencies | | 0 | |

## 5.2.Vibrational spectra

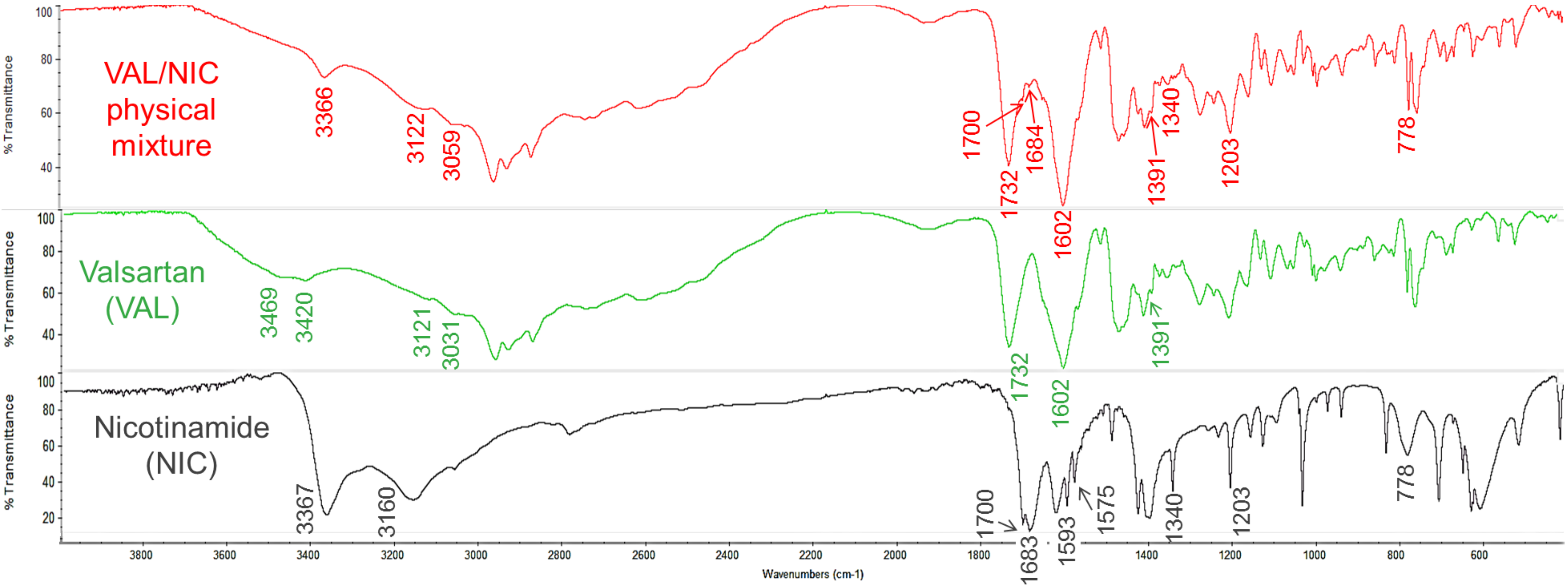


**Fig. S11.** FT-IR spectra recorded for VAL, NIC and their physical mixture.

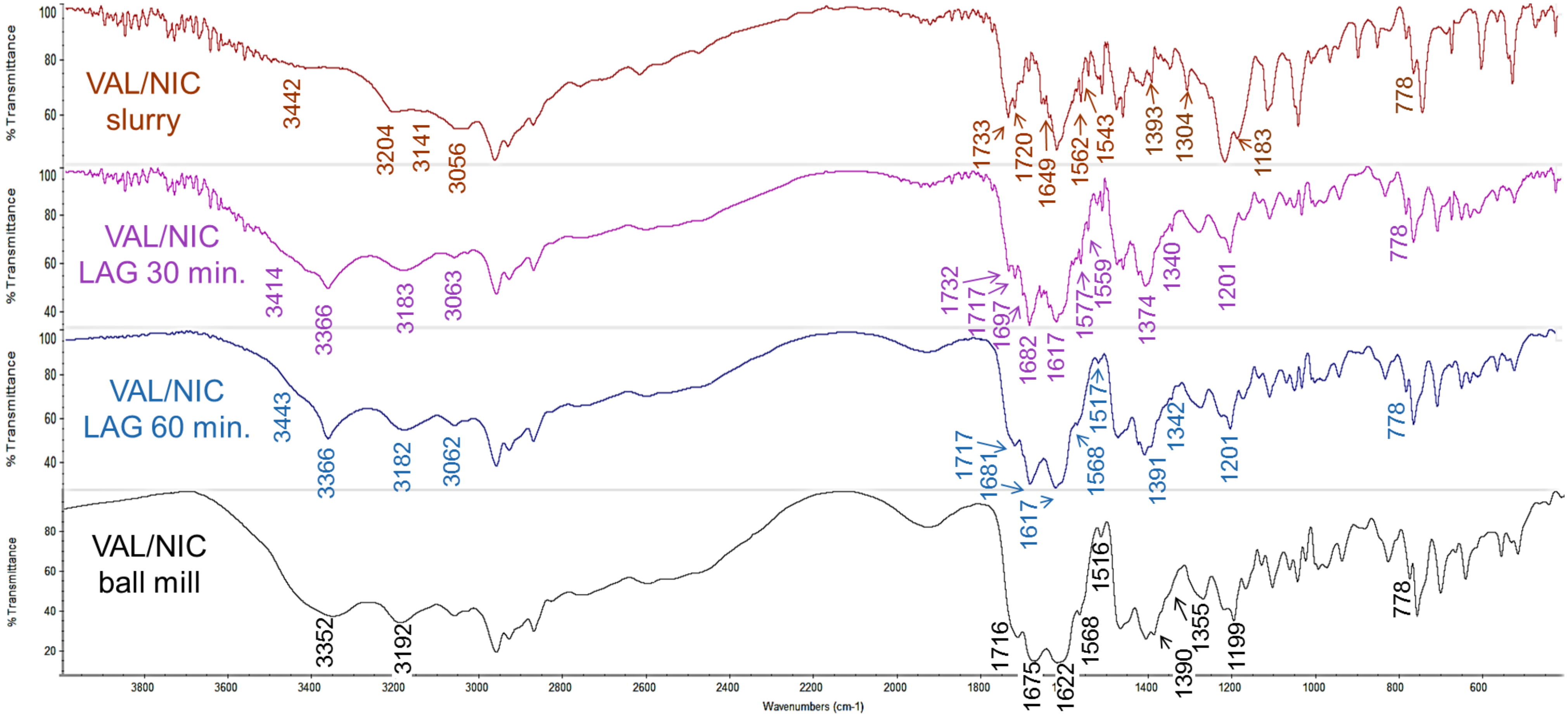


**Fig. S12.** FT-IR spectra recorded for VAL/NIC co-amorphous formulations.

**Table S6.** Comparison of experimental vibrations of VAL and NIC with calculated frequencies along with potential energy distribution (PED, %).[11]*

| Atom numbering | Observed frequencies [$cm^{-1}$] | Calculated frequencies [$cm^{-1}$] | Modes, PED [%] |
|---|---|---|---|
| 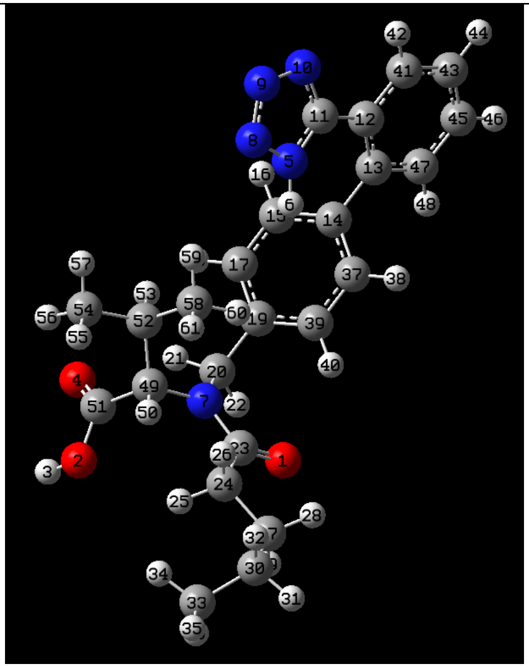 | 3420 | 3508 | ν(H6N5)(99) |
| | 3121 | 3617 | ν(H3O2)(100) |
| | 1732 | 1745 | ν(C51O4)(85), δ(H3O2C51)(4), ν(C51C49)(3), δ(O2C51C49)(2) |
| | 1602 | 1651 | ν(C23O1)(85), δ(N7C23C24)(2), ν(C24C23)(2), δ(C23N7C49)(1) |
| | 1471 | 1351 | ν(C11N10)(26), ν(C11N5)(19), δ(N9N10C11)(13), δ(N8N5C11)(6) |
| | 1458 | 1323 | δ(N7C20H22)(16), δ(C19C20H22)(10), δ(H3O2C51)(3), wag-tors(H33C20C19C39)(3) |
| | 1410 | 1320 | δ(H6N5N8)(20), ν(N9N8)(18), ν(C12C11)(13), δ(H6N5C11)(6) |
| | 1391 | 1238 | δ(H3O2C51)(39), δ(H50C49C51)(7), ν(C51O4)(6), δ(H50C49N7C23)(12), |
| | 1372 | 1234 | ν(N9N8)(17), ν(C41C12)(14), δ(C12C41H42)(14), ν(C14C13)(10) |
| | 1353 | 1215 | ν(C23N7)(16), ν(C20N7)(7), δ(C23N7C49)(3), δ(O1C23N7)(3) |
| | 1333 | 1211 | ν(N9N8)(31), δ(N8N9N10)(13), δ(H6N5C11)(12), ν(C41C12)(5) |
| | 1276 | 1131 | ν(C49N7)(25), ν(C20N7)(8), δ(C19C20H22)(4), δ(C51O2)(4) |
| | 1276 | 1131 | ν(C11N5)(13), δ(N8N9N10)(12), ν(C11N10)(9), ν(N10N9)(6) |
| | 1206 | 1072 | ν(C51O2)(30), δ(H3O2C51)(9), δ(C52C58H61)(4), ν(C51C49)(4), |
| | 1162 | 1056 | ν(N10N9)(25), ν(N8N5)(24), δ(N5N8N9)(16), δ(N8N5C11)(9) |
| | 1130 | 1030 | ν(N8N5)(38), δ(N5N8N9)(20), δ(H6N5C11)(10), δ(H6N5N8)(7) |
| | 1106 | 990 | ν(N10N9)(26), δ(N5N8N9)(5), δ(N9N10C11)(5), ν(C11N5)(5) |
| | 1066 | 987 | ν(N10N9)(12), ν(C45C43)(5), ν(C47C45)(5), ν(C43C41)(5) |
| | 1052 | 981 | δ(N9N10C11)(19), δ(N5C11N10)(13), δ(N8N9N10)(8), δ(N8N5C11)(4) |
| | 857 | 718 | τ(C11N10N9N8)(20), τ(N5C11N10N9)(15), τ(N10N9N8N5)(10), τ(C13C12C11C10)(4) |
| | 778 | 701 | τ(N9N8N5C11)(21), τ(N10N9N8N5)(19), wag-tors(N9N8N5H6)(18), τ(N10C11N5N8)(10) |
| **Valsartan (VAL)** | 686 | 634 | δ(O2C51O4)(29), δ(O4C51C49)(13), ν(C51O2)(10), δ(H3O2C51)(5) |
| 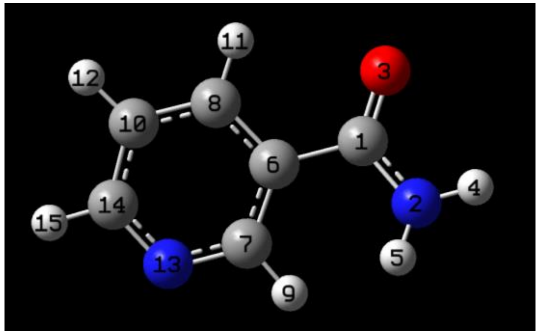 | 3367 | 3592 | ν(H5N2)(58), ν(H4N2)(41) asym |
| | 3160 | 3471 | ν(H4N2)(58), ν(H5N2)(41) sym |
| | 1683 | 1688 | ν(O3C1)(76), ν(N2C1)(5), δ(C1N2H4)(4), δ(N2C1C6)(3) |
| | 1593, 1575 | 1568 | δ(H4N2H5)(57), δ(C1N2H5)(19), δ(C1N2H4)(10), ν(O3C1)(5) |
| | 1203 | 1047 | δ(C1N2H5)(32), ν(N2C1)(23), δ(C1N2H4)(22), ν(O3C1)(9) |
| | 778 | 538 | wag-tors (H4N2C1C6)(37), wag-tors (H4N1C1O3)(29), wag-tors (H5N2C1C6)(10), wag-tors (H5N2C1O3)(6) |
| **Nicotinamide (NIC)** | 703 | 310 | wag-tor (H5N2C1O3)(40), wag-tor (H5N2C1C6)(34), wag-tor (H4N2C1C6)(10), wag-tors (H4N2C1O3)(7) |

υ - stretching, δ - bending, τ – twisting;

*PED matrices were calculated by using the FCART program

[11] C. R. Legler, N. R. Brown, R. A. Dunbar, M. D. Harness, K. Nguyen, O. Oyewole, W. B. Collier, Scaled quantum mechanical scale factors for vibrational calculations using alternate polarized and augmented basis sets with the B3LYP density functional calculation model, Spectrochim. Acta Part A 145 (2015) 15–24 (FCART version 7.0, http://fcart.wbcollier.com/).

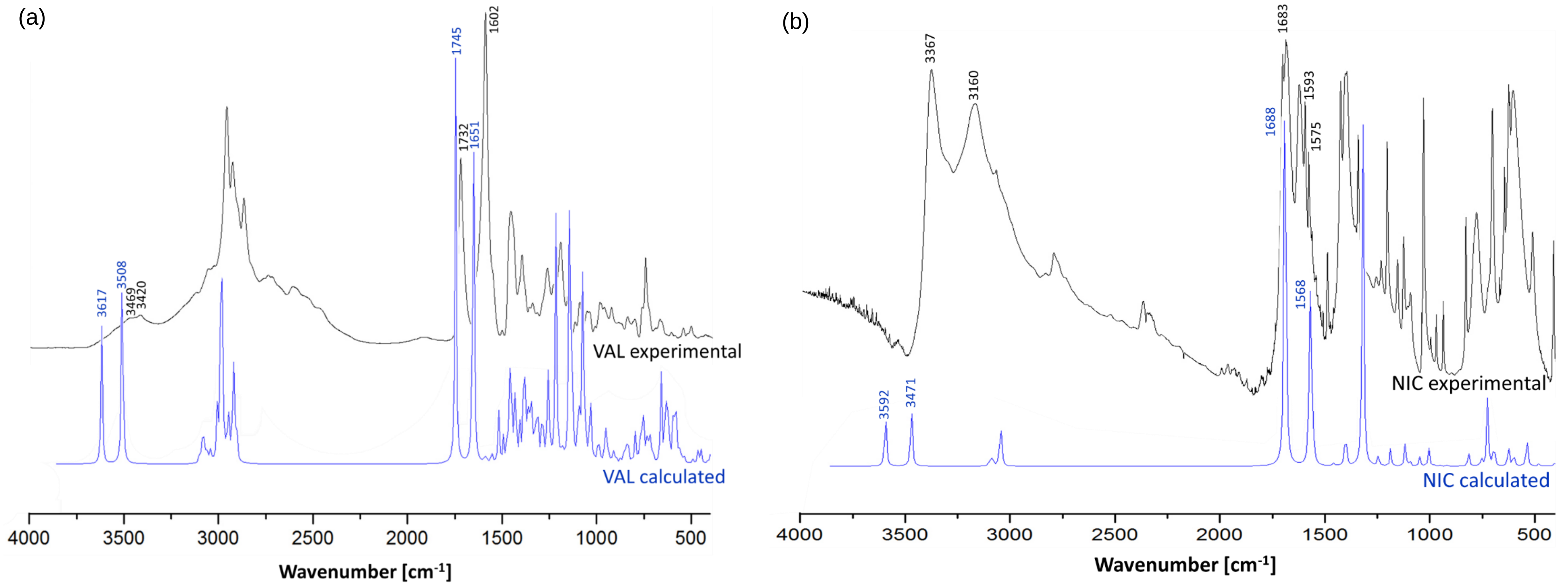


**Fig. S13.** Comparison of the experimental FT-IR spectrum of VAL (a) and NIC (b) with the theoretical frequencies.

**Table S7.** Comparison of experimental vibrations of **VAL/NIC slurry** with calculated frequencies.

| Assignment | Observed frequencies [$cm^{-1}$] | Calculated frequencies [$cm^{-1}$] | |
|---|---|---|---|
| | VAL/NIC slurry | VAL/NIC 1 | VAL/NIC 2 |
| υ(OH) | 3204 | - | 3619 (Δ = -177) |
| $υ(NH)^{tetrazole}$ | 3141 | 3508 (Δ = -367) | 3227 (Δ = -86) |
| **$υ(C=O)^{acid}$** | **1733** | **1686 (Δ = +47)** | **1751 (Δ = -18)** |
| **$υ(C=O)^{amide}$** | **1649** | **1647 (Δ = +2)** | **1624 (Δ = +25)** |
| υ(C-O) | 1393 | 1204 (Δ = +189) | 1073 (Δ = +320) |
| $υ(NH_2)^{as}$ | 3204 | 3565 (Δ = -361) | 3564 (Δ = -360) |
| $υ(NH_2)^{s}$ | 3056 | 3278 (Δ = -222) | 3340 (Δ = -284) |
| **υ(C=O)** | **1720** | **1646 (Δ = +74)** | **1663 (Δ = +57)** |
| δ(NH) | 1562 | 1569 (Δ = -7) | 1596 (Δ = -34) |
| δ(NH) | 1543 | 1546 (Δ = -3) | 1596 (Δ = -53) |
| υ(C-N) | 1304 | 1248 (Δ = +56) | 1247 (Δ = +57) |
| $ρ_w(NH_2)$ | 778 | 769 (Δ = +9) | 735 (Δ = +43) |

**Table S8.** Comparison of experimental vibrations of **VAL/NIC LAG 60 min.** with calculated frequencies.

| Assignment | Observed frequencies [$cm^{-1}$] | Calculated frequencies [$cm^{-1}$] | |
|---|---|---|---|
| | VAL/NIC LAG 60 min. | VAL/NIC 1 | VAL/NIC 2 |
| υ(OH) | - | - | 3619 (-) |
| $υ(NH)^{tetrazole}$ | 3443 | 3508 (Δ = -65) | 3227 (Δ = +216) |
| **$υ(C=O)^{acid}$** | **1717** | **1686 (Δ = +31)** | **1751 (Δ = -34)** |
| **$υ(C=O)^{amide}$** | **1605** | **1647 (Δ = -42)** | **1624 (Δ = -19)** |
| υ(C-O) | 1391 | 1204 (Δ = +187) | 1073 (Δ = +318) |
| $υ(NH_2)^{as}$ | 3366 | 3565 (Δ = -199) | 3564 (Δ = -198) |
| $υ(NH_2)^{s}$ | 3182 | 3278 (Δ = -96) | 3340 (Δ = -158) |
| **υ(C=O)** | **1681** | **1646 (Δ = +35)** | **1663 (Δ = +18)** |
| δ(NH) | 1568 | 1569 (Δ = -1) | 1596 (Δ = -28) |
| δ(NH) | 1517 | 1546 (Δ = -29) | 1596 (Δ = -79) |
| υ(C-N) | 1342 | 1248 (Δ = +94) | 1247 (Δ = +95) |
| $ρ_w(NH_2)$ | 778 | 769 (Δ = +9) | 735 (Δ = +43) |

**Table S9.** Comparison of experimental vibrations of **VAL/NIC ball mill** with calculated frequencies.

| Assignment | Observed frequencies [$cm^{-1}$] | Calculated frequencies [$cm^{-1}$] | |
|---|---|---|---|
| | VAL/NIC ball mill | VAL/NIC 1 | VAL/NIC 2 |
| υ(OH) | - | - | 3619 (-) |
| $υ(NH)^{tetrazole}$ | 3352 | 3508 (Δ = -156) | 3227 (Δ = +125) |
| **$υ(C=O)^{acid}$** | **1716** | **1686 (Δ = +30)** | **1751 (Δ = -35)** |
| **$υ(C=O)^{amide}$** | **1622** | **1647 (Δ = -25)** | **1624 (Δ = -2)** |
| υ(C-O) | 1390 | 1204 (Δ = +186) | 1073 (Δ = +317) |
| $υ(NH_2)^{as}$ | 3352 | 3565 (Δ = -213) | 3564 (Δ = -212) |
| $υ(NH_2)^{s}$ | 3192 | 3278 (Δ = -86) | 3340 (Δ = -148) |
| **υ(C=O)** | **1675** | **1646 (Δ = +29)** | **1663 (Δ = +12)** |
| δ(NH) | 1568 | 1569 (Δ = -1) | 1596 (Δ = -28) |
| δ(NH) | 1516 | 1546 (Δ = -30) | 1596 (Δ = -80) |
| υ(C-N) | 1355 | 1248 (Δ = +107) | 1247 (Δ = +108) |
| $ρ_w(NH_2)$ | 778 | 769 (Δ = +9) | 735 (Δ = +43) |

## 5.3. Quantum Theory of Atoms in Molecules (QTAIM)

**Table S10.** Topological parameters corresponding to H-bonds involved in intermolecular interactions.

| D-H···A | $\rho_{BCP}$ | $\nabla^2\rho_{BCP}$ | $G_{BCP}$ | $V_{BCP}$ | $H_{BCP}$ | $K_{BCP}$ | $E_{bin}$ [kcal/mol] | $\Delta r_H + \Delta r_A$ | $\Delta r_H - \Delta r_A$ |
|---|---|---|---|---|---|---|---|---|---|
| **Calculated in gas phase** | | | | | | | | | |
| **VAL/NIC1** | | | | | | | | | |
| O2-H2···O4 | 0.0514 | 0.1413 | 0.0427 | -0.0500 | -0.0073 | 0.0073 | -15.68 | 1.08 | 0.28 |
| N3-H3a···O3 | 0.0271 | 0.0967 | 0.0223 | -0.0204 | 0.0019 | -0.0019 | -6.40 | 0.81 | 0.25 |
| **VAL/NIC2** | | | | | | | | | |
| N1-H1···O4 | 0.0316 | 0.1229 | 0.0290 | -0.0273 | 0.0017 | -0.0017 | -8.56 | 0.93 | 0.23 |
| N3-H3a···O1 | 0.0231 | 0.0938 | 0.0204 | -0.0174 | 0.0030 | -0.0030 | -5.45 | 0.78 | 0.22 |
| **VAL/NIC1 + EtOH** | | | | | | | | | |
| O2-H2···O4 | 0.0507 | 0.1408 | 0.0421 | -0.0490 | -0.0069 | 0.0069 | -15.36 | 1.08 | 0.27 |
| N3-H3a···O3 | 0.0274 | 0.0976 | 0.0226 | -0.0207 | 0.0018 | -0.0018 | -6.49 | 0.81 | 0.25 |
| O5-H5···N2 | 0.0195 | 0.0656 | 0.0141 | -0.0119 | 0.0023 | -0.0023 | -3.72 | 0.60 | 0.30 |
| **Calculated in ethanol** | | | | | | | | | |
| **VAL/NIC1** | | | | | | | | | |
| O2-H2···O4 | 0.0535 | 0.1436 | 0.0443 | -0.0528 | -0.0084 | 0.0084 | -16.56 | 1.09 | 0.27 |
| N3-H3a···O3 | 0.0241 | 0.0858 | 0.0194 | -0.0173 | 0.0021 | -0.0021 | -5.42 | 0.75 | 0.25 |
| **VAL/NIC2** | | | | | | | | | |
| N1-H1···O4 | 0.0360 | 0.1333 | 0.0330 | -0.0326 | 0.0003 | -0.0003 | -10.22 | 0.97 | 0.23 |
| N3-H3a···O1 | 0.0233 | 0.0948 | 0.0207 | -0.0177 | 0.0030 | -0.0030 | -5.55 | 0.79 | 0.23 |

[a] $\Delta r_H = r_H^V - r_H$; $\Delta r_A = r_A^V - r_A$

**Table S11.** Geometrical parameters corresponding to H-bonds involved in intermolecular interactions along with molecular graphs with the BCPs (3, -1) as orange dots.

| Designed system | D-H···A | D-H [Å] | D···A [Å] | H···A [Å] | D-H···A [°] |
|---|---|---|---|---|---|
| **Calculated in gas phase** | | | | | |
| **VAL/NIC1** | O2-H2···O4 | 1.006 | 2.642 | 1.638 | 175.234 |
| | N3-H3a···O3 | 1.022 | 2.911 | 1.909 | 165.671 |
| **VAL/NIC2** | N1-H1···O4 | 1.027 | 2.809 | 1.794 | 168.946 |
| | N3-H3a···O1 | 1.019 | 2.915 | 1.936 | 160.112 |
| **VAL/NIC1 + EtOH** | O2-H2···O4 | 1.006 | 2.646 | 1.642 | 175.332 |
| | N3-H3a···O3 | 1.022 | 2.906 | 1.904 | 165.816 |
| | O5-H5···N2 | 0.969 | 2.986 | 2.100 | 151.243 |
| **Calculated in ethanol** | | | | | |
| **VAL/NIC1** | O2-H2···O4 | 1.009 | 2.627 | 1.623 | 172.639 |
| | N3-H3a···O3 | 1.020 | 2.960 | 1.962 | 165.225 |
| **VAL/NIC2** | N1-H1···O4 | 1.033 | 2.764 | 1.747 | 167.435 |
| | N3-H3a···O1 | 1.020 | 2.921 | 1.930 | 163.045 |

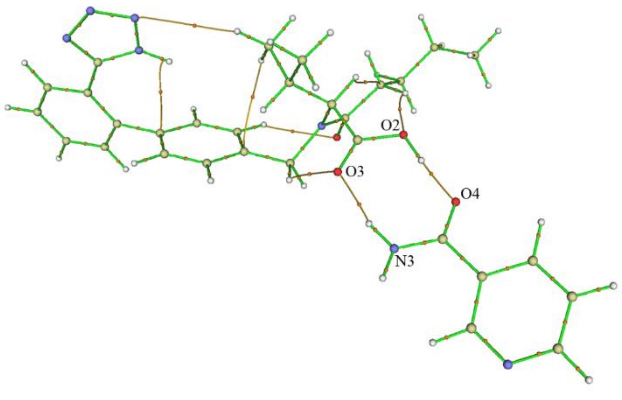


**VAL/NIC1**

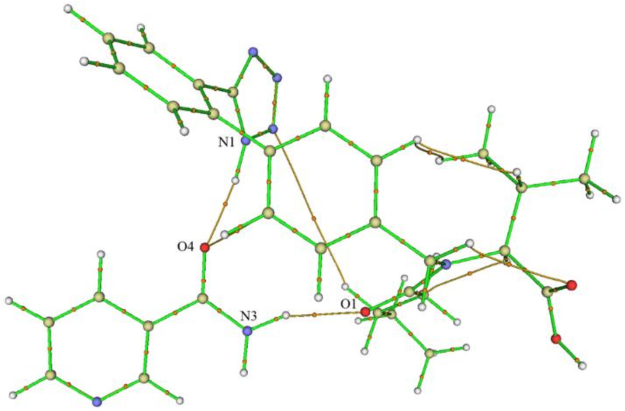


**VAL/NIC2**

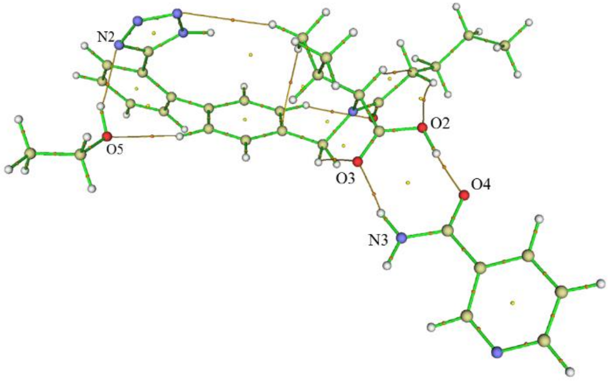


**VAL/NIC1 + EtOH**

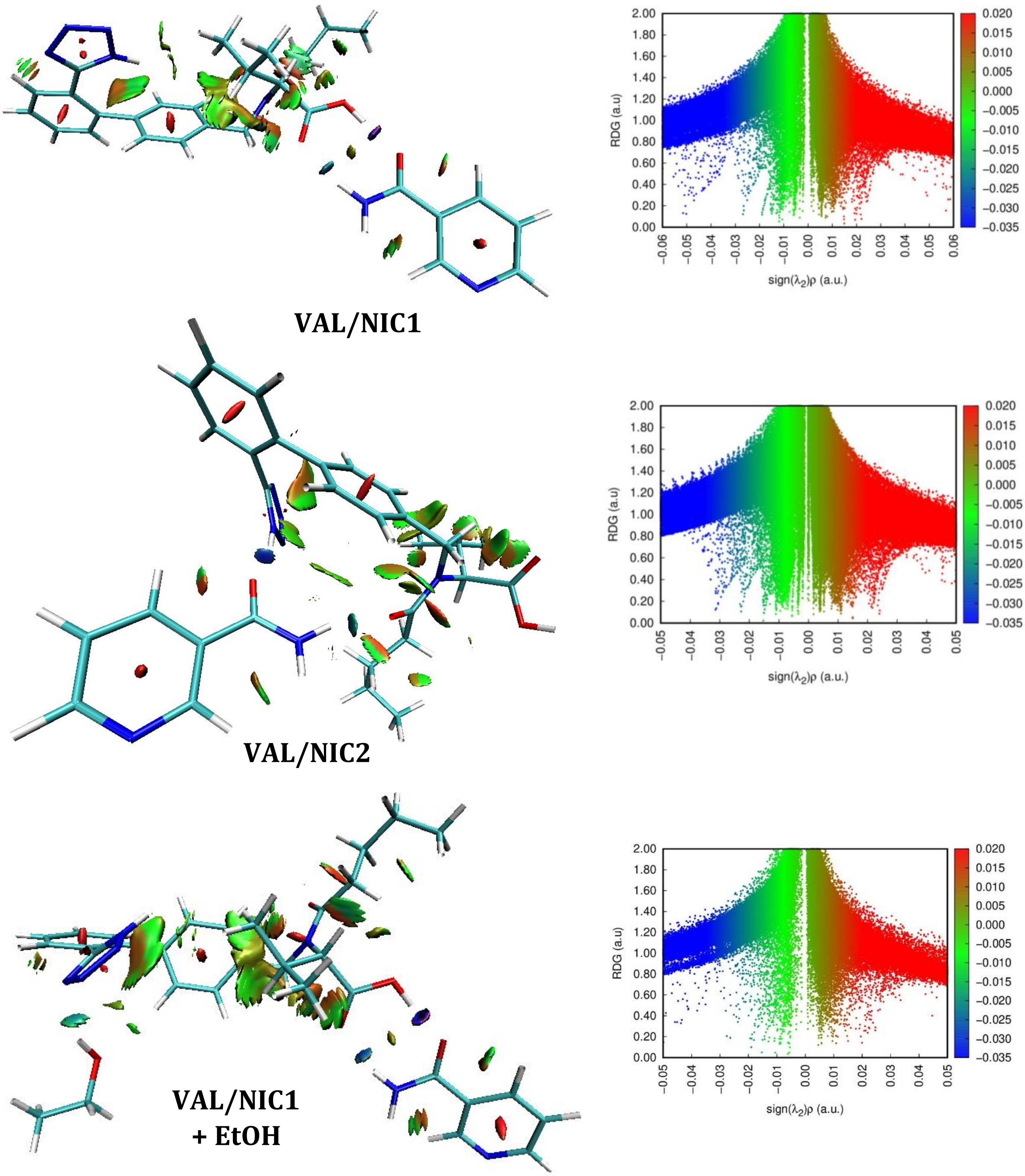


**Fig. S14a.** 3D-NCI plot with color-filled reduced density gradient (RDG) isosurfaces depicting non-covalent interactions in VAL/NIC1 and VAL/NIC2 along with 2D-NCI scatter plots (calculated in gas phase).

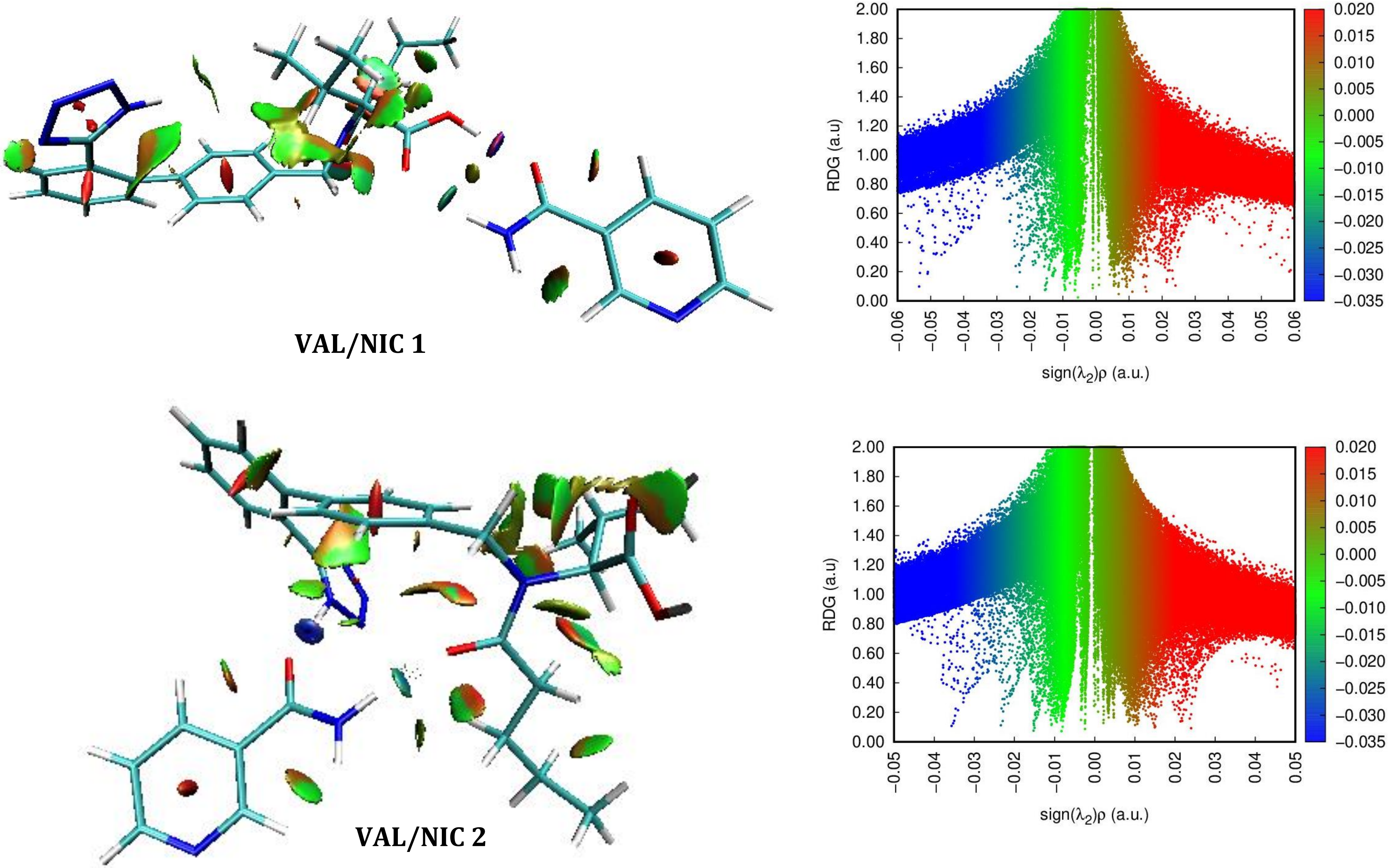


**Fig. S14b.** 3D-NCI plot with color-filled reduced density gradient (RDG) isosurfaces depicting non-covalent interactions in VAL/NIC1 and VAL/NIC2 along with 2D-NCI scatter plots (calculated in ethanol).

**Table S12.** Topological parameters corresponding to H-bonds involved in intramolecular interactions in homodimers/trimers.

| System | D-H···A | $\rho_{BCP}$ | $\nabla^2\rho_{BCP}$ | $G_{BCP}$ | $V_{BCP}$ | $H_{BCP}$ | $K_{BCP}$ | $E_{bin}$ [kcal/mol] | $\Delta r_H + \Delta r_A$ | $\Delta r_H - \Delta r_A$ |
|---|---|---|---|---|---|---|---|---|---|---|
| **VAL/VAL1** | N1'-H1'···O1 (i) | 0.03176 | 0.1495 | 0.03362 | -0.02986 | 0.003757 | -0.003757 | **-9.36** | 0.90 | 0.20 |
| **VAL/VAL2** | O2-H2···N2' (ii) | 0.02674 | 0.1021 | 0.02337 | -0.02121 | 0.002160 | -0.002160 | **-6.65** | 0.73 | 0.27 |
| **VAL/VAL3** | N1'-H1'···O1 (iii) | 0.04425 | 0.1497 | 0.04010 | -0.04277 | -0.002667 | 0.002667 | **-13.41** | 1.01 | 0.25 |
| | O2'-H2'···O5' *(iv) | 0.03490 | 0.1472 | 0.03536 | -0.03390 | 0.001451 | -0.001451 | **-10.63** | 0.94 | 0.24 |
| | O5-H5···N2' *(v) | 0.02171 | 0.0083 | 0.01807 | -0.01547 | 0.002595 | -0.002595 | **-4.85** | 0.68 | 0.26 |
| **NIC/NIC** | N3-H3a···O4' (vi) | 0.02224 | 0.0699 | 0.01561 | -0.01372 | 0.001886 | -0.001886 | **-4.30** | 0.67 | 0.28 |
| | N3-H3b···N5'' (v) | 0.02152 | 0.0921 | 0.01956 | -0.01611 | 0.003456 | -0.003456 | **-5.05** | 0.78 | 0.21 |

[a] $\Delta r_H = r_H^V - r_H$; $\Delta r_A = r_A^V - r_A$; * interaction with ethanol (solvated form of VAL crystal);
Symmetry codes: (i) 1+x, y, z; (ii) 1.5-x, 2-y, 1/2+z; (iii) 1-x, 1/2+y, 1/2-z; (iv) -1/2+x, 1/2-y, -z; (v) 1+x, y, z; (vi) x, 1/2-y, -1/2+z; (v) 1+x, 1/2-y, 1/2+z;

**Table S13.** Geometrical parameters determined from X-ray structures, corresponding to H-bonds involved in intramolecular interactions in homodimers/trimers.

| Designed system | D-H···A | D-H [Å] | D···A [Å] | H···A [Å] | D-H···A [°] |
|---|---|---|---|---|---|
| **VAL/VAL 1** | N1'-H1'···O1 (i) | 0.859 | 2.645 | 1.818 | 160.76 |
| **VAL/VAL 2** | O2-H2···N2' (ii) | 0.820 | 2.712 | 1.985 | 147.37 |
| **VAL/VAL3** | N1'-H1'···O1 (iii) | 0.958 | 2.670 | 1.713 | 176.77 |
| | O2'-H2'···O5' *(iv) | 0.840 | 2.619 | 1.783 | 173.43 |
| | O5-H5···N2' *(v) | 0.840 | 2.885 | 2.065 | 165.15 |
| **NIC/NIC** | N3-H3a···O4' (vi) | 1.011 | 3.075 | 2.076 | 169.31 |
| | N3-H3b···N5'' (v) | 1.011 | 2.960 | 1.953 | 174.65 |

* interaction with ethanol (solvated form of VAL crystal); Symmetry codes: (i) 1+x, y, z; (ii) 1.5-x, 2-y, 1/2+z; (iii) 1-x, 1/2+y, 1/2-z; (iv) -1/2+x, 1/2-y, -z; (v) 1+x, y, z; (vi) x, 1/2-y, -1/2+z; (v) 1+x, 1/2-y, 1/2+z;

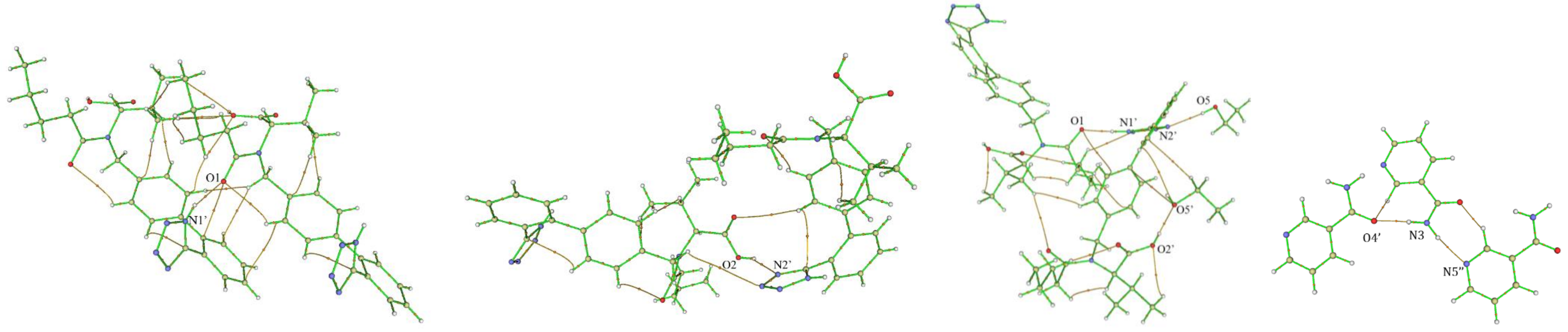


**VAL/VAL 1** **VAL/VAL 2** **VAL/VAL3** **NIC/NIC**

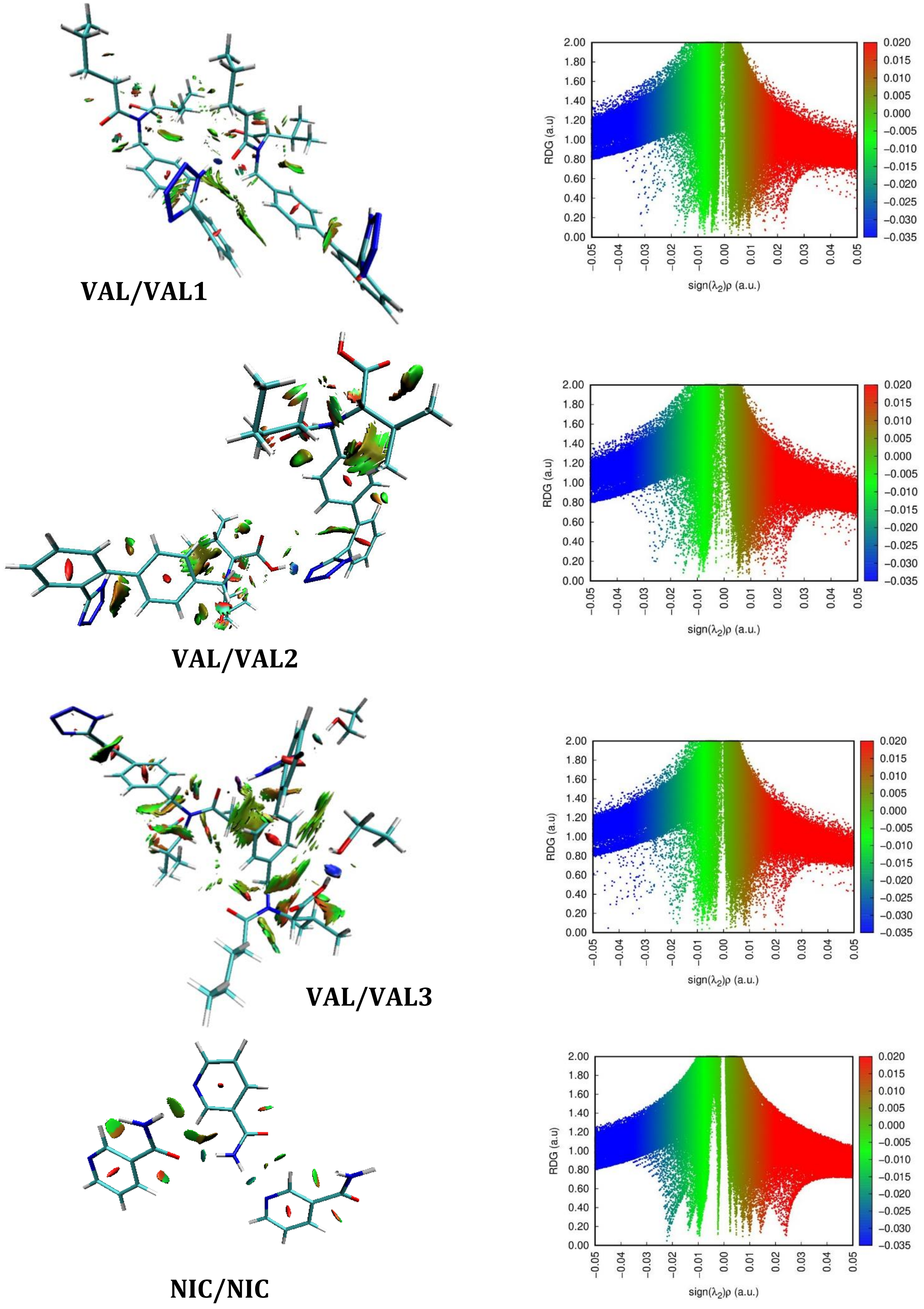


**Fig. S15.** 3D-NCI plot with color-filled reduced density gradient (RDG) isosurfaces depicting non-covalent interactions in homodimers/trimers along with 2D-NCI scatter plots.
